# Cytotoxic Curvalarol C and Other Compounds from Marine Fungus *Asteromyces cruciatus* KMM 4696

**DOI:** 10.3390/molecules30244772

**Published:** 2025-12-14

**Authors:** Liliana E. Nesterenko, Ekaterina A. Yurchenko, Olesya I. Zhuravleva, Galina K. Oleinikova, Natalya N. Kirichuk, Roman S. Popov, Viktoria E. Chausova, Konstantin A. Drozdov, Ekaterina A. Chingizova, Marina P. Isaeva, Anton N. Yurchenko

**Affiliations:** G.B. Elyakov Pacific Institute of Bioorganic Chemistry, Far Eastern Branch of the Russian Academy of Sciences, 159 Prospect 100-letiya Vladivostoka, Vladivostok 690022, Russia; nesterenko_le@piboc.dvo.ru (L.E.N.); eyurch@piboc.dvo.ru (E.A.Y.); zhuravleva_oi@piboc.dvo.ru (O.I.Z.); drovsh@yandex.ru (K.A.D.); issaeva@piboc.dvo.ru (M.P.I.)

**Keywords:** *Asteromyces cruciatus*, OSMAC, phylogeny, anthraquinones, cytotoxicity, anticancer activity

## Abstract

The present study aimed to isolate new specialized metabolites from the obligate marine fungus *Asteromyces cruciatus* KMM 4696. The strain KMM 4696 was identified based on the 28S rRNA, ITS, and TEF1 molecular genetic markers. Chromatographic separation of the fungal extract obtained from KI-containing nutrient media cultivation led to the isolation of undescribed pentanorlanostanes curvalarols C (**1**) and D (**2**), as well as an undescribed 6/6/5 anthraquinone acruciquinone D (**3**), along with eight known metabolites. The structures of the isolated compounds were established based on 1D and 2D NMR and MS data. The cytotoxic activity of curvalarol C (**1**) was assessed in MCF-10A and MCF-7 cells. Curvalarol C exhibited selective activity against cancer MCF-7 cells and inhibiting colony formation with an IC_50_ of 4.7 µM.

## 1. Introduction

Specialized metabolites from marine microorganisms have the potential to be a source of novel compounds for drug development. Chemical studies on microorganisms aim to identify structurally active compounds in culture media and mycelia. The “One Strain – Many Compounds” (OSMAC) strategy is used to obtain various specialized metabolites from one strain and involves changing a variety of factors: the form of cultivation vessel, temperature, medium composition, pH, light, and salinity can stimulate the production of new specialized metabolites [1].

One option for changing the medium composition is the addition of halide salts. The marine fungal strain *Trichoderma* sp. TPU199 was cultivated by the addition of NaBr, NaI, and NaF to the culture medium to investigate the biosynthesis of halogenated metabolites. This study showed that the addition of fluoride ions to the culture medium resulted in a significant reduction in fungal growth as well as the secondary metabolites production. When NaBr or NaI was added to the medium, two derivatives of pretrichodermamide A, DC1149R and new iododithiobrevamide, were isolated [2]. The marine fungal strain *Aspergillus unguis* CRI282-03 was cultured in a culture medium supplemented with KBr, KI, or KF. This fungal strain did not grow in the presence of fluoride ions in the medium; however, when KBr was added to the medium, three new brominated depsidones, aspergillusidones D–F, two new orcinol derivatives, aspergillusphenols A and B, and three known compounds were isolated. When KI was added to the medium, one new depsidone, 2,4-dichlorounguinol, and five known compounds were isolated [3]. In another study, the fungal strain *Aspergillus* sp. SCSIO F063 was cultured with the addition of NaBr, leading to the production of two new brominated anthraquinones, a new nonhalogenated anthraquinone, and five known compounds [4]. The fungal strain *Dothideomycetes* sp. CRI7 grown in the presence of KBr produced new polyketides, calbistrins I-K, dothideomynones D–F, and five known compounds [5]. The same strain, grown on a medium with KI, produced a new polyketide, dothideomynone E, along with a known austdiol [5].

As of 2023, marine fungi have been recorded in nine phyla, 33 classes, 107 orders, 273 families, 767 genera and comprise 1898 species including several obligate species, such as *Asteromyces cruciatus,* which is widespread in marine environments, but its taxonomic position remains uncertain [6]. Recently, the strain *A. cruciatus* 763 was shown to yield a new pentapeptide, lajollamide A, as well as the known compounds regiolone, hyalodendrin, gliovictin, 1N-norgliovicitin, and bis-N-norgliovictin [7]. Using the OSMAC approach, two new polyketides, primarolides A and B, were isolated from another fungus *A. cruciatus* fermented with the epigenetic modifier suberoylanilide hydroxamic acid (SAHA) and/or NaCl [8]. Previously, we discovered that the marine fungus *Asteromyces cruciatus* KMM 4696 produces six novel polyketides, acrucipentyns A–F [9], new anthraquinones, acruciquinones A–C [10], nine known polyketide derivatives, and gliovictin.

The aim of the present study was to isolate, elucidate the structure, and study the cytotoxicity of specialized metabolites of the marine fungus *Asteromyces cruciatus* KMM 4696 cultivated on a potassium iodide-containing culture medium.

## 2. Results and Discussion

### 2.1. Molecular Identification of the Strain KMM 4696

The strain KMM 4696 was identified using molecular markers such as 28S rRNA, internal transcribed spacer (ITS) region, and translation elongation factor 1 (TEF1). Fragments of 28S rRNA gene of about 950 bp in length, ITS region of about 1300 bp in length, and TEF1 gene of about 300 bp in length were amplified and sequenced. According to the BLAST (version 2.17.0) results, the corresponding sequences were 100% identical to those of the ex-type strain *Asteromyces cruciatus* CBS 171.63T. The genus *Asteromyces* is represented by one species, *Asteromyces cruciatus* [11] and was recently determined as a member of the family Pleosporaceae [12].

**Figure 1:**
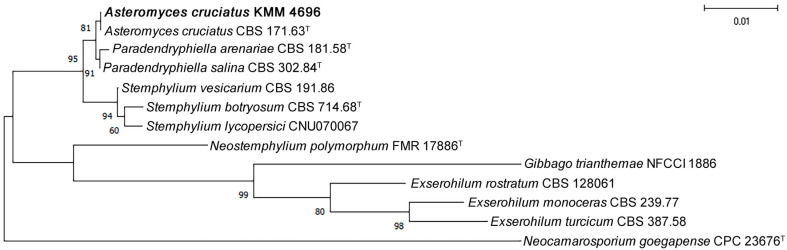
NJ tree based on ITS sequences showing phylogenetic position of the strain KMM 4696 among related representatives of the genera *Exserohilum*, *Gibbago*, *Stemphylium*, *Paradendryphiella*, *Asteromyces* and *Neostemphylium* belonging to the *Pleosporaceae* family. Bootstrap values (%) of 500 replications and nodes with confidence values greater than 60% are indicated. The scale bars represent 0.01 substitutions per site.

Phylogenetic NJ tree based on ITS sequences clearly showed that the strain KMM 4696 clusters with the ex-type strain *Asteromyces cruciatus* CBS 171.63^T^ (Figure 1). It is known that the genus *Asteromyces* is a monotypic one represented by one species, *Asteromyces cruciatus*, which had an uncertain taxonomic position among the *Ascomycota*. To date, the genus *Asteromyces* together with representatives of the genera *Paradendryphyella* and *Stemphylium* belong to the family *Pleosporaceae*. However, the ML phylogenetic tree based on the combined ITS, LSU, *rpb2*, *tef1*, and *gapdh* loci showed a low resolution between the clades [12]. The use of ITS allowed us to more accurately determine the taxonomic position of KMM 4696 based on high bootstrap support for the separation of the genera *Asteromyces, Paradendryphyella* and *Stemphylium*. Furthermore, comparison of concatenated (ITS, LSU, *tef1*) sequences shows 100% nucleotide identity of KMM 4696 with the ex-type strain of the genus *Asteromyces* (Figure S54).

### 2.2. The Isolation and Structure Elucidation of Individual Compounds from A. cruciatus KMM 4696 Fermented with KI

**Figure 2:**
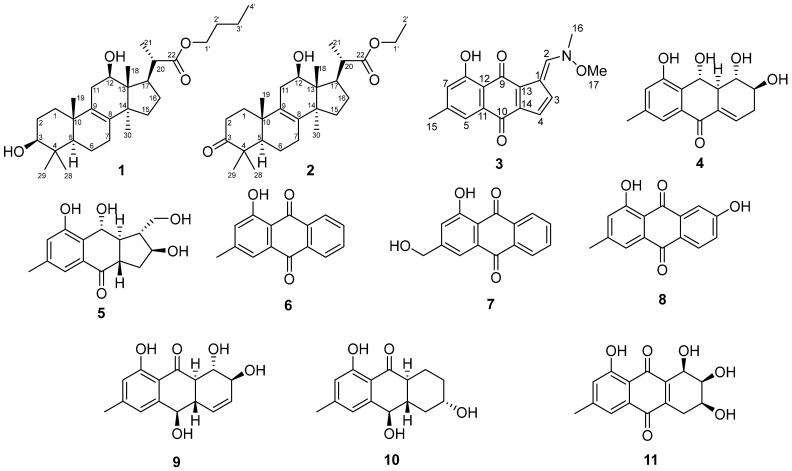
The compounds isolated from *Asteromyces cruciatus* KMM 4696 cultured with KI.

Previously undescribed curvalarol C (**1**), curvalarol D (**2**), and acruciquinone D (**3**), as well as the known anthraquinone derivatives acruciquinones A (**4**) and C (**5**) [10], pachybasin (**6**) [13], ω-hydroxypachybasin (**7**) [14,15], 1,7-dihydroxy-3-methyl-9,10-anthraquinone (**8**) [16], rubrumol (**9**) [17], coniothyrinone B (**10**) [18], and pleosporone (**11**) [19] (Figure 2), were isolated from the culture of *A*. *cruciatus* KMM 4696 cultured with KI. All known compounds were identified by comparing the NMR and HRESIMS data with literature data.

**Figure 3:**
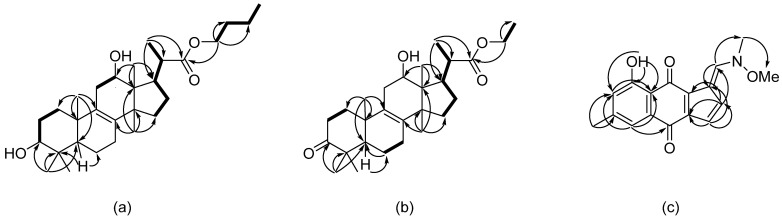
Key ^1^H–^1^H COSY correlations of curvalarol C (**1**) (**a**), curvalarol D (**2**) (**b**), acruciquinone D (**3**) (**c**), and ^1^H–^13^C HMBC correlations of 1 (**a**) and 2 (**b**).

The molecular formula of **1** was determined to be C_29_H_48_O_4_ based on the analysis of the (+)-HRESIMS (Figure S46) containing the peak of the cationized molecule [M + Na]^+^ (*m*/*z* 483.3445), which was confirmed by the ^13^C NMR data. Analysis of the ^1^H and ^13^C NMR spectra of compound **1** (Table 1, Figures S5–S11) revealed five singlets, one doublet, and one triplet methyl group, ten *sp^3^*-methylene groups including one oxygen-bearing, five *sp^3^*-methine protons including two oxygen-bearing, four quaternary *sp^3^*-carbons, as well as a tetrasubstituted double bond and a carboxyl group. The NMR data of **1** were generally similar to those of the known metabolite of the soil fungus *Curvularia borreriae* HS-FG-237, curvalarol A [20] with the most differences in the C-22 and C-20 chemical shifts. The COSY correlations (Figure 3a) revealed the spin system CH_2_-1′–CH_2_-2′–CH_2_-3′–CH_3_-4′, whereas the HMBC correlations (Figure 3a) from H_2_-1′ (δ_H_ 4.01) to C-22, C-2′ (δ_C_ 31.5), and C-3′ (δ_C_ 19.9) elucidated the OBu substituent at C-22 in **1** instead of OMe in curvalarol A [20]. Thus, compound **1** was named curvalarol C.

The relative stereochemistry of **1** was suggested to be the same as that of curvalarol A and was confirmed by ROESY correlations: H_3_-19/H_3_-18 and H_3_-29, H_3_-30/H-17 (δ_H_ 2.10), H-12 (δ_H_ 4.08), H_3_-18/H-20 (δ_H_ 2.65), and H_3_-28/H-3 (δ_H_ 3.14). Unfortunately, curvalarol C (**1**) was degraded after obtaining the NMR spectra, and we were unable to synthesize MTPA derivatives to determine the absolute stereochemistry using Mosher’s method or to obtain ECD spectra for quantum-chemical calculations. Thus, the absolute stereoconfigurations of curvalarol C (**1**) were suggested to be 3*S*, 5*R*, 10*S*, 12*R*, 13*R*, 14*S*, 20*R*, and 21*S*, based on the evident biogenetic relationships with curvalarol A and other pentanorlanostanoids (Table S1).

The molecular formula of **2** was determined to be C_27_H_42_O_4_ based on the analysis of the (+)-HRESIMS (Figure S47) containing the peak of the cationized molecule [M + Na]^+^ (*m*/*z* 453.2976), which was confirmed by the ^13^C NMR data. A close inspection of the ^1^H and ^13^C NMR data of compound **2** (Table 1, Figures S12–S16) indicated a high similarity with those of 1. The COSY and HMBC data (Figure 3b) revealed the OEt group at C-22 in **2** instead of the OBu group in **1**, as well as the C-3 keto group in **2** instead of the hydroxymethine group in **1**. Thus, compound **2** was named curvalarol D.

It should be noted that despite the use of ethanol during extraction and chromatographic separation, the formation of curvalarol D (**2**) as a result of artifact ethylation is unlikely. There are numerous examples of triterpenoids with free carboxyl groups that were isolated under similar conditions using ethanol, without the corresponding O-ethyl derivatives [21,22]. To verify the native origin of compounds **1** and **2**, their corresponding peaks were searched for in the HPLC-MS chromatogram of the crude EtOAc extract. Curvularols C (**1**) and D (**2**) have been assigned with peaks at 17.9 (*m*/*z* 483.3389), 17.3 (*m*/*z* 431.3124) based on their exact mass value and fragmentation patterns with those for isolated compounds **1** and **2** (Table S2). Therefore, we believe that compounds **1** and **2** are of natural origin.

At this time, only 15 pentanorlanostane-type compounds were isolated, most of which were of fungal origin, and their structures and sources are summarized in Table S2. Curvalarol A and curvalarol B were isolated from soil fungus *Curvularia borreriae* HS-FG-237 [20]; dendryphiellide A was isolated from marine fungus *Paradendryphiella salina* PC 362H [23]; 23,24,25,26,27-pentanorlanost-8-ene-3β,22-diol and pentanorlanost-7,9(11)-diene acids were isolated from entomopathogenic fungus *Verticillium lecanii* [24], soil fungus *Curvularia borreriae* HS-FG-237 [20], and dematiaceous fungus *Cladosporium* sp. IFM 49189 [25], cladosporides A-D, dihydrocladosporide A, and 3,30-dioxo23,24,25,26,27-pentanorlanost-8-en-22-oic acid were isolated from the dematiaceous fungus *Cladosporium* sp. IFM 49189 [26] and ganosineniol A were isolated from the medicinal fungus *Ganoderma sinense* [27]. Moreover, cucumariosides I_4_, A_10_, and G_2_ have been isolated from the sea cucumber *Eupentacta fraudatrix* [28,29,30].

The molecular formula of **3** was determined to be C_17_H_15_NO_4_ based on the analysis of the (+)-HRESIMS (Figure S48) containing the peak of the cationized molecule [M + H]^+^ (*m*/*z* 298.1073), which was confirmed by ^13^C NMR data. In the ^1^H and ^13^C NMR (700MHz, acetone-d_6_) spectra of compound **3** (Table 1, Figures S17–S22) contain signals of three methyl groups (δ_C_ 21.8, δ_H_ 2.39; δ_C_ 53.9, δ_H_ 3.95; δ_C_ 61.3, δ_H_ 3.95), five *sp^2^*-methine groups (δ_C_ 161.0, δ_H_ 8.40; δ_C_ 133.7, δ_H_ 6.99; δ_C_ 119.2, δ_H_ 6.78; δ_C_ 120.5, δ_H_ 7.43; δ_C_ 123.5, δ_H_ 6.94;), six quaternary *sp^2^*-carbons (δ_C_ 120.4, 147.0, 136.0, 115.7, 124.2, 137.8), as well as an oxygen-bearing *sp^2^*-carbon (δ_C_ 163.1) and two quinone keto-groups (δ_C_ 184.6, 181.2).

The HMBC correlations (Figure 3c) from H-5 (δ_H_ 7.43) to C-7 (δ_C_ 123.5), C-10 (δ_C_ 181.2), and C-12 (δ_C_ 115.7), from H-7 (δ_H_ 6.94) to C-5 (δ_C_ 120.5) and C-12, from H_3_-15 (δ_H_ 21.8) to C-5, C-6 (δ_C_ 147.0), and C-7, from 8-OH (δ_H_ 12.82) to C-7, C-8 (δ_C_ 163.1), and C-12, from H-3 (δ_H_ 6.99) to C-2 (δ_C_ 161.0), C-13 (δ_C_ 124.2), and C-14 (δ_C_ 137.8), from H-4 (δ_H_ 6.78) to C-1 (δ_C_ 120.4), C-3 (δ_C_ 133.7), C-13, and C-14, from H-2 (δ_H_ 8.40) to C-1, C-3, and C-13 revealed the structure of anthraquinone derivative with 6/6/5 cyclic core with methyl group at C-6, hydroxy-group at C-8, and exocyclic double bond Δ^1,2^. The HMBC correlations from H-2 (δ_H_ 8.40) to C-16 (δ_C_ 53.9) and from H-16 (δ_H_ 3.95) to C-17 (δ_C_ 61.3), together with downfield chemical shifts of *sp^2^*-methine C-2 and methyl groups C-16 and C-17, as well as the presence of one nitrogen atom and one more oxygen atom according to HRESIMS data, suggested the presence of an amino group at C-2 with an N-methyl group at C-16 and an N-methoxyl group at C-17. Compound **3**, named acruciquinone D, is the second example of a natural 6/6/5 anthraquinone derivative, following acruciquinone C (**5**) [10].

### 2.3. Bioactivity of Curvalarol C (**1**)

The biological activities of new curvalarol D (**2**) and acruciquinone D (**3**) were not studied because of their insufficient amounts.

The effect of curvalarol C (**1**) on the growth of *Staphylococcus aureus*, *Escherichia coli*, and *Candida albicans* was investigated, and compound **1** was found to be inactive.

**Figure 4:**
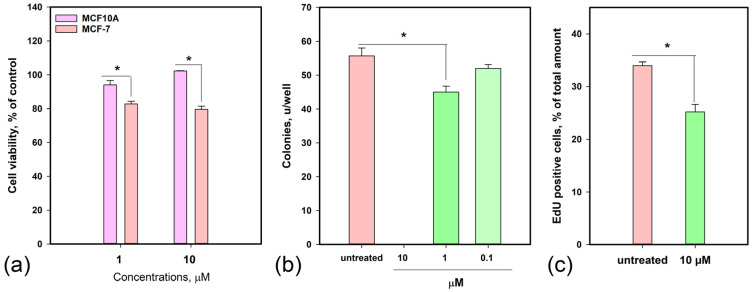
Effects of curvalarol C (**1**) on cell viability (**a**), MCF-7 cell colony formation (**b**), and proliferation (**c**). Data are presented as mean ± SEM. * Asterisks indicate significant differences (*p* ≤ 0.05).

The cytotoxic activity of curvalarol C (**1**) was assessed in normal human breast epithelial MCF-10A and breast cancer MCF-7 cells. Curvalarol C (**1**) inhibited the viability of MCF-10A and MCF-7 cells after 48 h with IC_50_s of 76.3 and 53.9 µM, respectively. Moreover, compound **1** at 1 and 10 µM was more toxic to MCF-7 than to MCF-10A cells (Figure 4a).

The effect of **1** on the formation of MCF-7 colonies was studied, and the data are presented in Figure 4b. Thus, **1** fully inhibited MCF-7 cell colony formation at 10 µM and by 14.3% at 1 µM; the IC_50_ was calculated as 4.7 µM.

The effect of **1** on MCF-7 cell proliferation was assessed using an EdU incorporation assay (Figure 4c). The untreated cell population had 33.9% EdU-positive cells, and **1** at 10 µM decreased the percentage of EdU-positive cells to 25.2%.

**Figure 5:**
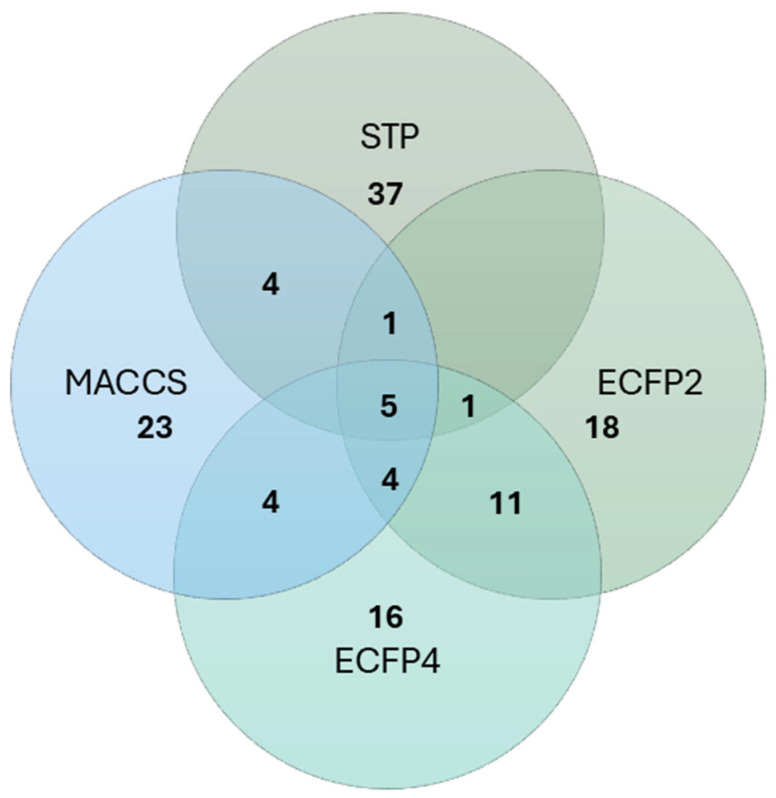
Prediction of curvalarol C (**1**) targets. STP—SwissTargetPrediction model; ECFP2, ECFP4 and MACCS—Target.Net models.

The prediction of possible curvalarol C (**1**) targets was evaluated using the SwissTargetPrediction (http://swisstargetprediction.ch/index.php, accessed on 18 September 2025) and TargetNet (http://targetnet.scbdd.com/home/index/, accessed on 18 September 2025) online services. The SwissTargetPrediction model uses 2D and 3D similarity parameters in a so-called Combined-Score [31]. TargetNet models using different types of fingerprints, and we used ECFP2, ECFP4, and MACCS fingerprints for target prediction [32].Therefore, four lists of the predicted top-50 targets were obtained and analyzed (Figure 5). A list of the predicted targets is available in the Supplementary Materials.

All models predicted five targets such as androgen receptor, nitric oxide synthase, inducible, cannabinoid receptor 2, acetylcholinesterase, and tyrosine-protein phosphatase non-receptor type 1. SwissTargetPrediction and ECFP2 and ECFP4 models predicted interactions with the glucocorticoid receptor. SwissTargetPrediction and ECFP2, MACCS models predicted interactions with estrogen receptor alpha. ECFP2, ECFP4, and MACCS models predicted as targets 3-oxo-5-alpha-steroid 4-dehydrogenase 2, prostaglandin F2-alpha receptor, retinoic acid receptor RXR-beta, and prostacyclin receptor. Moreover, the SwissTargetPrediction and MACCS models predicted interactions with progesterone and mineralocorticoid receptors, estrogen receptor beta, and protein kinase C alpha type.

Thus, steroid receptors, prostaglandin receptors and some others may be targeted by curvalarol C (**1**). Really, steroid receptors are possible targets for pentanorlanostane compounds due to structure similarity with steroid ligands of these receptors, i.e., estradiol, cortisol and others. Recently we proposed that some oxygenated sterol derivatives from marine sponge may interact with ligand-binding domain of glucocorticoid receptor [33] while 3β,15β-dihydroxy-(22*E*,24*R*)-ergosta-5,8(14),22-trien-7-one from marine fungus can act as estrogen-dependent cardioprotective agent [34]. So, the targeting of curvalarol C (1) on steroid receptors will be verified in future studies.

Some other pentanorlanostane triterpenes also had cytotoxic activity against breast cancer cells. Curvalarol A and curvalarol B exhibited in vitro cytotoxicity against the A549, K562 and MDA-MB-231 cell lines with IC_50_ values of 15–25 μg/mL [20]. Dendryphiellide A exhibited a weak activity against MCF-7 (IC_50_ = 50 mkM), MCF7-Sh-WISP2 (IC_50_ > 60 mkM), 3T3-F442A (IC_50_ > 60 mkM) cells [23]. The activity of ganosineniol A was tested using HL-60, SMMC-7721, A-549, MCF-7, and SW480 cell lines and IC_50_s were more than 40 µM [27]. Cucumarioside A_10_ showed moderate cytotoxic activity against mouse Ehrlich cells (which is spontaneous breast mouse carcinoma) with ED_50_ 25 mkg/mL [33].

Thus, all the described compounds of this group, for which cytotoxic activity has been studied, have moderate or weak effects. For the first time, we not only studied the cytotoxic effect of curvalarol C (**1**), but also showed its effect on colony formation at concentrations lower than IC_50_ in the MTT test. Moreover, its effect on the proliferation of MCF-7 cells has been shown. Obviously, this whole group of compounds has not been studied sufficiently, and the potential of pentanorlanostane triterpenes as antitumor agents may be significant.

## 3. Materials and Methods

### 3.1. General Methods

UV spectra were recorded using a Shimadzu UV-1601PC spectrometer (Shimadzu Corporation, Kyoto, Japan) in methanol. NMR spectra were recorded in CDCl_3_ and acetone-d_6_ on a Bruker DPX-300 (Bruker BioSpin GmbH, Rheinstetten, Germany), a Bruker Avance III-500 (Bruker BioSpin GmbH, Rheinstetten, Germany), and a Bruker Avance III-700 (Bruker BioSpin GmbH, Rheinstetten, Germany) spectrometer. The NMR spectra were calibrated using the residual solvent signals (7.26/77.16 for CDCl_3_ and 2.05/29.84 for acetone-d6, according to [35]). HRESIMS spectra were obtained using a Maxis Impact Mass Spectrometer (Bruker Daltonics GmbH, Rheinstetten, Germany).

Low-pressure liquid column chromatography was performed using silica gel (50/100 μm, Imid Ltd., Krasnodar, Russia) and Gel ODS-A (12 nm, S-75 μm, YMC Co., Ishikawa, Japan). Plates precoated with silica gel (5–17 μm, 4.5 cm × 6.0 cm, Imid Ltd., Krasnodar, Russia) and silica gel 60 RP-18 F254S (20 cm × 20 cm, Merck KGaA, Darmstadt, Germany) were used for thin-layer chromatography. Preparative HPLC was performed on a Shimadzu LC-20 chromatograph (Shimadzu USA Manufacturing, Canby, OR, USA) with a Shimadzu RID-20A refractometer (Shimadzu Corporation, Kyoto, Japan) using YMC-Pack Pro C18 (250 mm × 10 mm, YMC Co., Ishikawa, Japan), Synergi Fusion-RP 80 (250 mm × 10 mm, Phenomenex, Torrance, CA, USA), and Nautilus 110-5-C18 R (250 mm × 10 mm, Biochimmak ST, Moscow, Russia) columns.

### 3.2. Fungal Strain

The fungal strain KMM 4696 was isolated from the surface of the thallus of the brown alga *Sargassum pallidum* (Sea of Japan) and identified as *Asteromyces cruciatus* based on morphological (Figure S53) and molecular genetic features [9]. The fungal strain is stored in the Collection of Marine Microorganisms (KMM) of PIBOC FEB RAS (Vladivostok, Russia).

### 3.3. DNA Extraction and Amplification

Genomic DNA of strain KMM 4696 was isolated from mycelium grown on malt extract agar (MEA) at 25 °C for 7 days using the MagJET Plant Genomic DNA Kit (Thermo Fisher Scientific, Waltham, MA, USA), following the manufacturer’s protocol. PCR was performed using GoTaq Flexi DNA Polymerase (Promega, Madison, WI, USA). For amplification of the partial 28S rRNA (LSU) region, the standard primer pair LROR and LR5 was used [36]. The reaction profile was 95 °C for 300 s, 35 cycles of 95 °C for 30 s, 50 °C for 45 s, 72 °C for 120 s, and finally 72 °C for 300 s. The ITS region was amplified using the primer pair 1400-F (5′-CTGCCCTTTGTACACACCGCCCGTC-3′) [37] and D2CR (5′-CCTTGGTCCGTGTTTCAAGA-3′) [38]. The reaction profile was 95 °C for 300 s, followed by 35 cycles of 94 °C for 20 s, 55 °C for 20 s, 72 °C for 90 s, and finally 72 °C for 300 s. The partial TEF1 gene was amplified using the standard primer pair EF1-728F (5′-CATCGAGAAGTTCGAGAAGG-3′) and EF1-986R (5′-TACTTGAAGGAACCCTTACC-3′) [39]. The reaction profile was 95 °C for 300 s, 35 cycles of 95 °C for 30 s, 56 °C for 45 s, 72 °C for 90 s, and finally 72 °C for 210 s. The amplified 28S rRNA, ITS, and TEF1 genes were purified and sequenced as described previously [40]. The gene sequences were deposited in GenBank under the accession numbers are presented in Table 2.

### 3.4. Multi-Locus Phylogenetic Analysis

The regions of LSU, ITS and the partial *TEF1* gene sequences of the fungal strain KMM 4696 *Asteromyces cruciatus* and related species of the genera *Exserohilum*, *Gibbago*, *Stemphylium*, *Paradendryphiella*, *Asteromyces* and *Neostemphylium* belonging to the *Pleosporaceae* family, according to [12], were individual aligned by MEGA X software version 11.0.9 using Clustal W algorithm [41].

A search of LSU, ITS and *TEF1* sequences of ex-type and reference strains was performed in the GenBank database using the BLASTn algorithm (http://www.ncbi.nlm.nih.gov/BLAST, accessed on 30 November 2025). Multiple alignment of LSU, ITS and *TEF1* sequences of the strain KMM 4696 *Asteromyces cruciatus* and related species (listed in Table 2) were carried out using MEGA X software version 11.0.9 [41]. A bootstrap test based on 500 replicates was used to statistically estimate branch support. The NJ tree is rooted to *Neocamarosporium goegapense* CPC 23676^T^.

### 3.5. Cultivation and Extraction of Fungus

The strain KMM 4696 strain was cultivated on rice medium at 22 °C for three weeks in 98 Erlenmeyer flasks (500 mL), each containing 20 g of rice, 20 mg of yeast extract, 10 mg of KH_2_PO_4_, and 40 mL of natural seawater from the Marine Experimental Station of PIBOC FEB RAS, Troitsa (Trinity) Bay, Sea of Japan, and 2.5 mg/mL of KI.

At the end of the incubation period, the mycelia of the strain KMM 4696 fungus, together with the medium, were homogenized and extracted with EtOAc (2 × 8 L). The extract was then concentrated to dryness. The dry residue (14.9 g) was dissolved in a H_2_O—EtOH (4:1) system (200 mL) and consistently extracted with *n*-hexane (3 × 0.2 L) and EtOAc (3 × 0.2 L).

### 3.6. HPLC MS

UPLC MS analysis was performed using a Bruker Elute UPLC chromatograph (Bruker Daltonics, Bremen, Germany) connected to a Bruker Impact II Q-TOF mass spectrometer (Bruker Daltonics, Bremen, Germany). An InfinityLab Poroshell 120 SB-C18 column (2.1 × 150 mm, 2.7 μm, Agilent Technologies, Santa Clara, CA, USA) was used for chromatographic separation. A detailed description of chromatographic separation and mass spectrometric detection has been reported previously [42].

### 3.7. Isolation of Individual Compounds

The ethyl acetate part (after consistent extraction, see Section 3.5) was evaporated to dryness (8.5 g) and chromatographed on a silica gel column (4 × 30 cm), which was first eluted using a step gradient from 0% to 100% EtOAc in *n*-hexane. The fractions were collected and combined based on TLC (Si gel, toluene–isopropanol 6:1 and 9:4, *v*/*v*) data.

The fraction eluted with *n*-hexane (100%, 1895 mg) was separated on a SiO_2_ column (3 × 15 cm), eluted with a step gradient from 0% to 100% EtOAc in *n*-hexane, and then rechromatographed on a SiO_2_ column (2 × 10 cm) in *n*-hexane to yield **6** (165 mg).

The fraction of *n*-hexane-EtOAc (90:10, 111.1 mg) was separated on a SiO_2_ column (1.5 × 9 cm), which was eluted with a step gradient from 0% to 100% EtOAc in *n*-hexane to afford subfractions IV and V. Subfraction IV (5% EtOAc, 27.3 mg) was separated on a YMC ODS-A reverse-phase column (3 × 3 cm) with 100% MeOH and purified by reverse-phase HPLC on a YMC ODS-AM column (EtOH–H_2_O, 95:5) to yield **8** (7.5 mg). Subfraction V (10% EtOAc, 33 mg) was separated on a YMC ODS-A reverse-phase column (3 × 3 cm) with 100% MeOH and purified by reverse-phase HPLC on a YMC ODS-AM column (EtOH–H_2_O, 95:5) to yield **6** (16.3 mg).

The fraction eluted with *n*-hexane–EtOAc (80:20, 801.2 mg) was separated on a YMC ODS-A reverse-phase column (3 × 20 cm), which was eluted with 80% MeOH, and repeatedly rechromatographed on a SiO_2_ column (1.5 × 11 cm) (a step gradient from 0% to 100% EtOAc in *n*-hexane), on a YMC ODS-A reverse-phase column (1.5 × 12 cm) (100% MeOH), and by reverse-phase HPLC on a YMC ODS-AM column (EtOH–H_2_O, 92:8) to yield **1** (3.6 mg) and **2** (0.8 mg).

The fraction of *n*-hexane-EtOAc (60:40, 402 mg) was separated on a YMC ODS-A reverse-phase column (MeOH–H_2_O, 80:20) and then rechromatographed on a SiO_2_ column (2 × 20 cm) (a step gradient from 0% to 100% EtOAc in *n*-hexane) and a YMC ODS-A reverse-phase column (MeOH–H_2_O, 80:20) to yield **3** (0.22 mg), **10** (3.3 mg), and subfractions IX-3 and IX-5. Subfraction IX-3 (4.9 mg) was separated by reverse-phase HPLC on a Phenomenex Fusion column (MeOH–H_2_O, 50:50), and on a YMC Chiral column (MeOH–H_2_O, 50:50) to yield **5** (0.44 mg), **9** (3.3 mg), and **11** (0.72 mg). Subfraction IX-5 was separated by reverse-phase HPLC on a Nautilus 110-5-C18 R chiral column (MeCN–H_2_O, 40:60) to yield **4** (0.4 mg).

### 3.8. Spectral Data

Curvalarol C (**1**): amorphous solids; UV (MeOH) *λ*_max_ (log *ε*) 195 (3.82) nm (see Supplementary Figure S43); CD (*c* 7.0 × 10^−4^, MeOH), λ_max_ (∆ε) 197 (1.52), 225 (−0.40), 228 (−0.38) nm (see Supplementary Figure S44); ^1^H and ^13^C NMR data, see Table 1 and Supplementary Figures S5–S11; HRESIMS *m*/*z* 461.3616 [M + H]^+^ (calcd. for C_29_H_49_O_4_, 461.3625), 483.3445 [M + Na]^+^ (calcd. for C_29_H_48_O_4_Na, and 483.3445 (see Figure S46).

Curvalarol D (**2**): amorphous solids; for ^1^H and ^13^C NMR data, see Table 1 and Supplementary Figures S12–S16; HRESIMS *m*/*z* 453.2976 [M + Na]^+^ (calcd. for C_27_H_42_O_4_Na, 453.2975) (see Figure S47).

Acruciquinone D (**3**): amorphous solids; UV (MeOH) λmax (log ε) 466 (3.75), 293 (4.05), 272 (4.00) nm, 199 (4.16) nm (see Supplementary Figure S45); ^1^H and ^13^C NMR data, see Table 1 and Supplementary Figures S17–S22; HRESIMS *m*/*z* 296.0929 [M − H]^−^ (calcd. for C_17_H_14_NO_4_, 296.0928), 320.0891 [M + Na]^+^ (calcd. for C_17_H_15_NO_4_Na, 320.0893) (see Figure S48).

### 3.9. Bioassays

#### 3.9.1. Antimicrobial and Biofilm Formation Assay

The Gram-positive bacterial *Staphylococcus aureus* ATCC 21027, Gram-negative bacterial *Escherichia coli* VKPM (B-7935) and yeast-like fungal *Candida albicans* KMM 455 strains were fermented on solid medium Mueller Hinton broth with agar (16.0 g/L) in a Petri dish at 37 °C for 24 h.

The antimicrobial activities of the compounds were tested at concentrations 1–100 µM. The effect of the compounds on the growth of the test strains was estimated in accordance with [43]. The compounds were dissolved in DMSO and 10 µL of each was added in the wells of 96-well plates. The need concentrations were obtained via consistent two-fold dilutions. The microbial suspensions (1 × 10^6^ CFU/mL) were added in the wells (90 µL). The plates were incubated for 18 h at 37 °C. The optical density of the microbial suspension after 18 h was measured at 620 nm wavelength. The effect of compounds on the biofilm formation for 18 h was tested using MTT reagent (Sigma-Aldrich, St. Louis, MO, USA), in accordance with [44]. The biofilms were washed twice with PBS and incubated with MTT reagent (10 µM in PBS) for 4 h, after that the biofilms were lysed with 100% DMSO. The optical density of the obtained solution was measured at 570 nm wavelength. A MultiskanFS spectrophotometer (Thermo Scientific Inc., Beverly, MA, USA) was used in both assays. In both assays, gentamicin was used as a positive control at a concentration of 1 mg/mL and a 1% DMSO solution in PBS was used as a negative control. The results were calculated as percentages of the negative control.

#### 3.9.2. Cell Lines and Culture Conditions

The human breast cancer MCF-7 cells were purchased from American Type Culture Collection (Manassas, VA, USA). The normal human breast epithelial MCF-10A cell line was kindly provided by Dr. Irina Zhitnyak from N.N. Blokhin National Medical Research Center of Oncology, Moscow, Russia. The cells were cultured in DMEM with 10% of fetal bovine serum, and 1% of penicillin/streptomycin (BioloT, St. Petersburg, Russia). The cells were incubated at 37 °C and 5% CO_2_ (*v*/*v*).

#### 3.9.3. Cell Viability Assay

MTT assay was used for cell viability investigation. The MCF-7 cells were seeded in 96-well plates at density of 5 × 10^3^ cell/well and the experiments were started after 24 h.

All compounds were dissolved in DMSO such that the final concentration of DMSO in the cell culture did not exceed 1%. A total of 1% DMSO was used as a control. The compounds at concentrations up to 100 µM were added to the wells for 24 h or 48 h, and the viability of the cells was measured using an MTT (3-(4,5-dimethylthiazol-2-yl)-2,5-diphenyltetrazolium bromide) reagent, which was performed according to the manufacturer’s instructions (Sigma-Aldrich, Munich, Germany). The culture media was changed with fresh DMEM without fetal bovine serum and antibiotics and MTT reagent (5 mg/mL in PBS) was added for 4 h. After that, the media was removed, and the cells were lysed with 100% DMSO. Optical density of the obtained solutions was measured at 570 nm wavelength was measured using a MultiskanFS spectrophotometer (Thermo Scientific Inc., Beverly, MA, USA). The results are presented as percentages of the control data.

#### 3.9.4. Colony Formation Assay

The influence of the investigated compound on colony formation by MCF-7 cells was tested using a clonogenic assay [45]. The concentration of MCF-7 cells was 0.33 × 10^3^/mL. The cells were incubated for 10 d, fixed with methanol (25 min), stained with 0.5% crystal violet solution (25 min), and washed with PBS. The colonies were counted using a BIO-PRINT-Cx4 Edge-Fixed Pad-Container (Vilber, Collegien, France) using the Bio-Vision Software user and service manual-v18.01 (Vilber, Collegien, France). The results are presented as colony units per well.

#### 3.9.5. EdU Incorporation Assay

The MCF-7 cells were seeded in a 96-well plate at density of 5 × 10^3^ cells/well and treated with the compound at concentrations of 1 µM and 10 µM for 24 h and 48 h. Before 2 h of incubation, a dH_2_O solution of 5-ethynyl-2′-deoxyuridine (EdU) (Lumiprobe, Moscow, Russia) at a concentration of 10 µM was added to each well for 2 h and then stained according to the manufacturer’s instructions. The cell monolayer was rinsed with phosphate-buffered saline (PBS) three times and permeabilized with 0.2% Triton X-100 (Helicon, Moscow, Russia) in PBS for 30 min at room temperature. Then, the cells were stained through a click reaction with ascorbic acid at 10 mM (Lumiprobe, Moscow, Russia), Cu(II)-BTTAA complex at 2 mM (Lumiprobe, Moscow, Russia), and sulfo-Cyanine5 Azide at 8 µM (Lumiprobe, Moscow, Russia) in PBS for 30 min at room temperature in the dark. The cells were then washed twice with PBS. The cells were stained with DAPI and visualized under a fluorescence microscope. Images at the same position at λex = 630 and λem = 670 nm (sulfo-Cyanine5) and λex = 358 and λem = 461 nm (DAPI) were obtained using an MIB-2-FL fluorescent microscope (Lomo Microsystems, St. Peterburg, Russia) for at least four positions in each well. Nearly 15 images were obtained for each cell incubation variant. The number of cells and EdU-positive cells in each position was calculated using stardist [46] for the initial segmentation of nuclei and CellProfiler [47] for nuclei counting. The percentage of EdU-positive cells for each variant was calculated. Image processing is detailed in [34].

#### 3.9.6. Statistical Data Evaluation

All bioassay data were obtained in three independent replicates, and calculated values are expressed as the mean ± standard error mean (SEM) using SigmaPlot 14.0 (Systat Software Inc., San Jose, CA, USA). Student’s *t*-test was performed using SigmaPlot 14.0 (Systat Software Inc., San Jose, CA, USA) to determine statistical significance. Differences were considered statistically significant at *p* < 0.05.

## 4. Conclusions

The KMM 4696 strain was identified as *Asteromyces cruciatus* based on the 28S rRNA, ITS, and TEF1 phylogenetic markers. Previously undescribed pentanorlanostane triterpenoids curvalarols C (**1**) and D (**2**), and a unique nitrogen-containing 6/6/5-anthraquinone derivative acruciquinone D (**3**), together with eight known metabolites, were isolated from the EtOAc extract of a fungal culture grown on KI-containing medium. Curvalarol C (**1**) showed selective activity against cancer MCF-7 cells and inhibited colony formation with an IC_50_ of 4.7 µM.

## Figures and Tables

**Table 1:** ^1^H and ^13^C NMR spectroscopic data (δ in ppm, 700.13/125.75 MHz, acetone-d_6_) for **1**–**3**.

Position	1		2		3	
	δ_C_, mult	δ_H_ (*J* in Hz)	δ_C_, mult	δ_H_ (*J* in Hz)	δ_C_, mult	δ_H_ (*J* in Hz)
1	36.6, CH_2_	1.71, m 1.22, m	36.9, CH_2_	1.99, m 1.64, m	120.4, C	
2	28.6, CH_2_	1.62, m	34.9, CH_2_	2.55, m 2.38, m	161.0, CH	8.40, d (14.5)
3	78.5, CH	3.14, t (7.9)	216.2, C		133.7, CH	6.99, d (3.7)
4	39.6, C		47.7, C		119.2, CH	6.78, d (3.8)
5	51.4, CH	1.05, m	52.0, CH	1.63, m	120.5, CH	7.43, s
6	26.9, CH_2_	2.04 (overlapped)	20.1, CH_2_	1.62, m 1.67, m	147.0, C	
7	30.3, CH_2_	1.29 (overlapped)	26.8, CH_2_	2.10, m	123.5, CH	6.94, s
8	134.3, C		135.9, C		163.1, C	
9	137.4, C		135.6, C		184.6, C	
10	37.7, C		37.7, C		181.2, C	
11	34.7, CH_2_	2.54, ddt (18.4, 8.5, 2.5) 1.83, m	34.5, CH_2_	2.32, m	136.0, C	
12	71.7, CH	4.08, m	71.7, CH	4.10, brs	115.7, C	
13	52.8, C		52.8, C		124.2, C	
14	49.7, C		49.9, C		137.8, C	
15	31.8, CH_2_	1.19, brs	32.0, CH_2_	1.20, m 1.72, m	21.8, CH_3_	2.39, s
16	26.4, CH_2_	1.87, m 1.66, m	26.3, CH_2_	1.88, m 1.72, m	53.9, CH_3_	3.95, s
17	49.7, CH	2.10, dt (9.5,7.8)	49.9, CH	2.14, m	61.3, CH_3_	3.95, s
18	10.2, CH_3_	0.72, s	10.4, CH_3_	0.75, s		
19	19.6, CH_3_	1.02, s	19.0, CH_3_	1.14, s		
20	42.4, CH	2.65, quintet (7.1)	42.3, CH	2.66, t (7.0)		
21	19.6, CH_3_	1.29, d (7.0)	19.5, CH_3_	1.28, d (6.8)		
22	177.9, C		177.9, C			
28	28.5, CH_3_	0.98, brs	26.7, CH_3_	1.05, s		
29	16.1, CH_3_	0.80, s	21.5, CH_3_	1.03, s		
30	24.3, CH_3_	0.93, brs	24.4, CH_3_	0.96, s		
1′	64.0, CH_2_	4.01, t (6.6)	60.1, CH_2_	4.06, quintet (7.0)		
2′	31.5, CH_2_	1.60, brs	14.6, CH_3_	1.20, m		
3′	19.9, CH_2_	1.4, sextet				
4′	14.0, CH_3_	0.92, t (7.4)				
8-OH						12.82, s

**Table 2:** The strains of the species included in the multi-locus phylogenetic analysis and GenBank accession numbers.

Species	Strain Number	GenBank Accession Number		
		ITS	LSU	*TEF1*
*Asteromyces cruciatus*	CBS 171.63^T^	MH858254	MH869856	ON542234
*Asteromyces cruciatus*	KMM 4696	PP825141	PP825359	PP845345
*Exserohilum monoceras*	CBS 239.77	LT837474	LT883405	
*Exserohilum rostratum*	CBS 128061	KT265240	MH877986	
*Exserohilum turcicum*	CBS 387.58	MH857820	LT883412	
*Gibbago trianthemae*	NFCCI 1886	HM448998	MH870931	
*Neocamarosporium goegapense*	CPC 23676^T^	KJ869163	KJ869220	
*Neostemphylium polymorphum*	FMR 17886^T^	OU195609	OU195892	ON368192
*Paradendriphyella arinariae*	CBS 181.58^T^	MH857747	KC793338	
*Paradendriphyella salina*	CBS 302.84^T^	MH873443	KC584325	KC584709
*Stemphylium botryosum*	CBS 714.68^T^	MH859208	MH870931	KC584729
*Stemphylium lycopersici*	CNU 070067	JF417683		JX213347
*Stemphylium vesicarium*	CBS 191.86	MH861935	JX681120	KC584731

^T^—ex-type strain

## Data Availability

The original contributions presented in this study are included in the article/Supplementary Material. Further inquiries can be directed to the corresponding authors.

## Supplementary Materials

The following supporting information can be downloaded at: https://www.mdpi.com/article/10.3390/molecules30244772/s1, Figures S1–S38: NMR spectra of **1**–**11**, Figures S39–S41: UV and CD spectra of **1** and **3**, Figures S42–S52: HRESIMS spectra of **1**–**11**, Figure S53: Morphological characters of *Asteromyces cruciatus* KMM 4696, Figure S54: The phylogenetic tree of *Asteromyces cruciatus* KMM 4696; Table S1: the list of pentanorlanostane-type of natural compounds, Table S2: the HPLC MS data for **1** and **2**.
