# Supplementary material for: Cytotoxic Curvalarol C and Other Compounds from Marine Fungus Asteromyces cruciatus KMM 4696
Source: Molecules. 2025 Dec 14;30(24):4772. doi: 10.3390/molecules30244772 (PMC12736370; doi:10.3390/molecules30244772)
Supplement: Supplementary file 1 [file molecules-30-04772-s001.zip › molecules-4006883-supplementary.pdf]

Article

Cytotoxic Curvalarol C and Other Compounds from Marine Fungus *Asteromyces cruciatus* KMM 4696

Liliana E. Nesterenko [1], Ekaterina A. Yurchenko [1], Olesya I. Zhuravleva [1], Galina K. Oleinikova [1], Natalya N. Kirichuk [1], Roman S. Popov [1], Viktoria E. Chausova [1], Konstantin A. Drozdov [1], Ekaterina A. Chingizova [1], Marina P. Isaeva [1] and Anton N. Yurchenko [1,*]

[1] G.B. Elyakov Pacific Institute of Bioorganic Chemistry, Far Eastern Branch of the Russian Academy of Sciences, 159 Prospect 100-letiya Vladivostoka, Vladivostok 690022, Russian Federation

* Correspondence: yurchenkoan@piboc.dvo.ru

Abstract

The study of the influence of NaBr, KI, and NaF additives on the specialized metabolites production of obligate marine fungus *Asteromyces cruciatus* KMM 4696 was the aim of the present work. *Asteromyces cruciatus* KMM 4696 strain was identified based on 28S rRNA, ITS, and TEF1 molecular genetic markers. The addition of NaBr, KI, and NaF to the fungal culture significantly changed the UHPLC MS profiles of the fungal extracts. Chromatographic separation of the fungal extract fermented with KI resulted in the isolation of undescribed pentanorlanostanes curvalarols C (**1**) and D (**2**), and an undescribed 6/6/5 anthraquinone acruciquinone D (**3**), together with eight known metabolites. The structures of the isolated compounds were established based on the 1D and 2D NMR and MS data. The cytotoxic activity of curvalarol C (**1**) was assayed in MCF-10A and MCF-7 cells. Curvalarol C showed selective activity against cancer MCF-7 cells and inhibited colony formation with an IC_{50} of 4.7 µM.

Keywords: *Asteromyces cruciatus*; cultivation conditions; metabolite profile; phylogeny; anthraquinones; bioactivity

Contents

Figure S1. ^{1}H NMR spectrum (700 MHz, acetone-d6) of **1** 4

Figure S2. ^{13}C NMR spectrum (700 MHz, acetone-d6) of **1** 6

Figure S3. DEPT NMR spectrum (700 MHz, acetone-d6) of **1** 7

Figure S4. HSQC NMR spectrum (700 MHz, acetone-d6) of **1** 8

Figure S5. HMBC NMR spectrum (700 MHz, acetone-d6) of **1** 9

Figure S6. COSY NMR spectrum (700 MHz, acetone-d6) of **1** 10

Figure S7. ROESY NMR spectrum (700 MHz, acetone-d6) of **1** 11

Figure S8. ^{1}H NMR spectrum (700 MHz, acetone-d6) of **2** 12

Figure S9. ^{13}C NMR spectrum (700 MHz, acetone-d6) of **2** 13

Figure S10. HSQC NMR spectrum (700 MHz, acetone-d6) of **2** 14

Figure S11. HMBC NMR spectrum (700 MHz, acetone-d6) of **2** 15

Figure S12. COSY NMR spectrum (700 MHz, acetone-d6) of **2** 16

Figure S13. ^{1}H NMR spectrum (500 MHz, acetone-d6) of **3** 17

Figure S14. ^{13}C NMR spectrum (500 MHz, acetone-d6) of **3** 18

Figure S15. DEPT NMR spectrum (500 MHz, acetone-d6) of **3** 19

Figure S16. HSQC NMR spectrum (700 MHz, acetone-d6) of **3** 20

Figure S17. HMBC NMR spectrum (700 MHz, acetone-d6) of **3** 21

Figure S18. COSY NMR spectrum (700 MHz, acetone-d6) of **3** 22

Figure S19. ^{1}H NMR spectrum (500 MHz, acetone-d6) of **4** 23

Figure S20. ^{13}C NMR spectrum (500 MHz, acetone-d6) of **4** 25

Figure S21. DEPT NMR spectrum (500 MHz, acetone-d6) of **4** 26

Figure S22. ^{1}H NMR spectrum (500 MHz, acetone-d6) of **5** 27

Figure S23. ^{13}C NMR spectrum (500 MHz, acetone-d6) of **5** 29

Figure S24. DEPT NMR spectrum (500 MHz, acetone-d6) of **5** 30

Figure S25. ^{1}H NMR spectrum (500 MHz, $CDCl_3$) of **6** 31

Figure S26. ^{13}C NMR spectrum (500 MHz, $CDCl_3$) of **6** 32

Figure S27. ^{1}H NMR spectrum (500 MHz, acetone-d6) of **7** 33

Figure S28. ^{13}C NMR spectrum (500 MHz, acetone-d6) of **7** 34

Figure S29. DEPT NMR spectrum (500 MHz, acetone-d6) of **7** 35

Figure S30. ^{1}H NMR spectrum (500 MHz, acetone-d6) of **8** 36

Figure S31. ^{13}C NMR spectrum (500 MHz, acetone-d6) of **8** 37

Figure S32. DEPT NMR spectrum (500 MHz, acetone-d6) of **8** 38

Figure S33. ^{1}H NMR spectrum (500 MHz, CD_3OD) of **10** 39

Figure S34. ^{13}C NMR spectrum (500 MHz, CD_3OD) of **10** 40

Figure S35. DEPT NMR spectrum (500 MHz, CD_3OD) of **10** 41

Figure S36. ^{1}H NMR spectrum (500 MHz, acetone-d6) of **11** 42

Figure S37. ^{13}C NMR spectrum (500 MHz, acetone-d6) of **11**........ 43

Figure S38. DEPT NMR spectrum (500 MHz, acetone-d6) of **11**........ 44

Figure S39. UV spectrum of **1** 46

Figure S40. ECD spectrum of **1**........ 46

Figure S41. UV spectrum of 3 47

Figure S42. (+) ESI MS of **1** 48

Figure S43. (+) ESI MS of **2** 48

Figure S44. (-) ESI MS of **3**........ 49

Figure S45. (-) ESI MS of **5**........ 50

Figure S46. (-) ESI MS of **6**........ 51

Figure S47. (-) ESI MS of **7**........ 52

Figure S48. (-) ESI MS of **8**........ 53

Figure S49. (-) ESI MS of **9** 54

Figure S50. (-) ESI MS of **10**........ 55

Figure S51. (-) ESI MS of **11**........ 56

Figure S52. (-) ESI MS of **4**........ 57

Table S1. The pentanorlanostane type compounds........ 58

Table S2. The HPLC MS data of compounds 1 and 2 62

Figure S53. Morphological characters of *Asteromyces cruciatus* KMM 4696. 63

Figure S54. Sequence variation of concatenated ITS-LSU-*tef1* between KMM 4696 and ex-types of the genera *Asteromyces, Stemphylium* and *Paradendryphiella*. ITS, LSU and *tef1* are shown in green, yellow and blue, respectively. Identical nucleotides are shown as asterisk........ 63

Figure S1. ^{1}H NMR spectrum (700 MHz, acetone-d6) of **1**

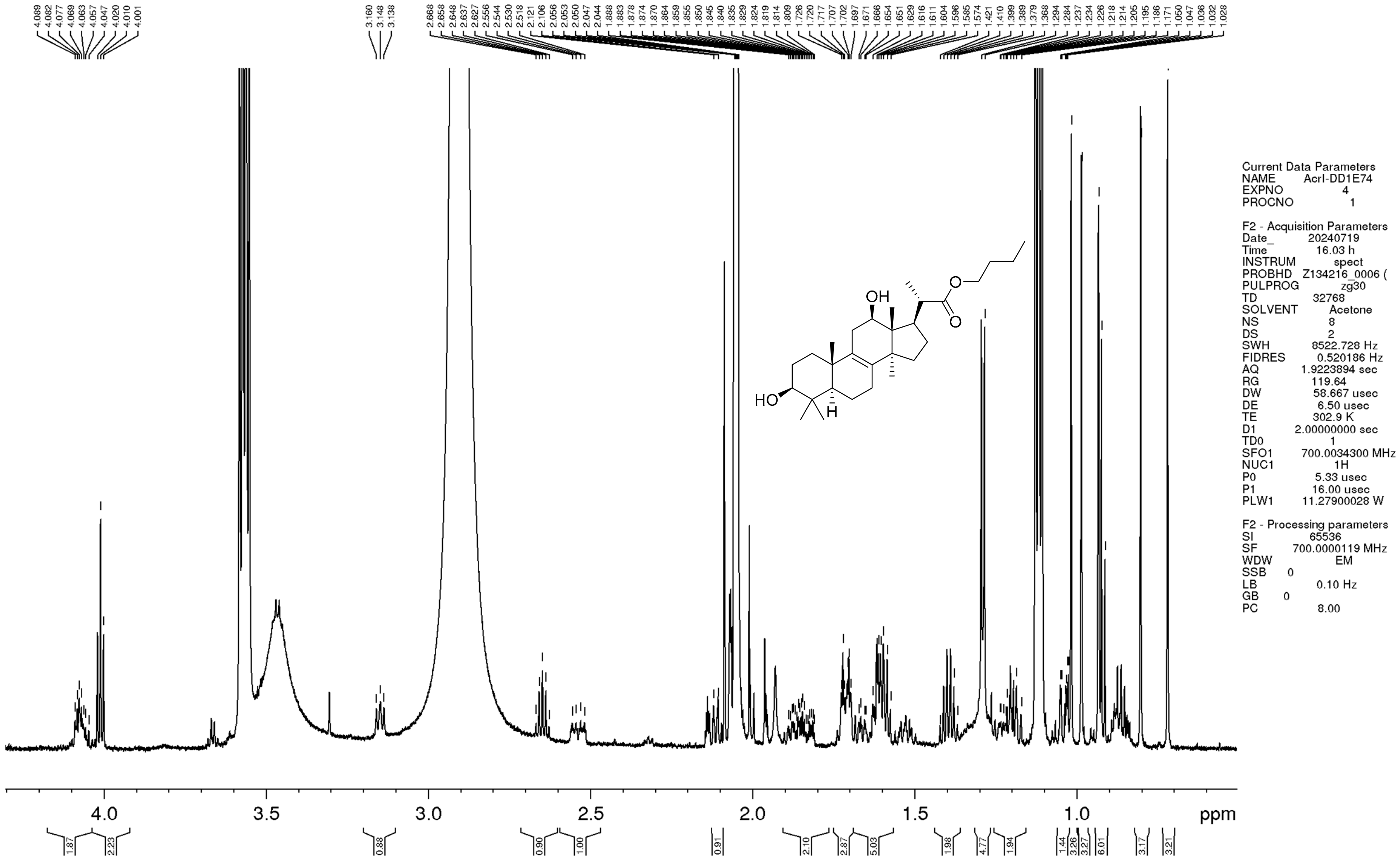

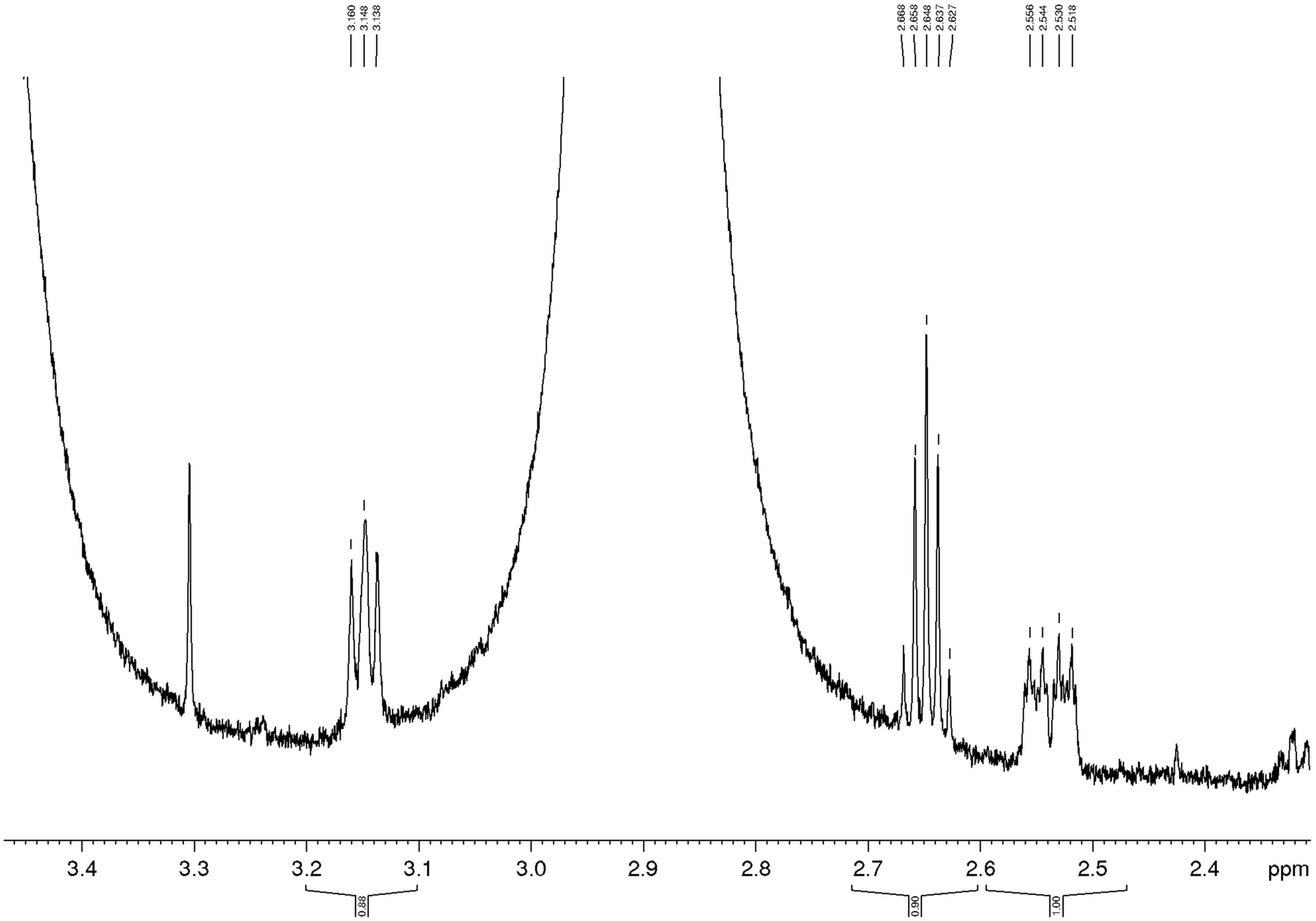

3.160
3.148
3.138
2.668
2.658
2.648
2.637
2.627
2.556
2.544
2.530
2.518
3.4
3.3
3.2
3.1
3.0
2.9
2.8
2.7
2.6
2.5
2.4
ppm
0.88
0.90
1.00

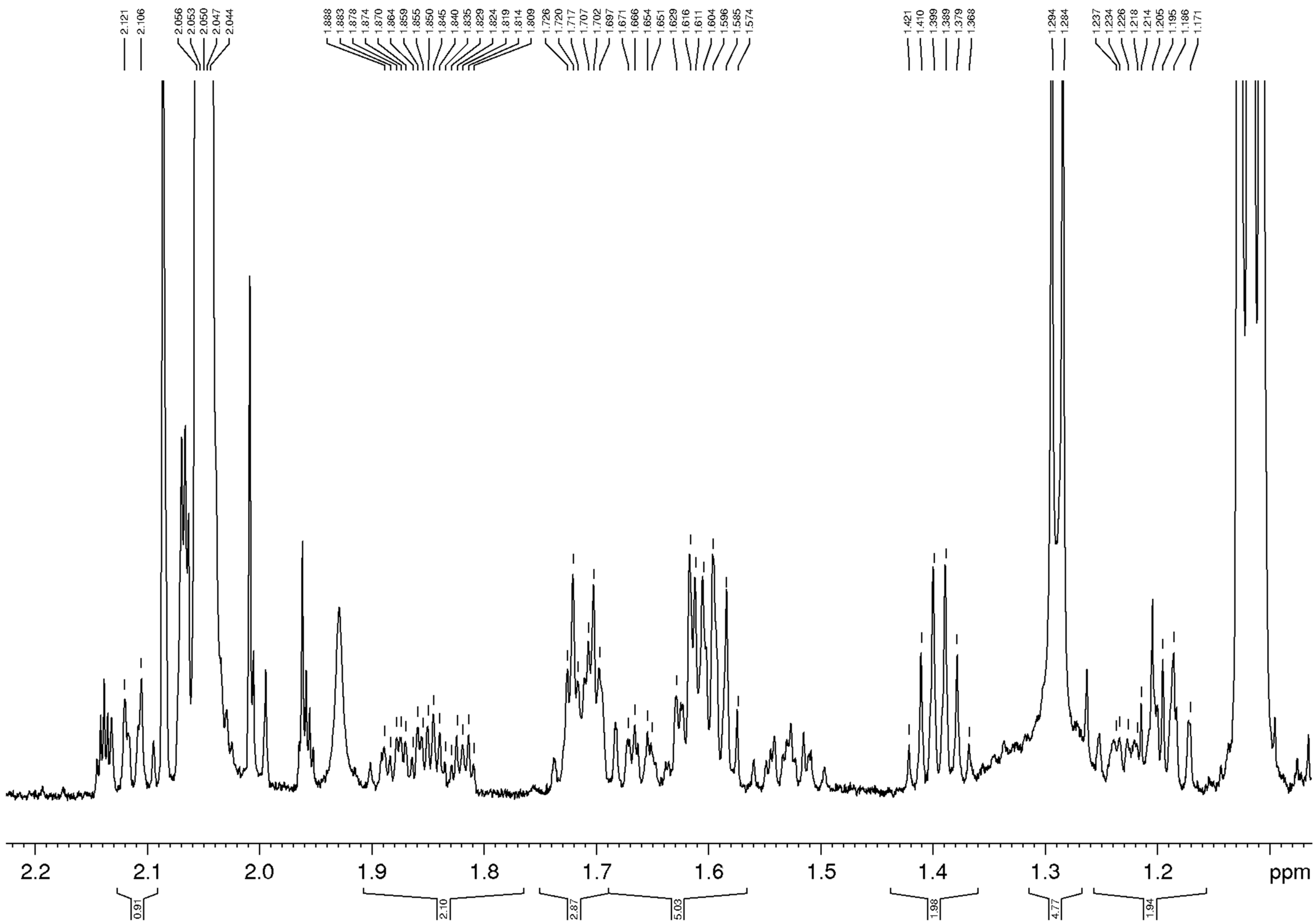

Figure S2. ^{13}C NMR spectrum (700 MHz, acetone-d6) of **1**

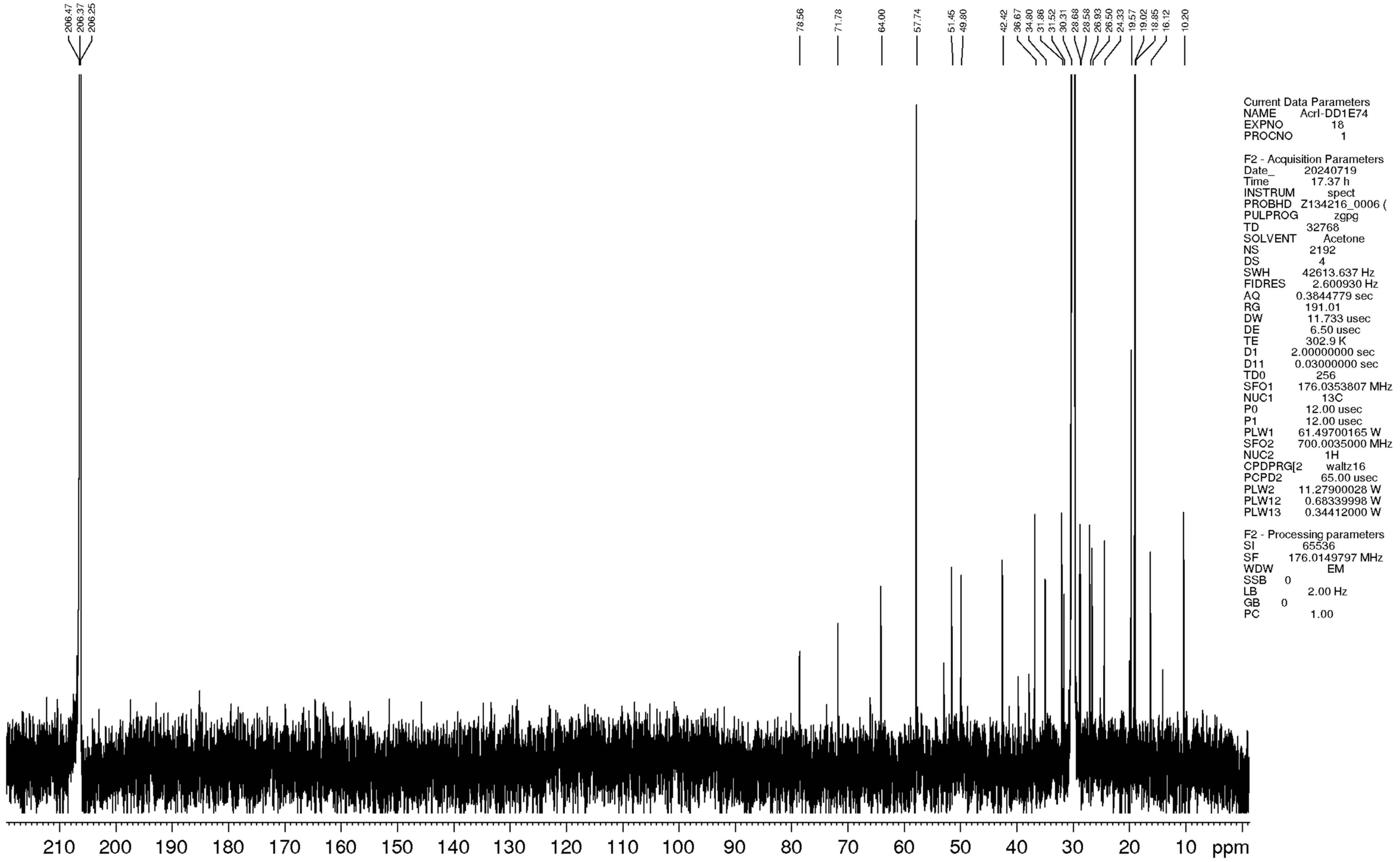

Figure S3. DEPT NMR spectrum (700 MHz, acetone-d6) of **1**

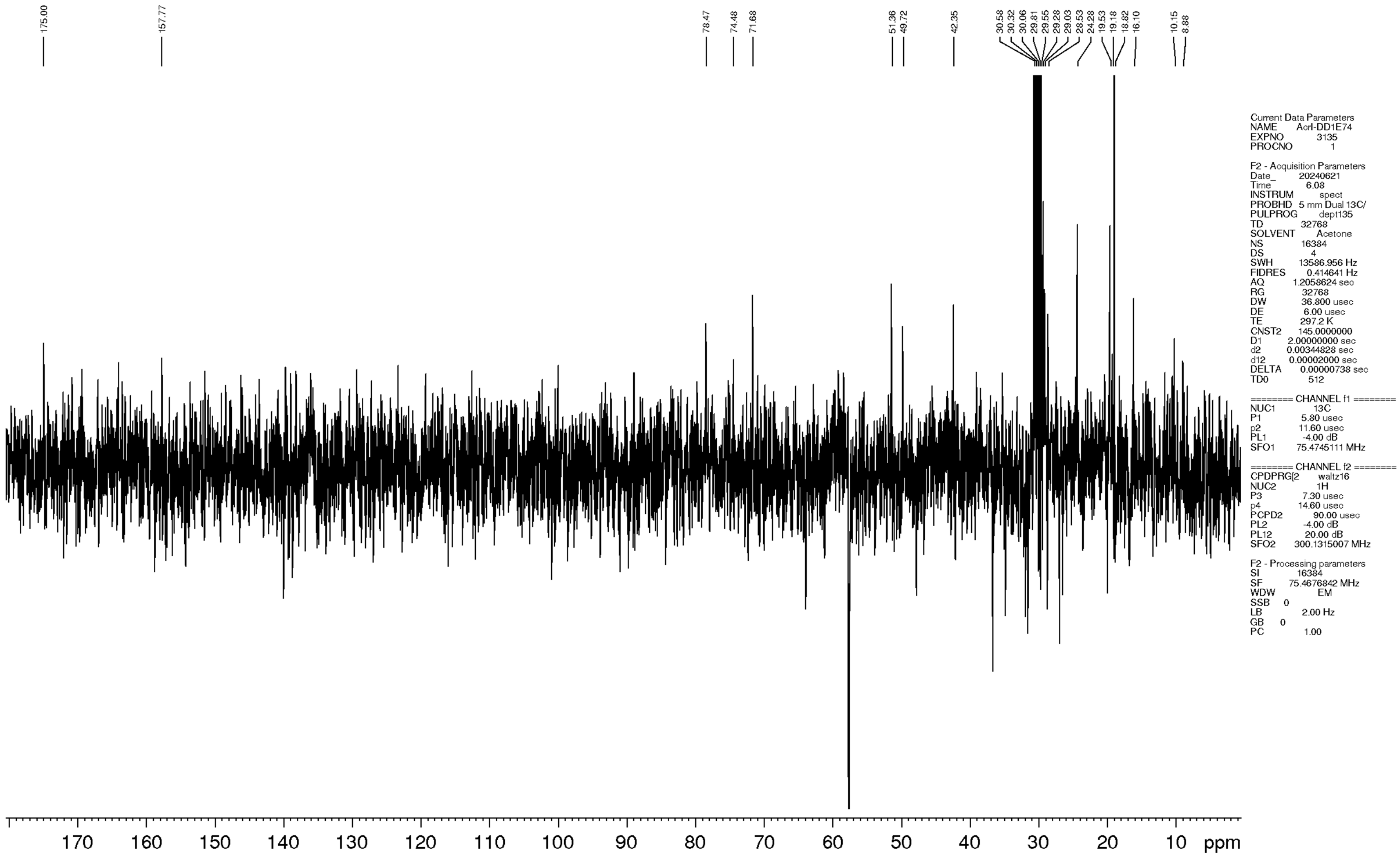

Figure S4. HSQC NMR spectrum (700 MHz, acetone-d6) of **1**

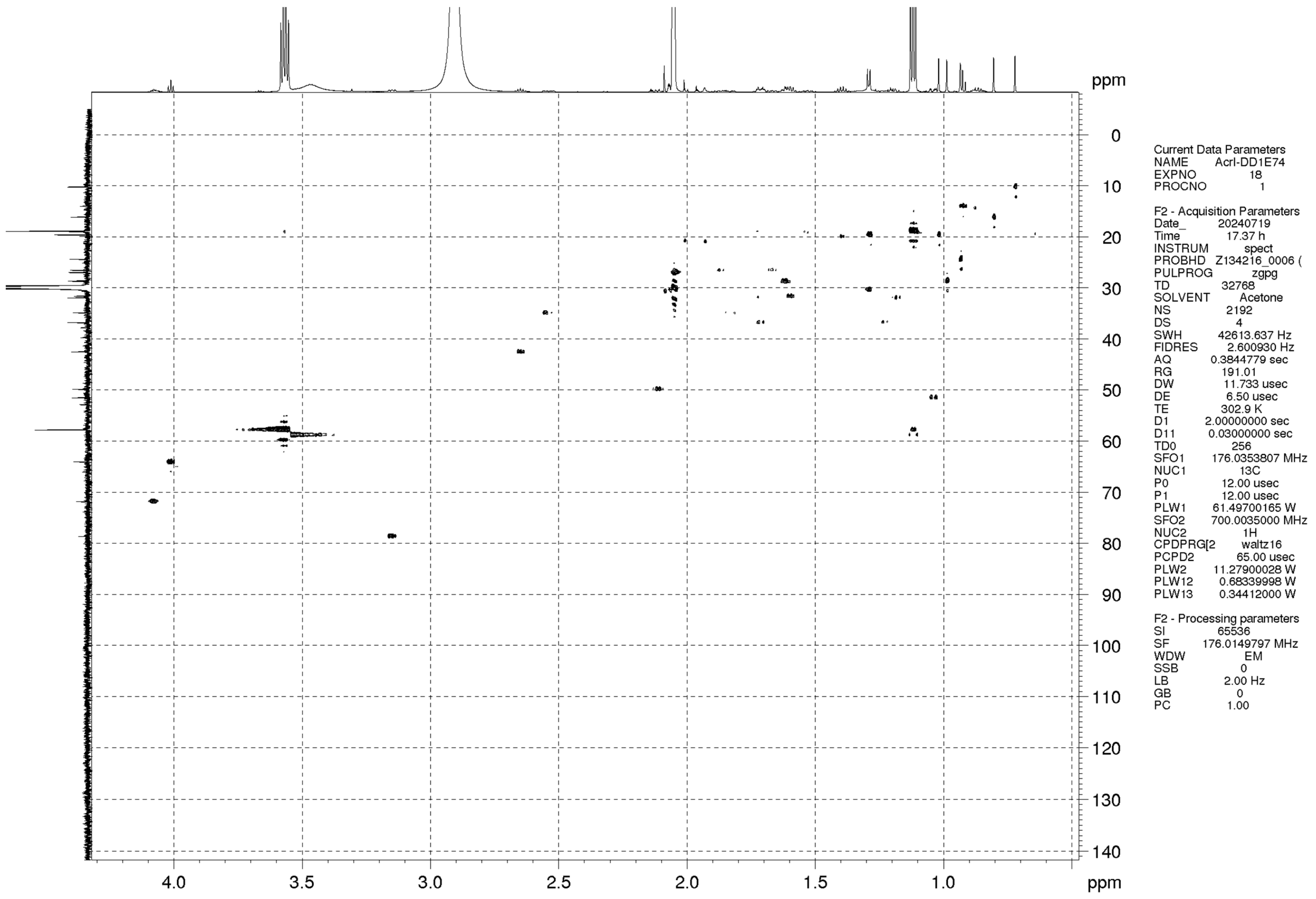

Figure S5. HMBC NMR spectrum (700 MHz, acetone-d6) of **1**

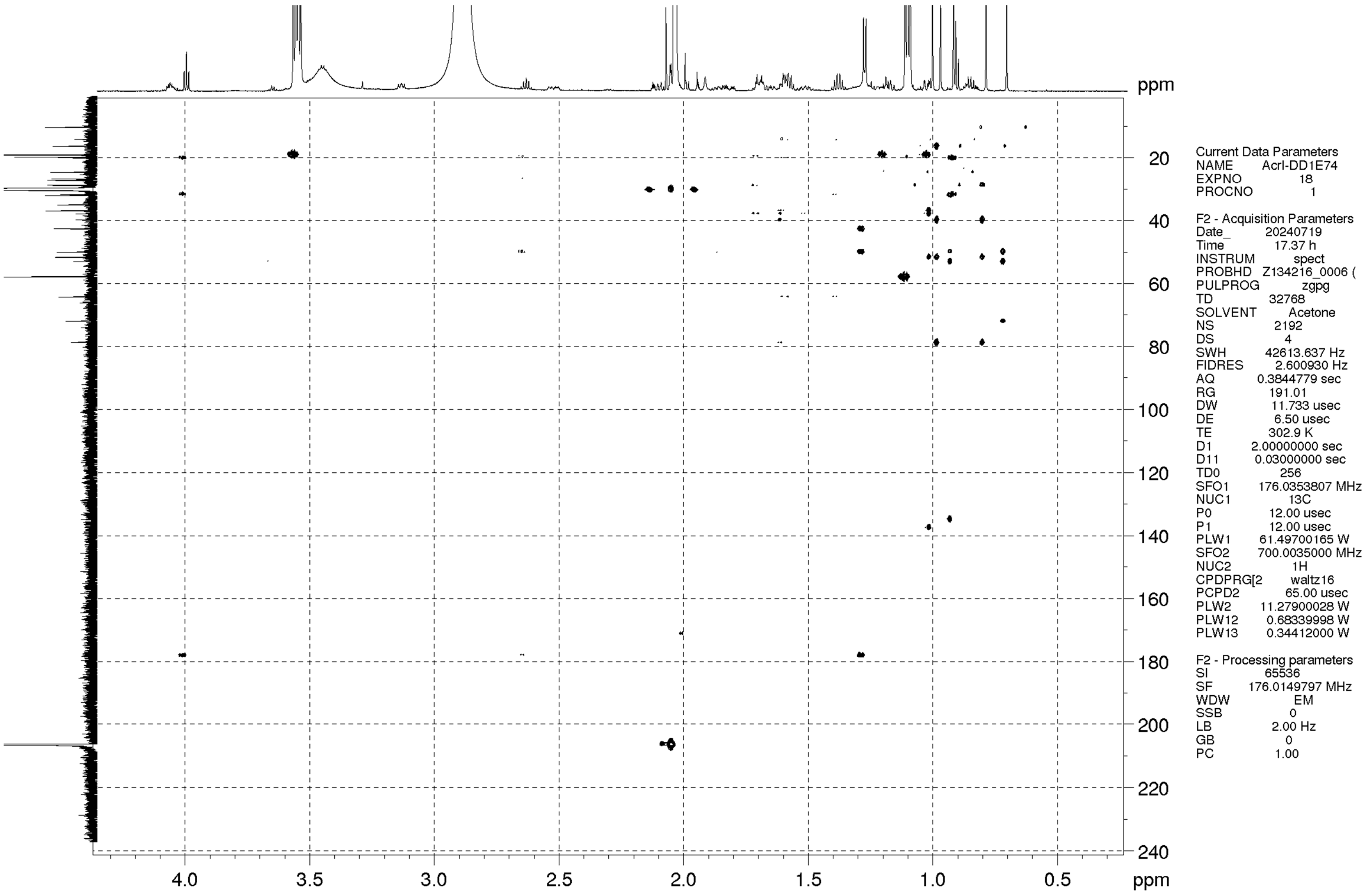

Figure S6. COSY NMR spectrum (700 MHz, acetone-d6) of **1**

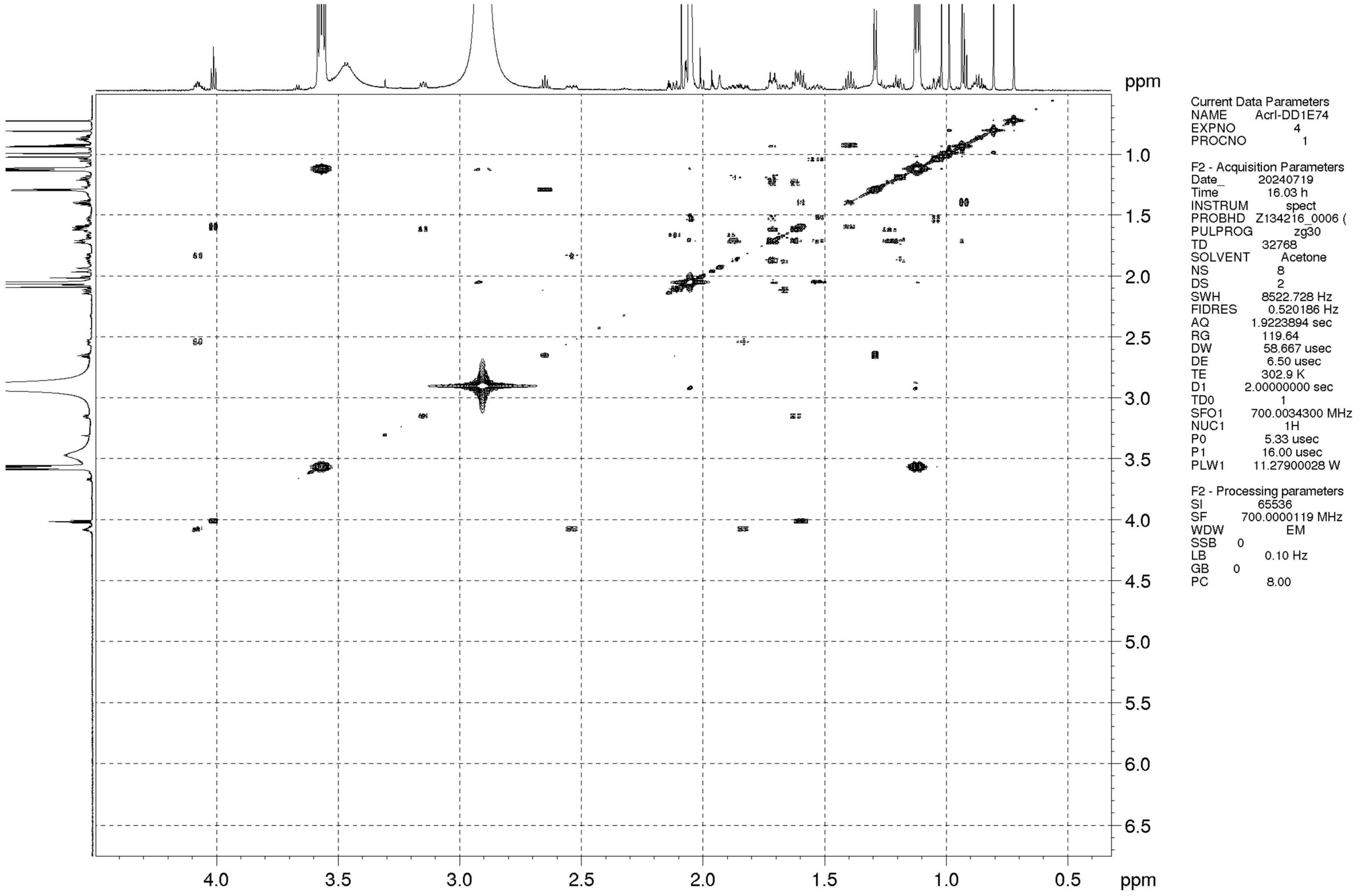

Figure S7. ROESY NMR spectrum (700 MHz, acetone-d6) of **1**

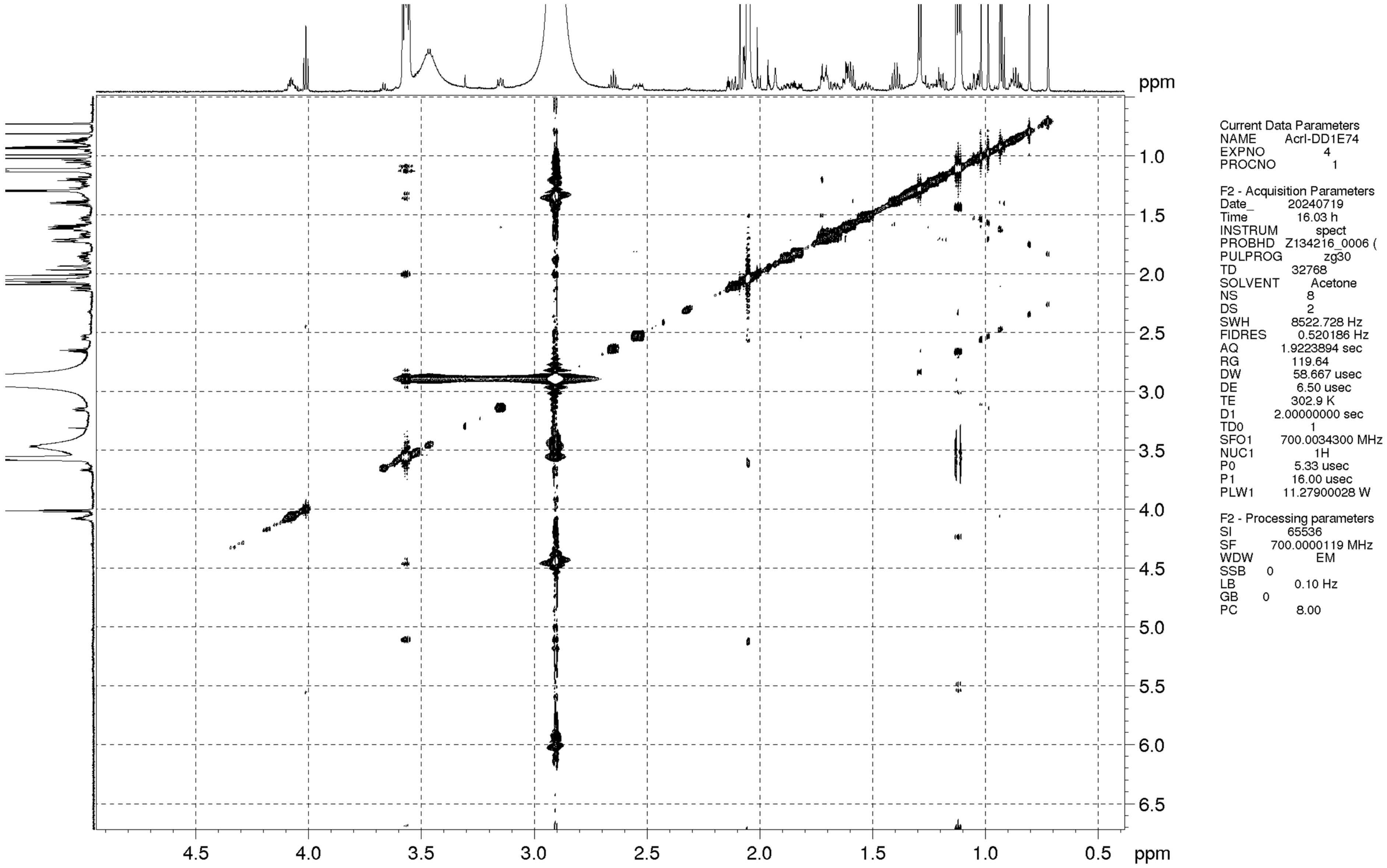

Figure S8. ^{1}H NMR spectrum (700 MHz, acetone-d6) of **2**

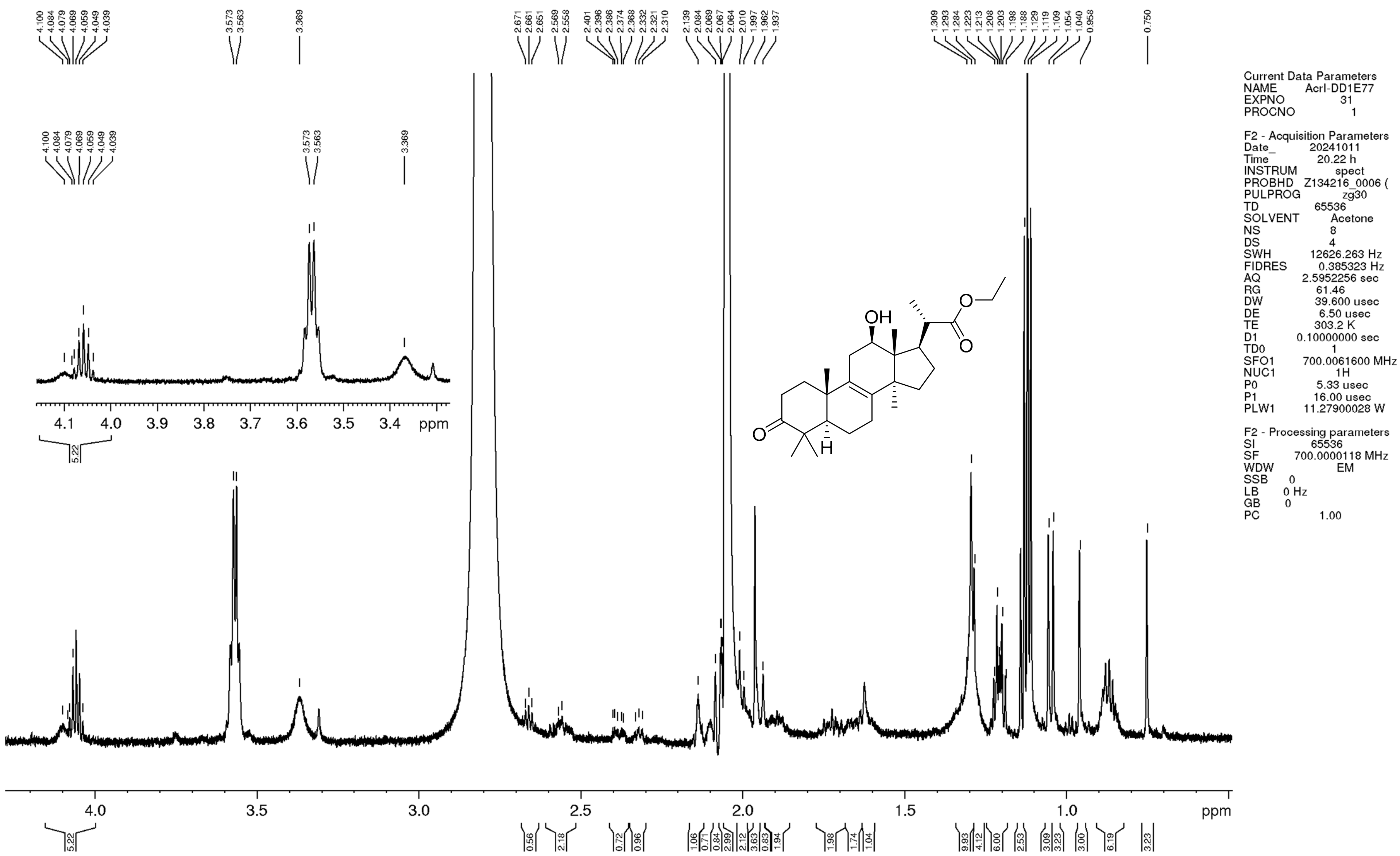

Figure S9. ^{13}C NMR spectrum (700 MHz, acetone-d6) of **2**

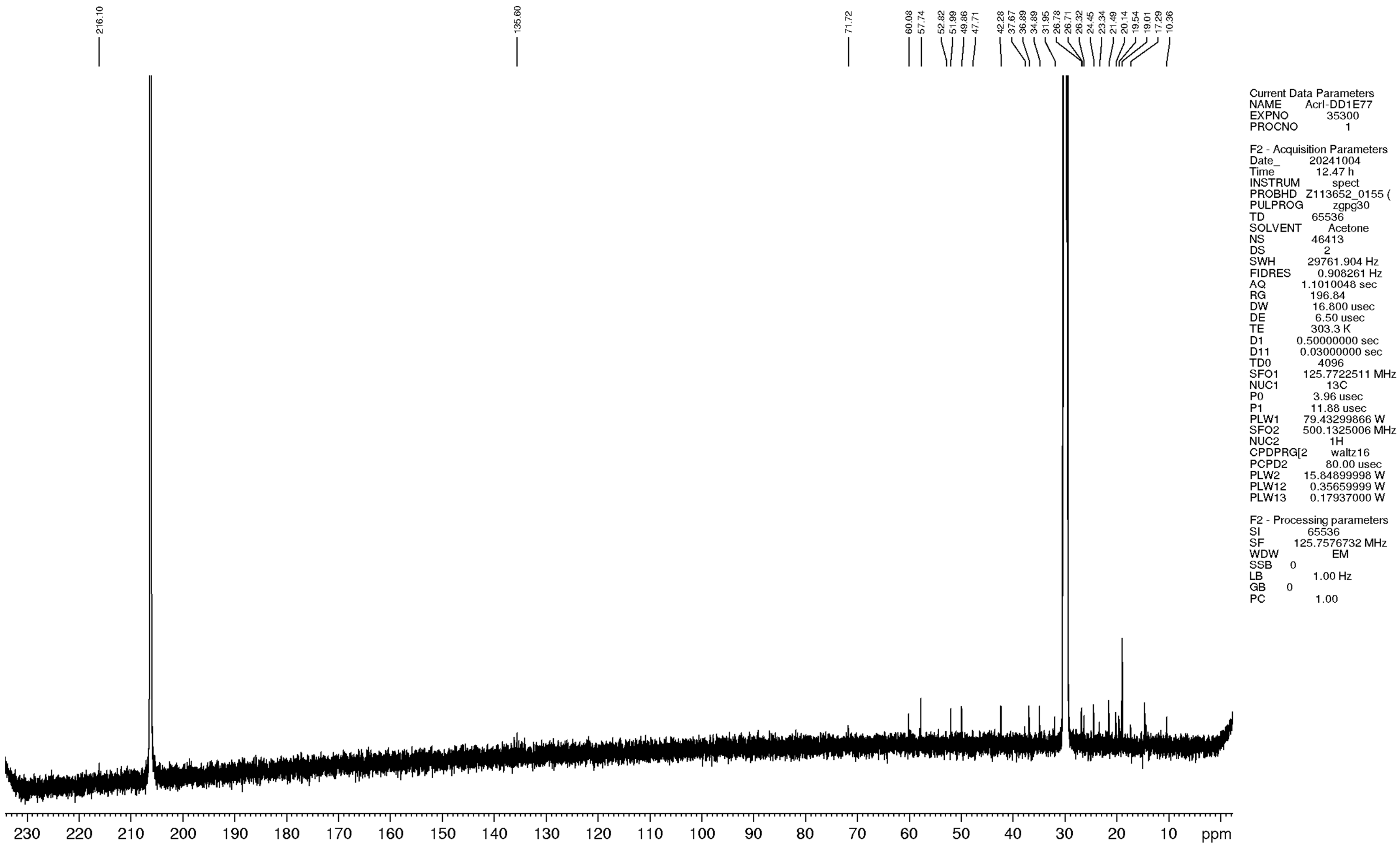

Figure S10. HSQC NMR spectrum (700 MHz, acetone-d6) of **2**

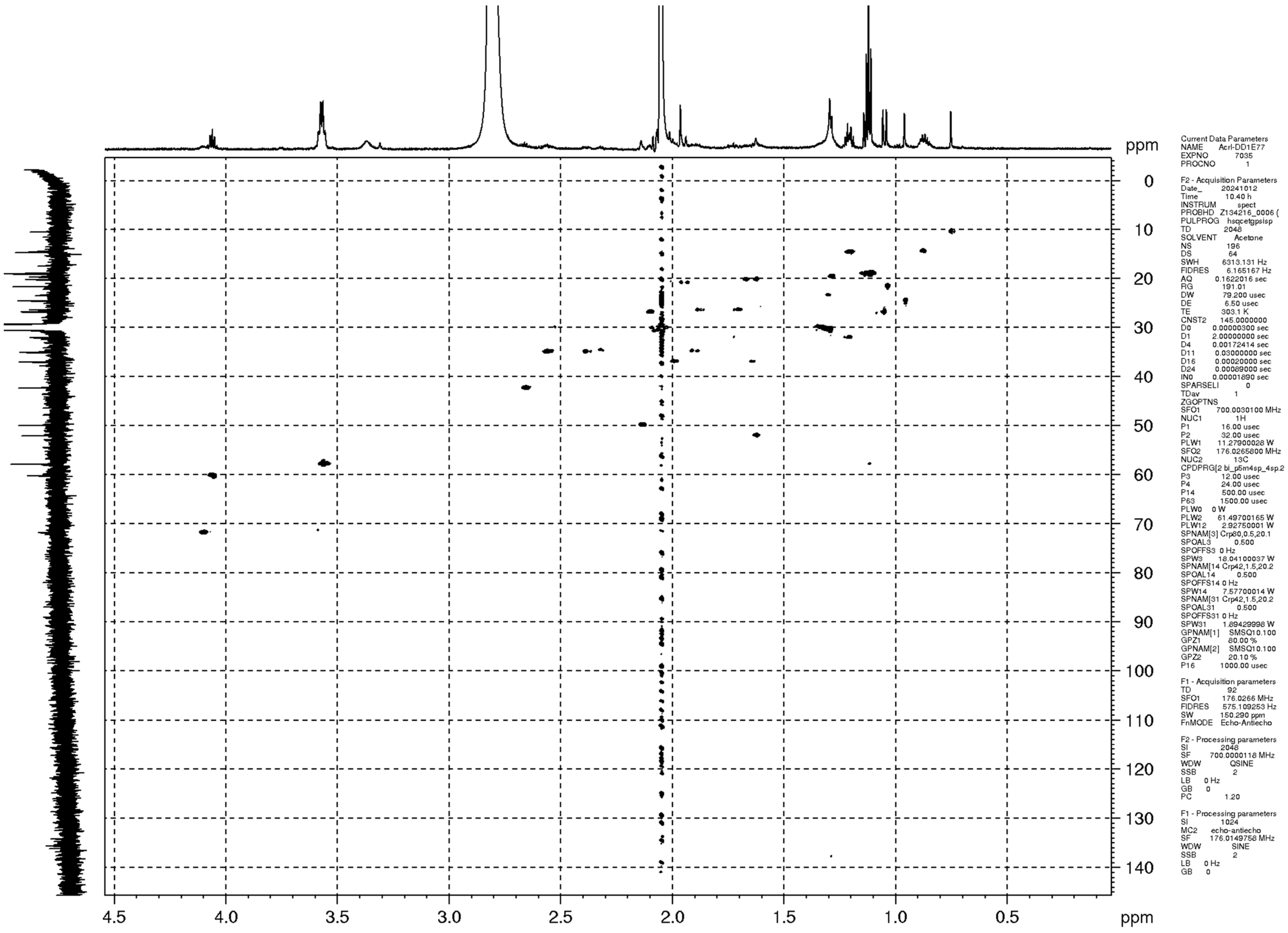

Figure S11. HMBC NMR spectrum (700 MHz, acetone-d6) of **2**

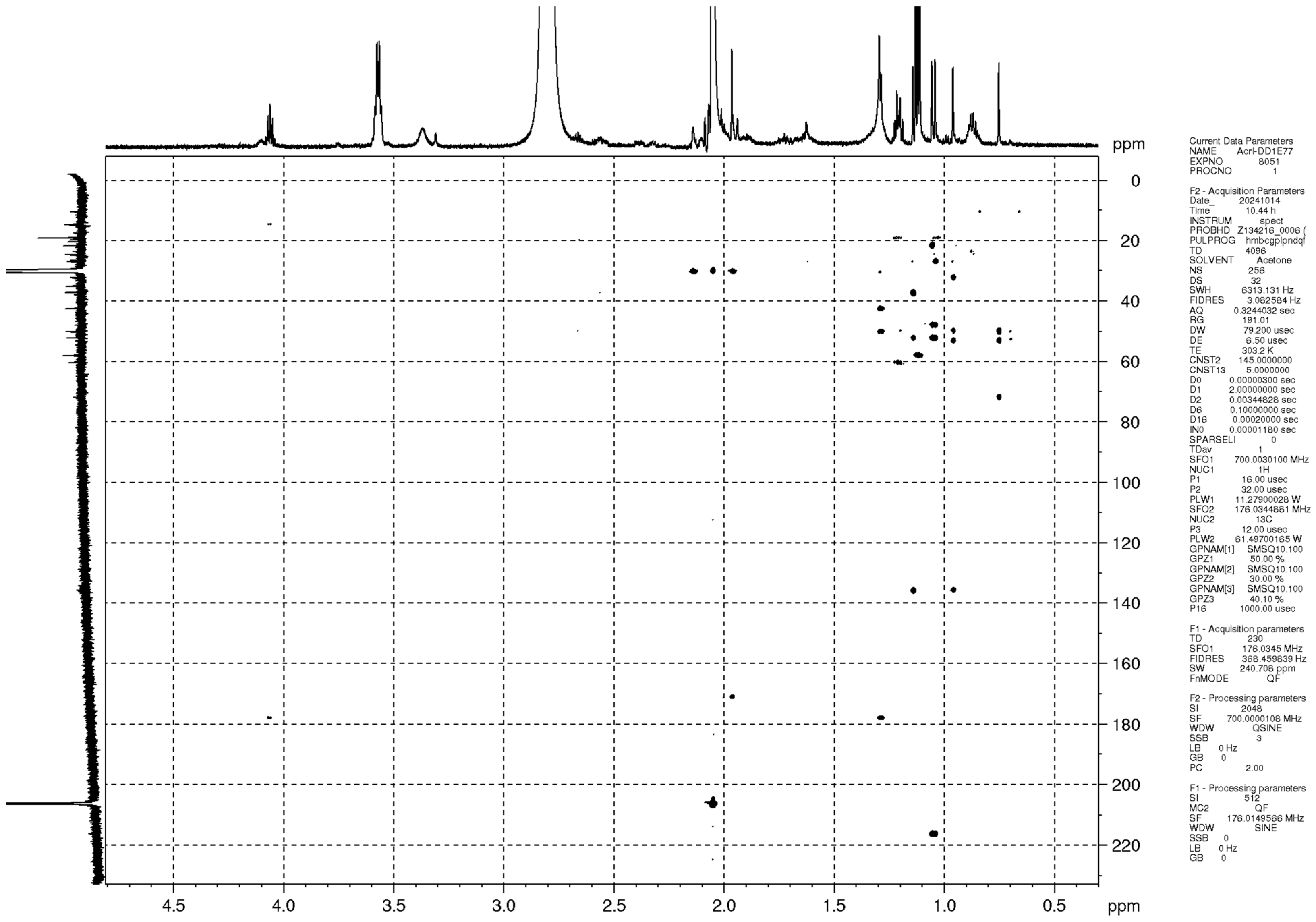

Figure S12. COSY NMR spectrum (700 MHz, acetone-d6) of **2**

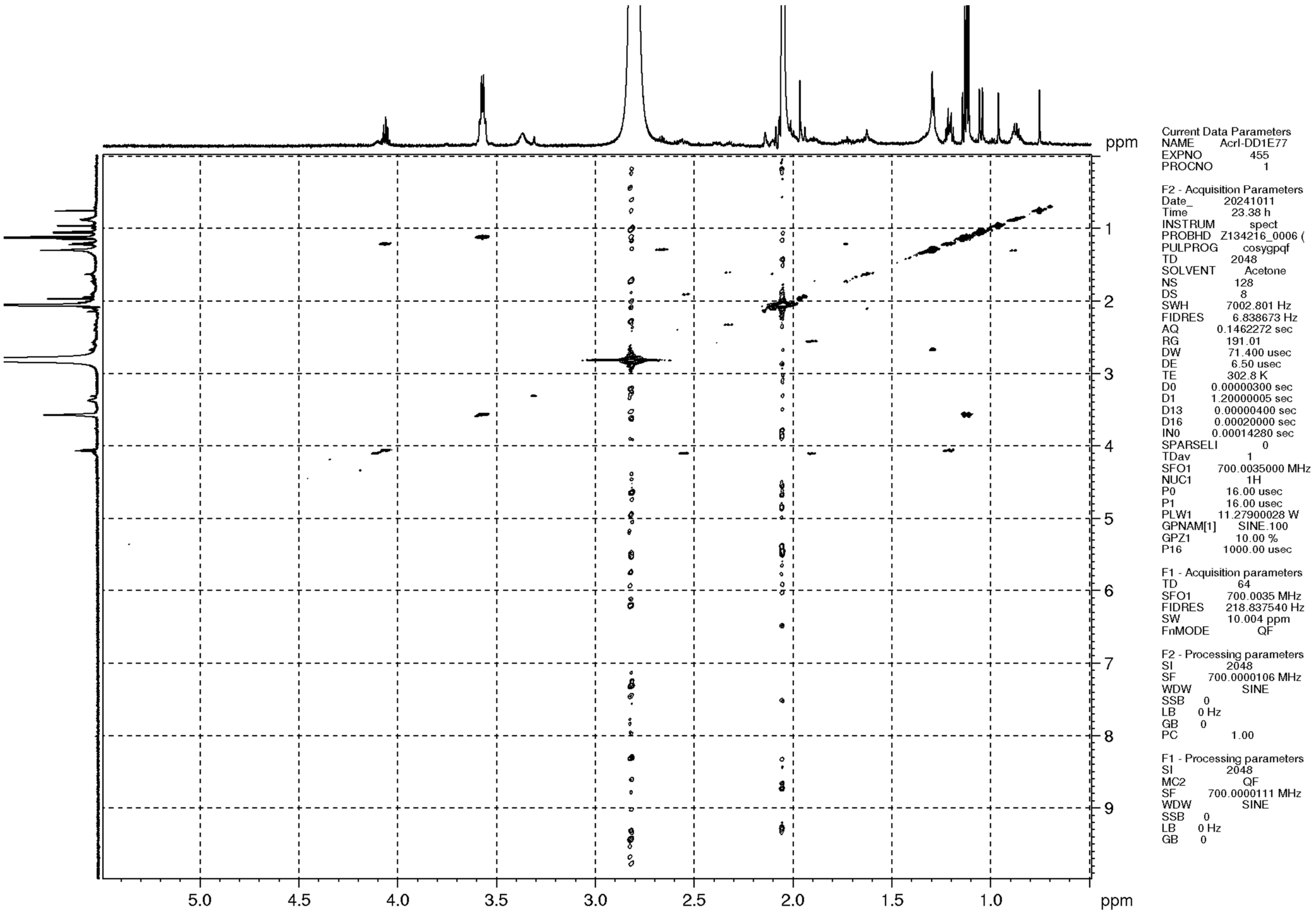

Figure S13. ^{1}H NMR spectrum (500 MHz, acetone-d6) of **3**

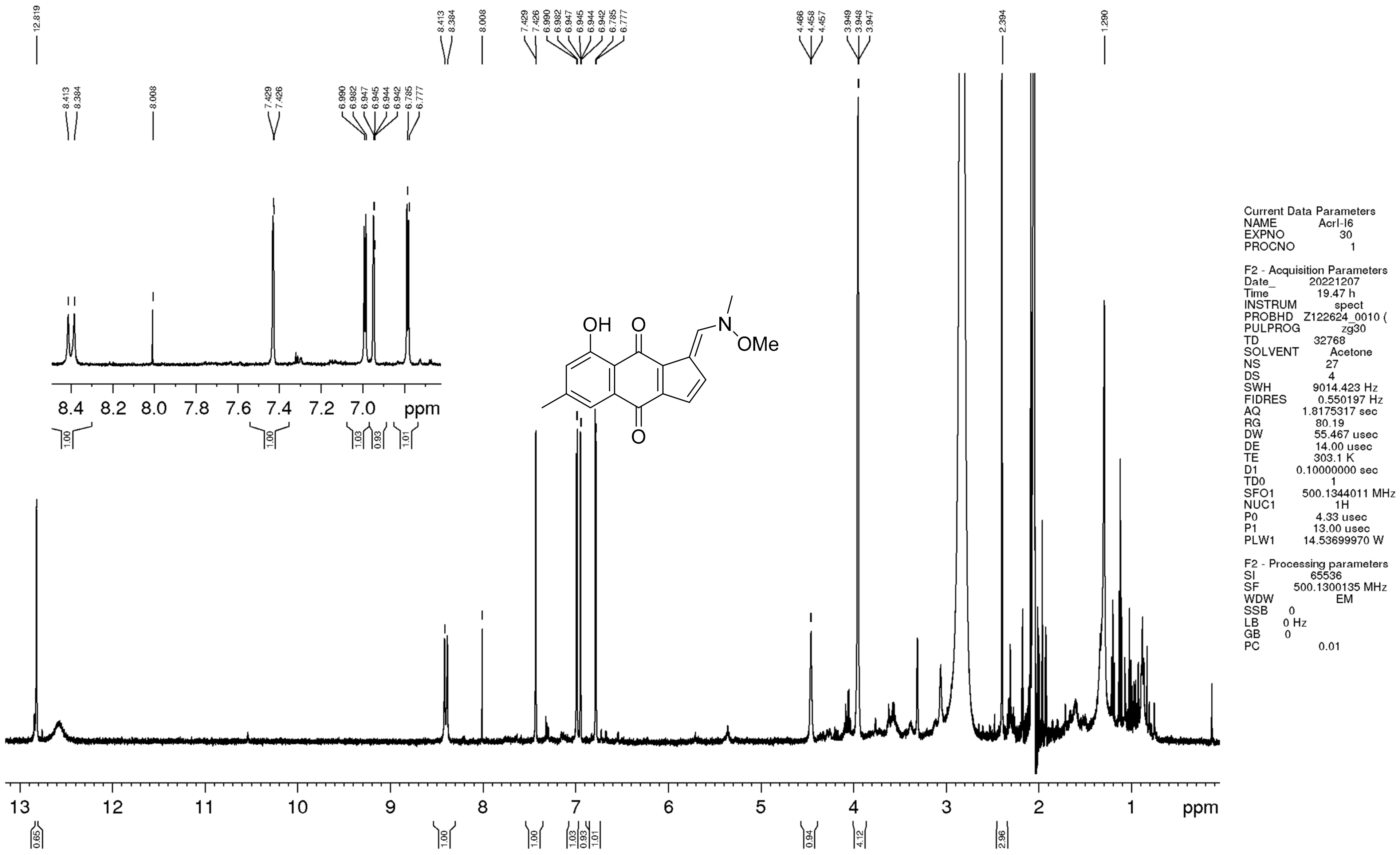

Figure S14. ^{13}C NMR spectrum (500 MHz, acetone-d6) of **3**

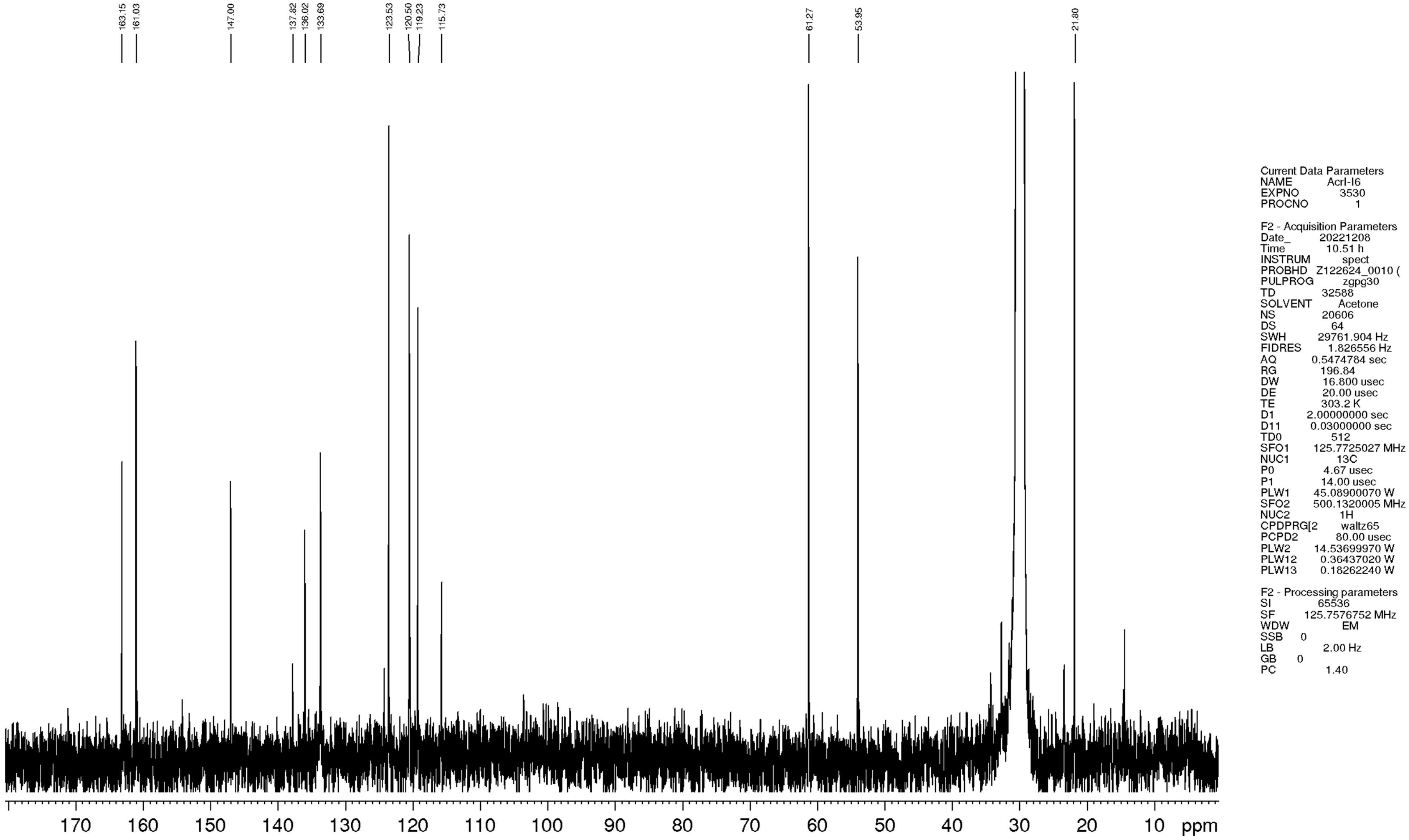

Figure S15. DEPT NMR spectrum (500 MHz, acetone-d6) of **3**

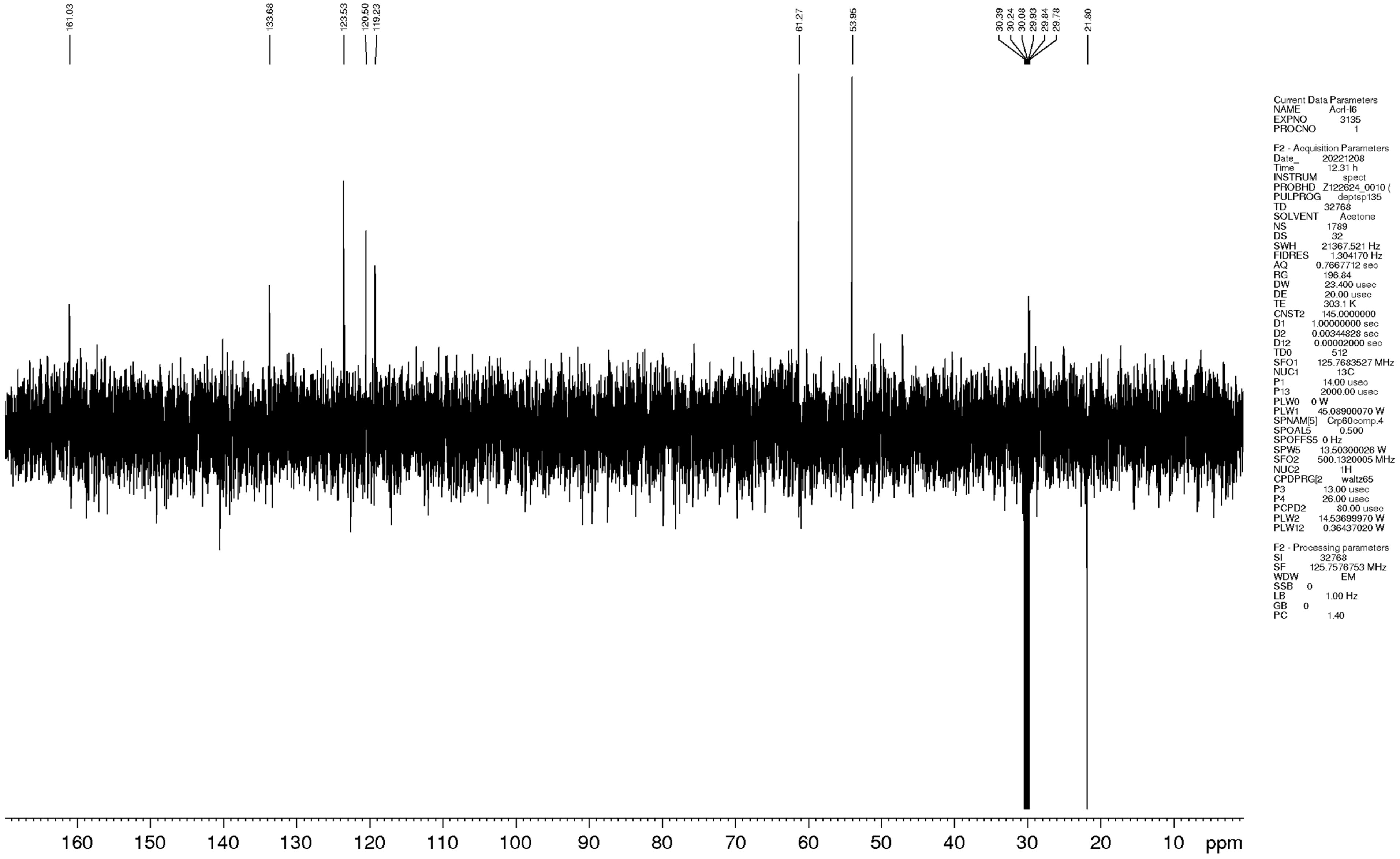

Figure S16. HSQC NMR spectrum (700 MHz, acetone-d6) of **3**

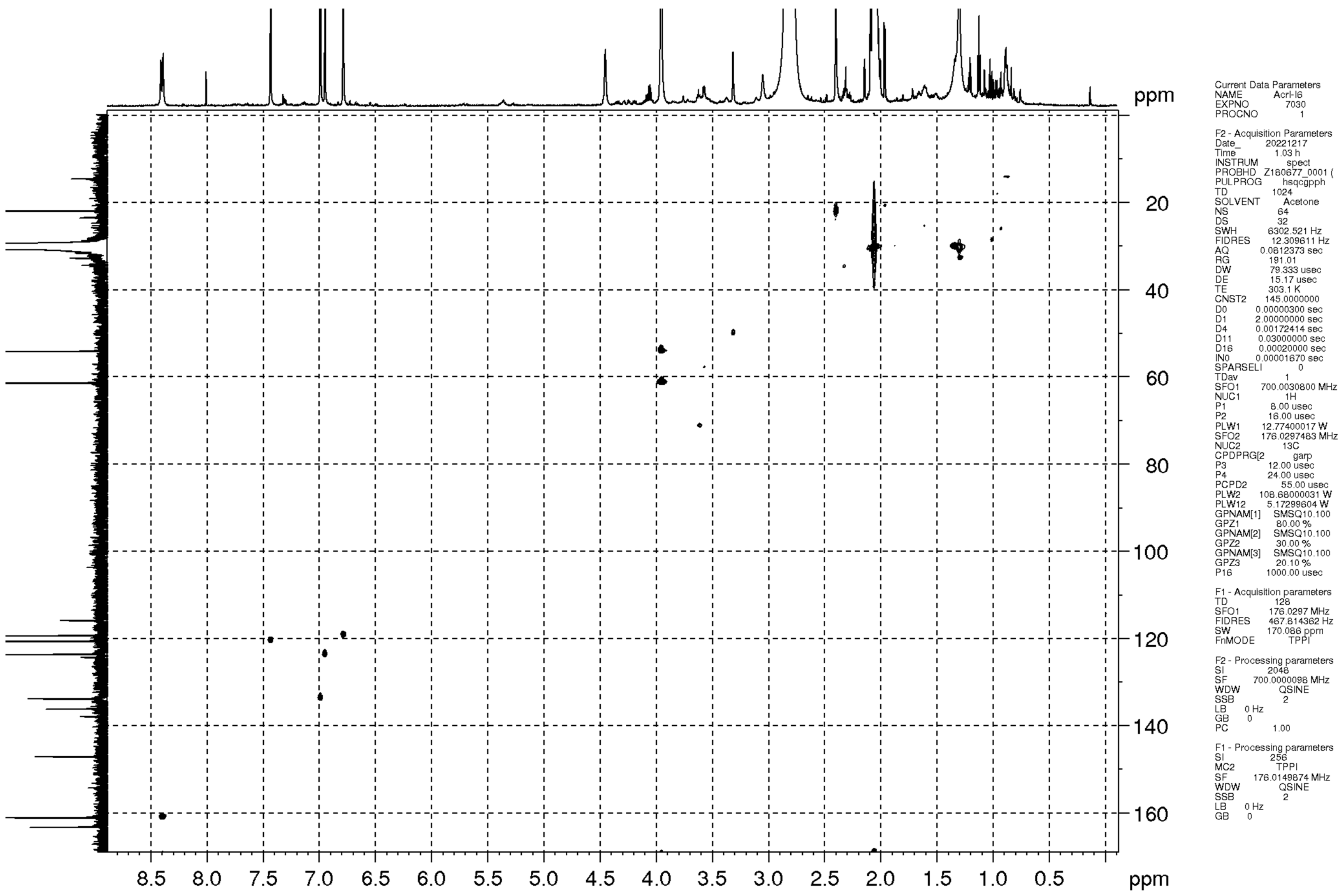

Figure S17. HMBC NMR spectrum (700 MHz, acetone-d6) of **3**

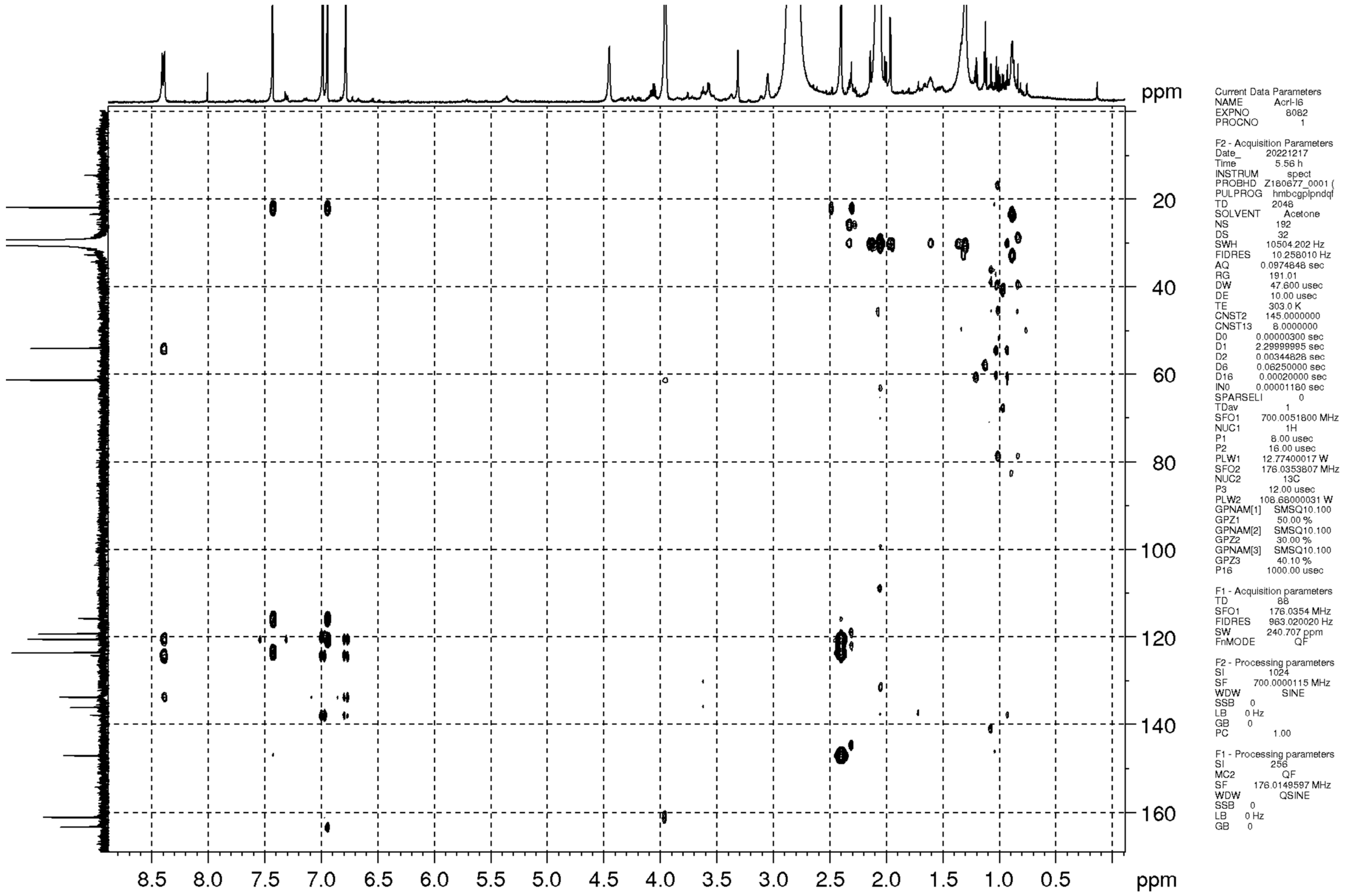

Figure S18. COSY NMR spectrum (700 MHz, acetone-d6) of **3**

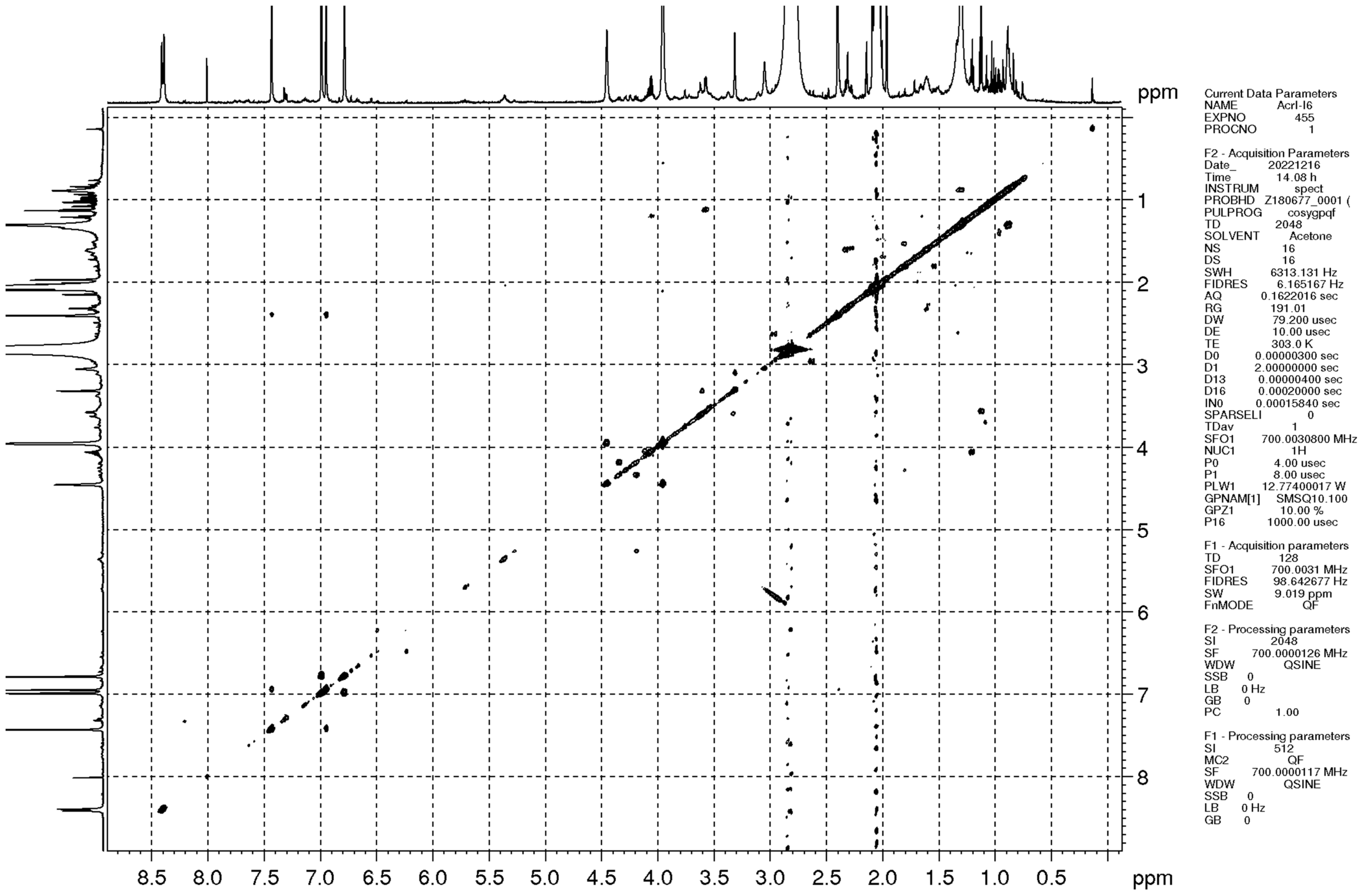

Figure S19. ^{1}H NMR spectrum (500 MHz, acetone-d6) of **4**

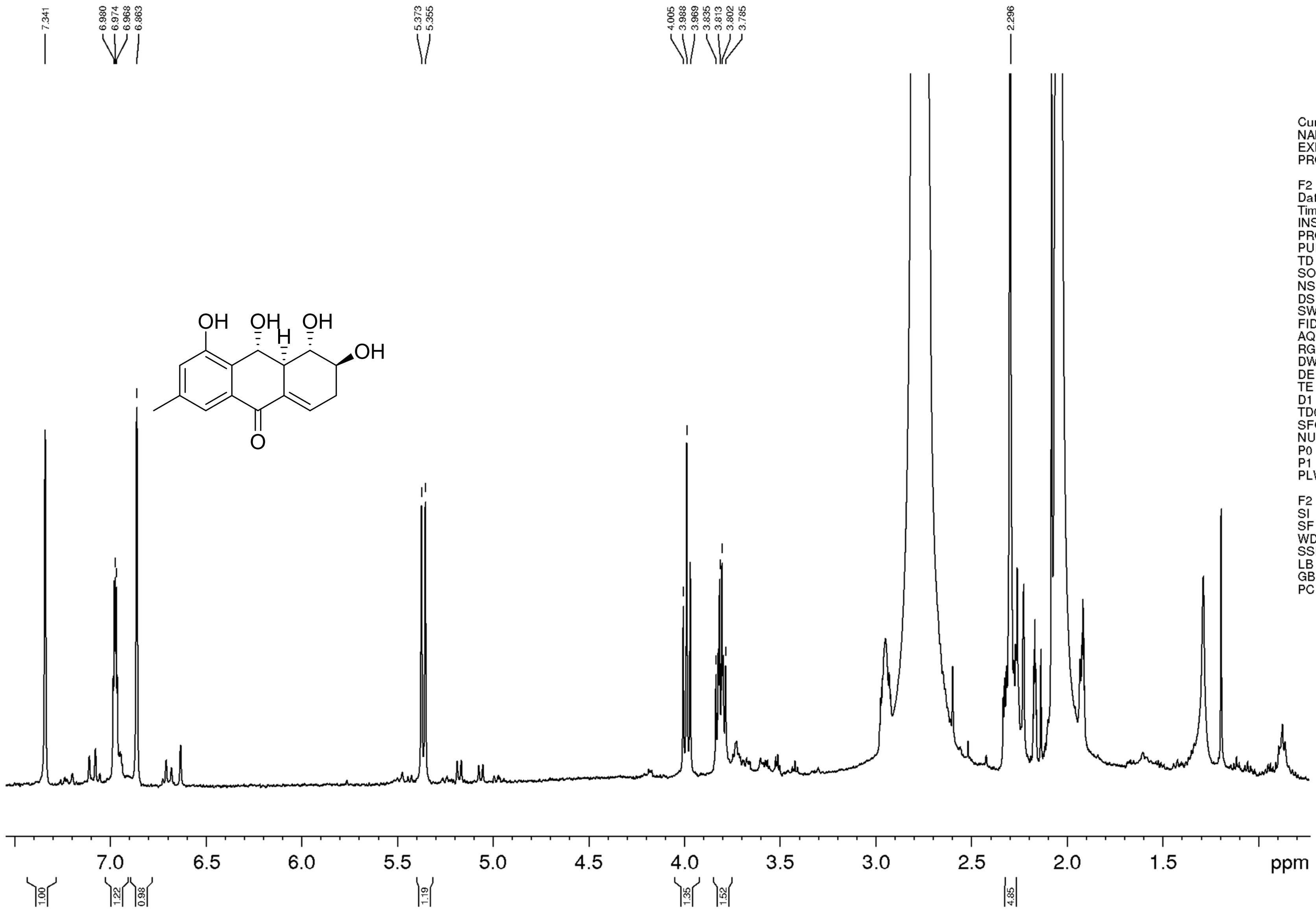

Current Data Parameters
NAME I5-2h-4
EXPNO 30
PROCNO 1

F2 - Acquisition Parameters
Date_ 20240610
Time 13.52 h
INSTRUM spect
PROBHD Z113652_0155 (
PULPROG zg30
TD 16384
SOLVENT Acetone
NS 128
DS 2
SWH 8012.820 Hz
FIDRES 0.978127 Hz
AQ 1.0223616 sec
RG 99.34
DW 62.400 usec
DE 14.00 usec
TE 303.1 K
D1 0.20000000 sec
TD0 1
SFO1 500.1338510 MHz
NUC1 1H
P0 4.33 usec
P1 13.00 usec
PLW1 14.53699970 W

F2 - Processing parameters
SI 65536
SF 500.1300165 MHz
WDW EM
SSB 0
LB 1.00 Hz
GB 0
PC 1.00

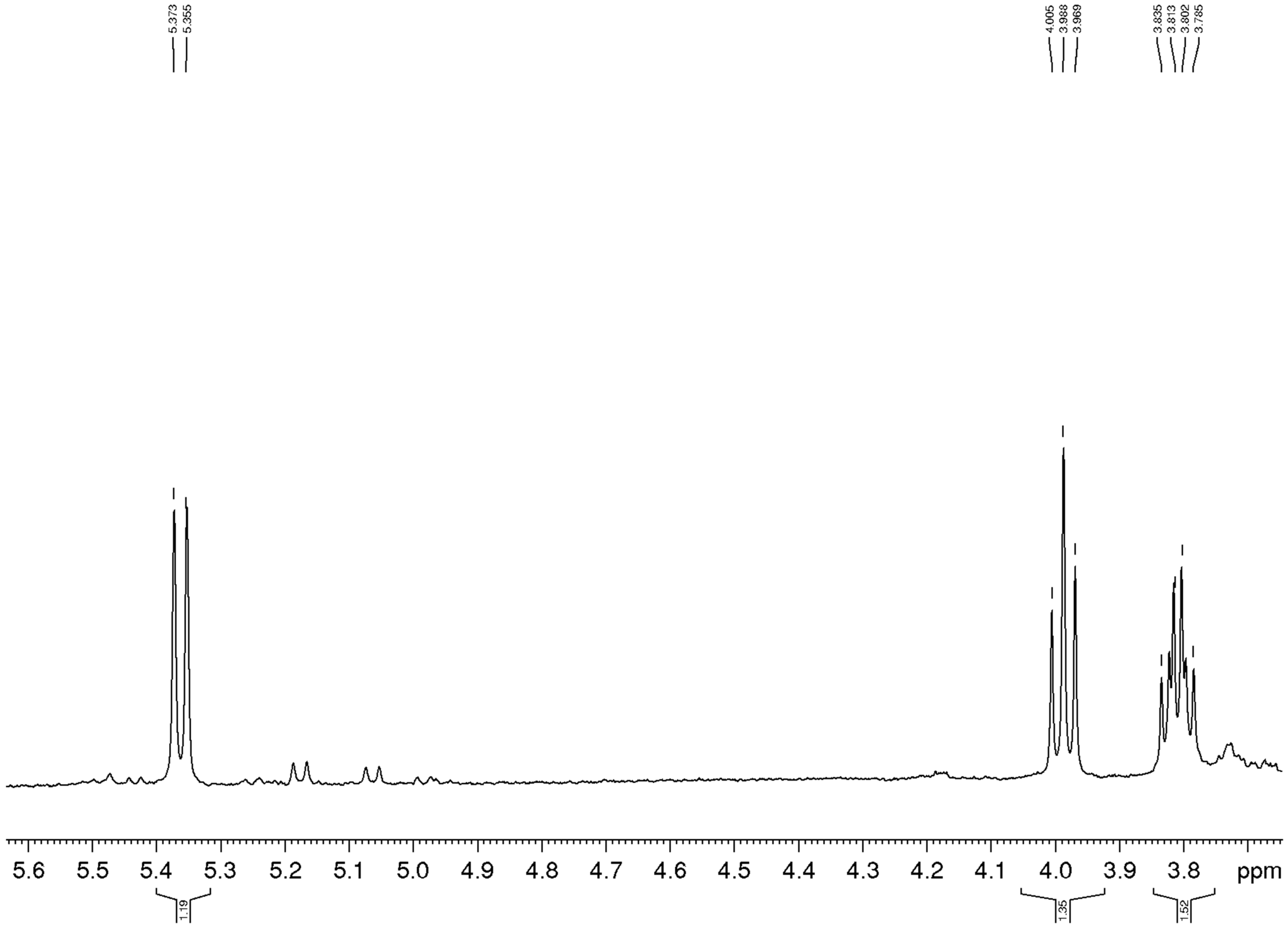

Figure S20. ^{13}C NMR spectrum (500 MHz, acetone-d6) of **4**

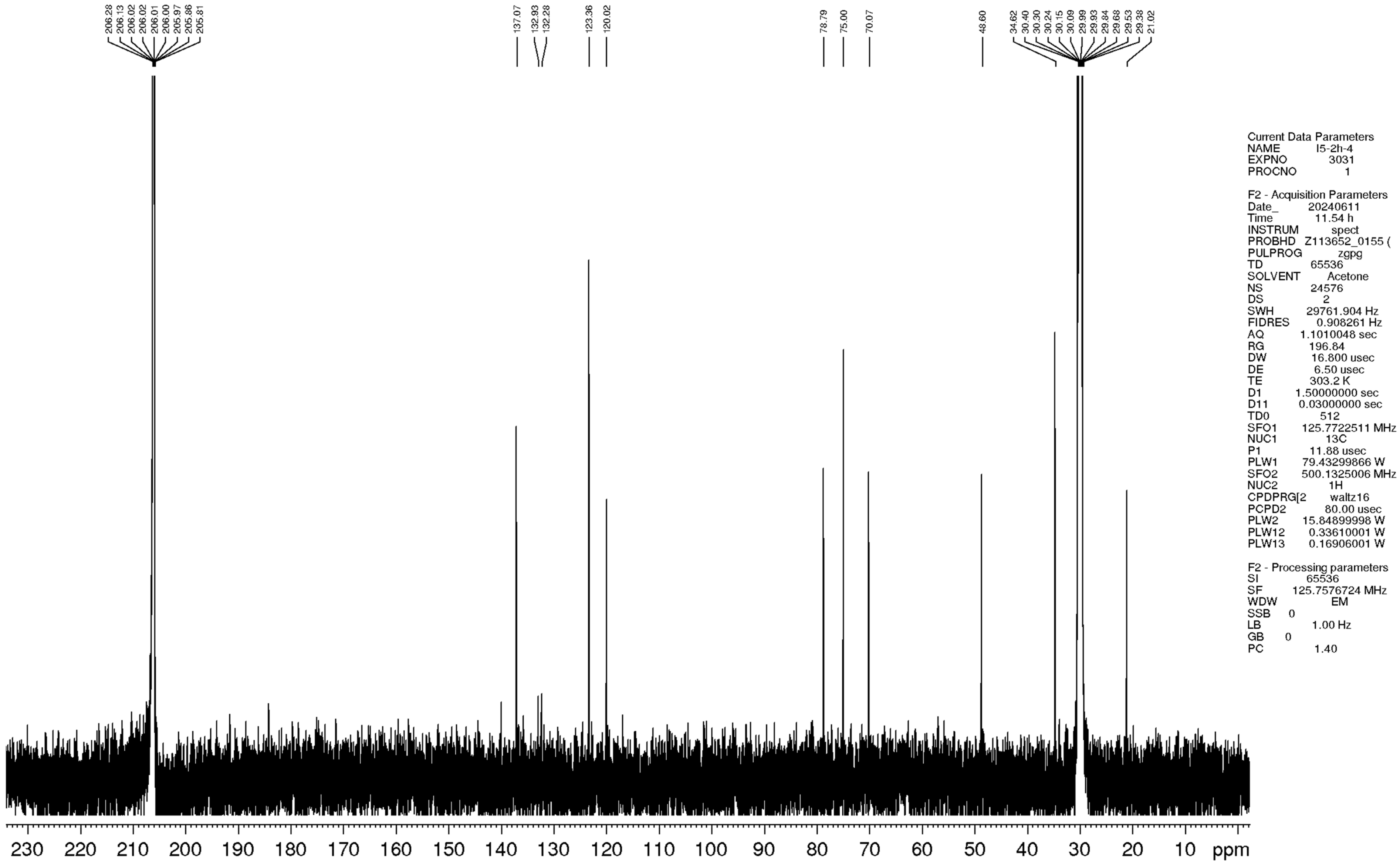

Figure S21. DEPT NMR spectrum (500 MHz, acetone-d6) of **4**

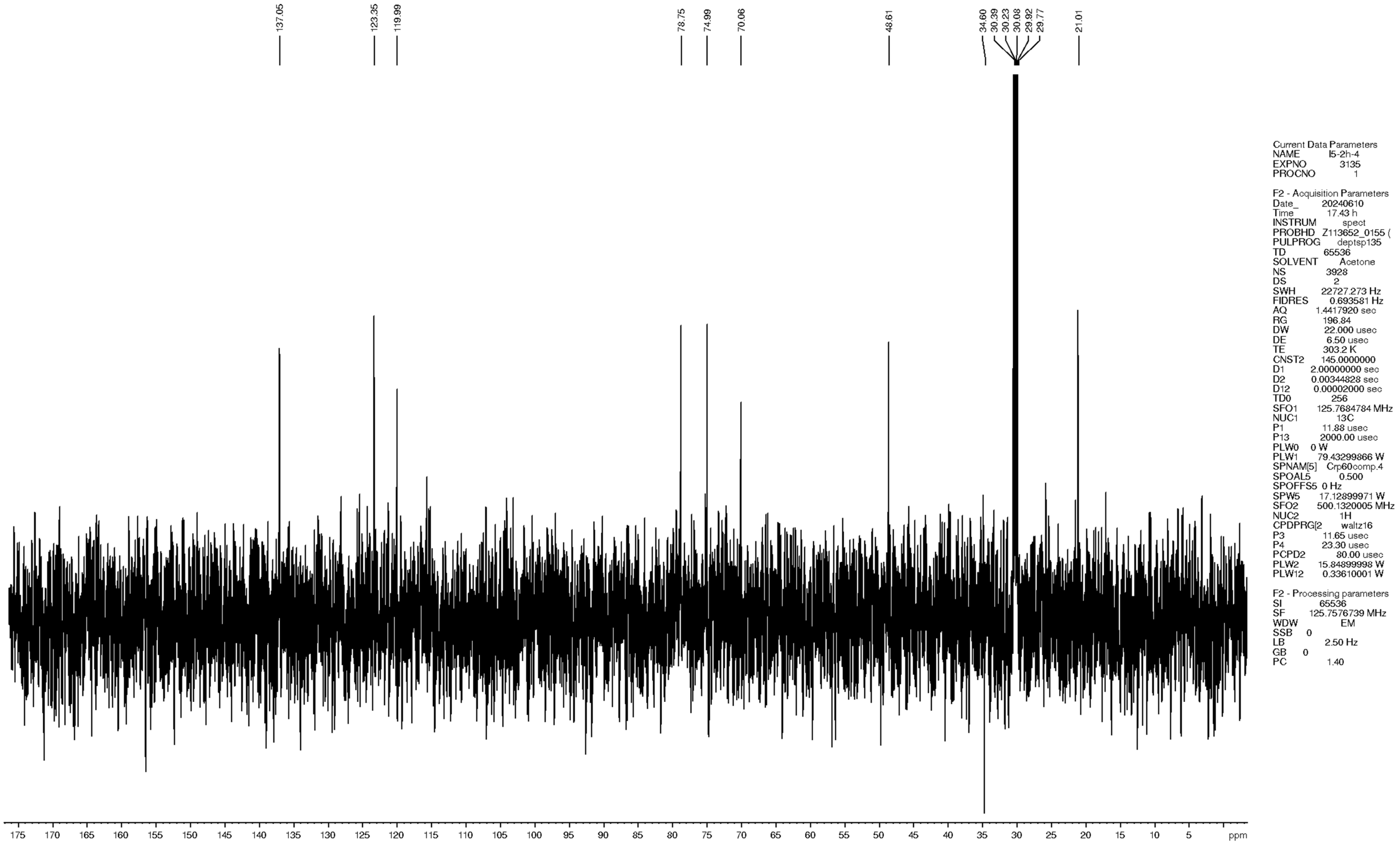

Figure S22. ^{1}H NMR spectrum (500 MHz, acetone-d6) of **5**

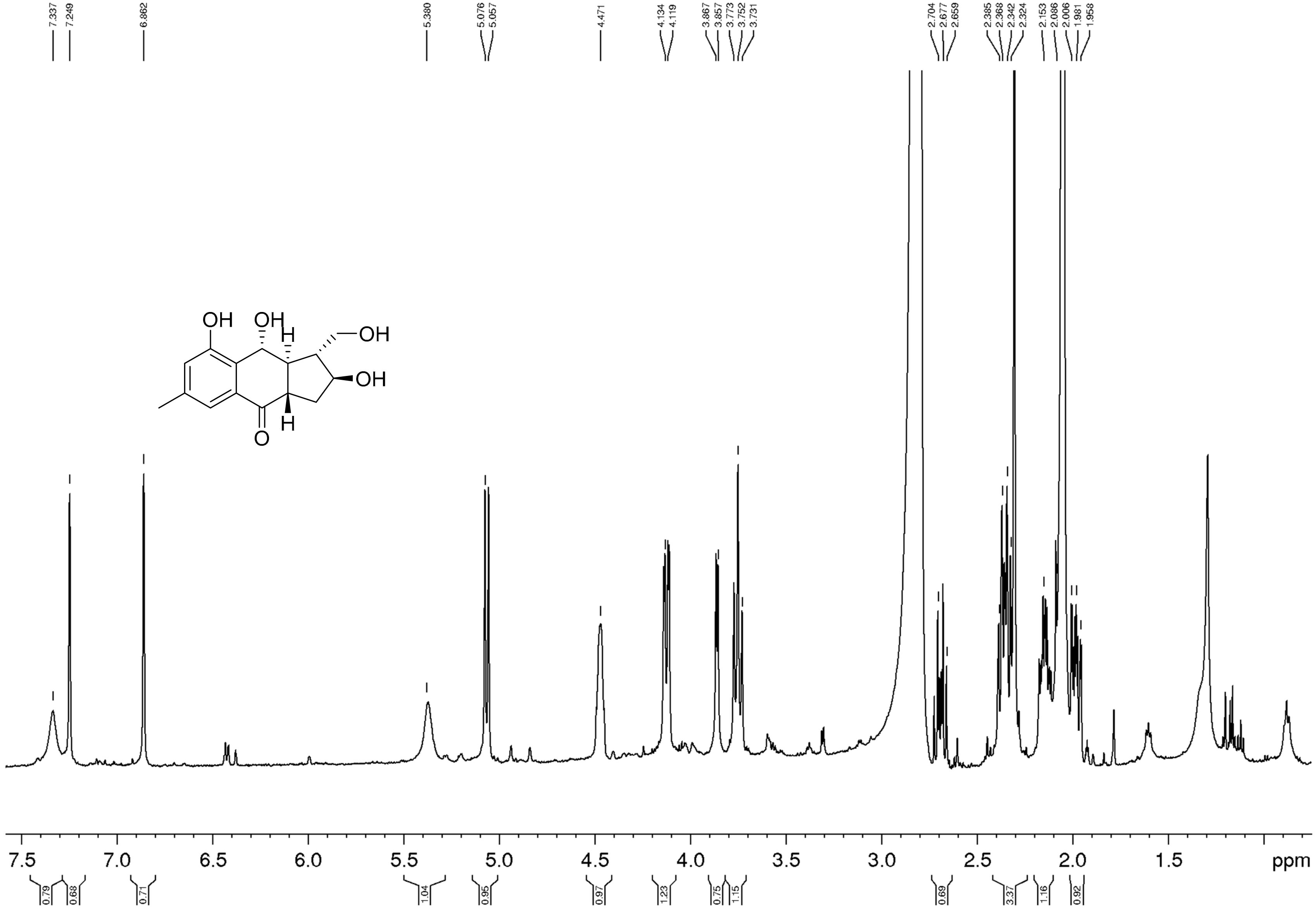

Current Data Parameters
NAME Acrl-L2
EXPNO 31
PROCNO 1

F2 - Acquisition Parameters
Date_ 20230307
Time 16.59 h
INSTRUM spect
PROBHD Z122624_0010 (
PULPROG zg
TD 16384
SOLVENT Acetone
NS 8
DS 0
SWH 8012.820 Hz
FIDRES 0.978127 Hz
AQ 1.0223616 sec
RG 119.65
DW 62.400 usec
DE 14.00 usec
TE 303.1 K
D1 0.20000000 sec
TD0 1
SFO1 500.1332508 MHz
NUC1 1H
P1 13.00 usec
PLW1 14.53699970 W

F2 - Processing parameters
SI 65536
SF 500.1300136 MHz
WDW EM
SSB 0
LB 1.00 Hz
GB 0
PC 4.00

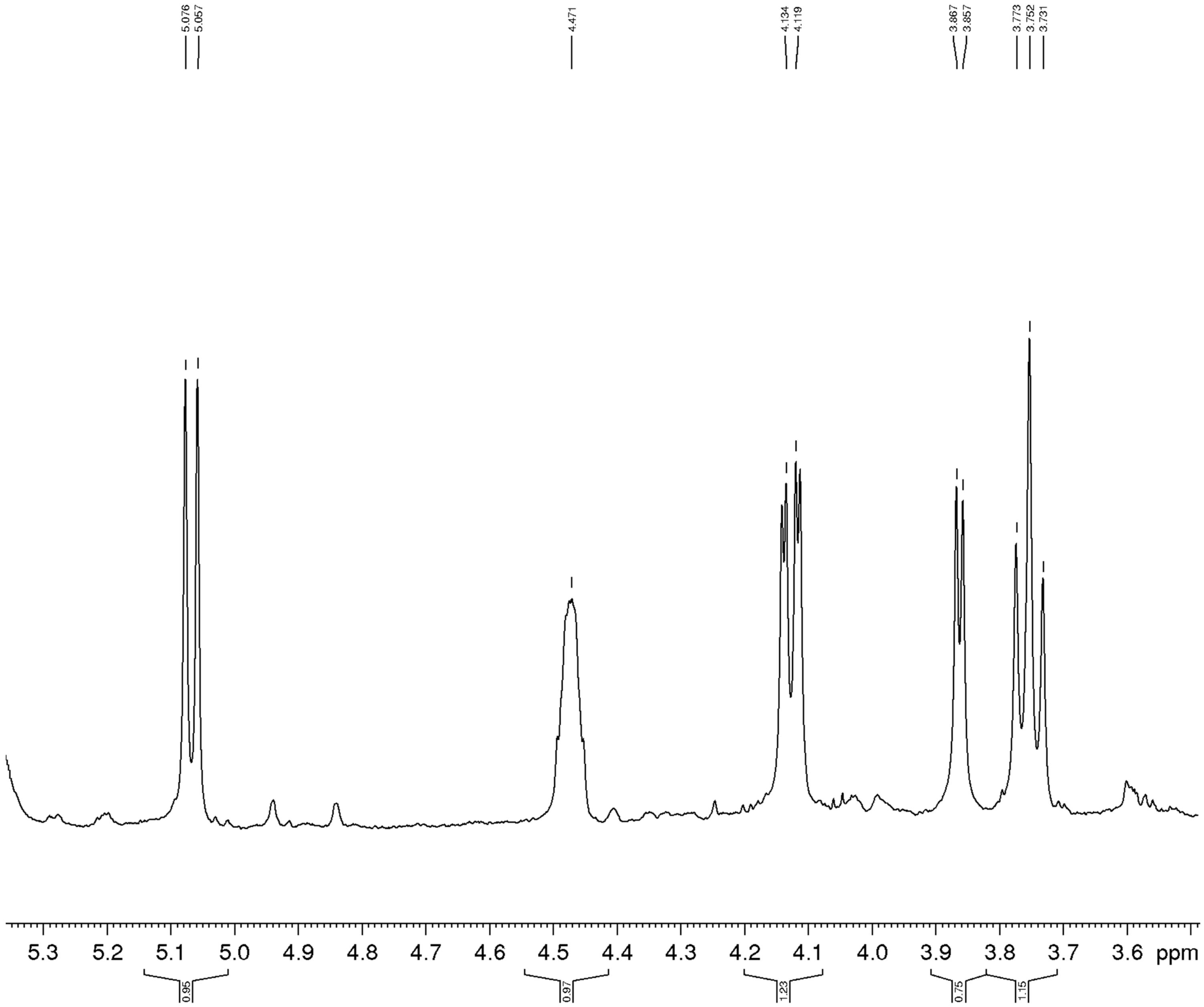

Figure S23. ^{13}C NMR spectrum (500 MHz, acetone-d6) of **5**

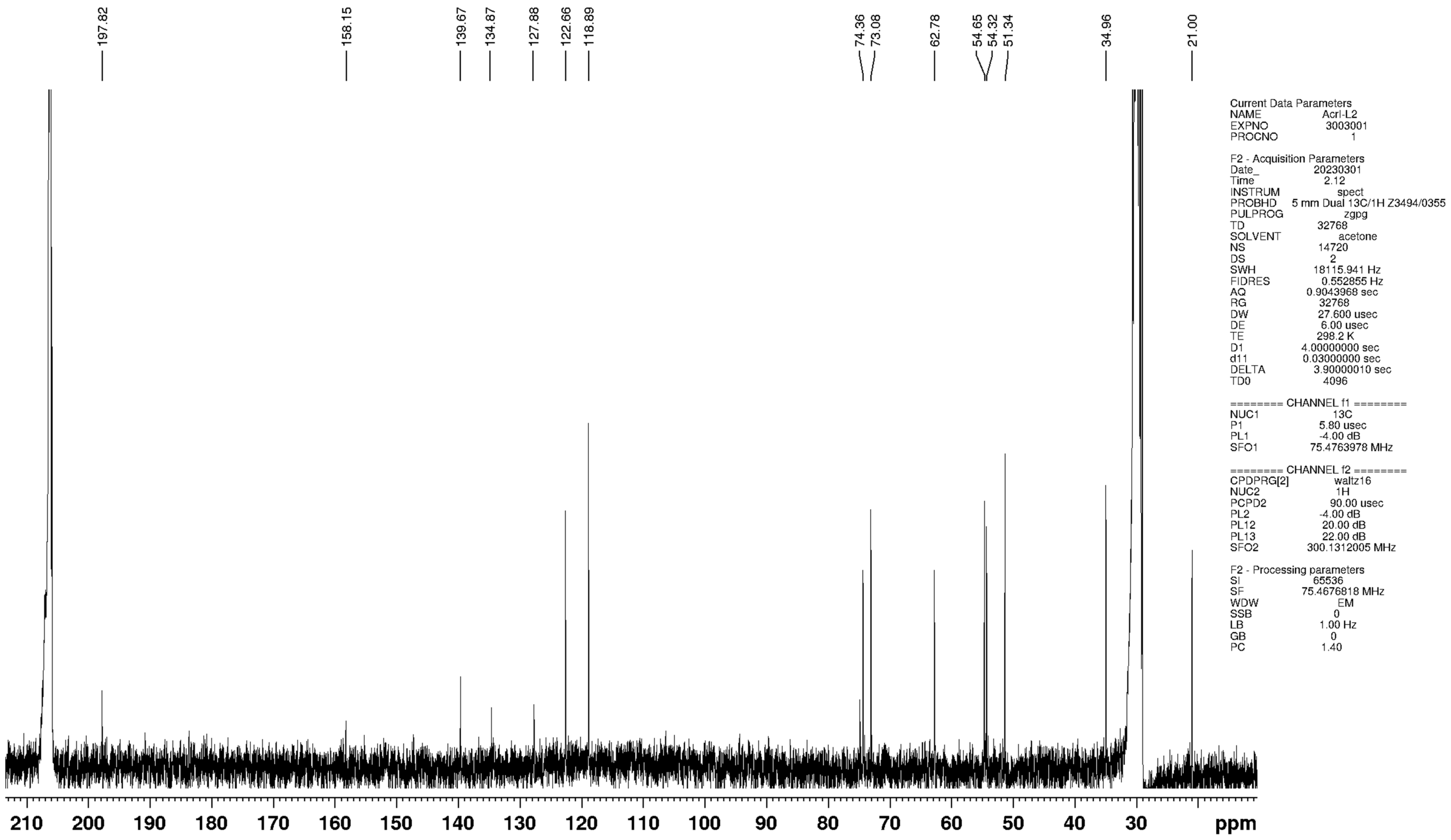

Figure S24. DEPT NMR spectrum (500 MHz, acetone-d6) of **5**

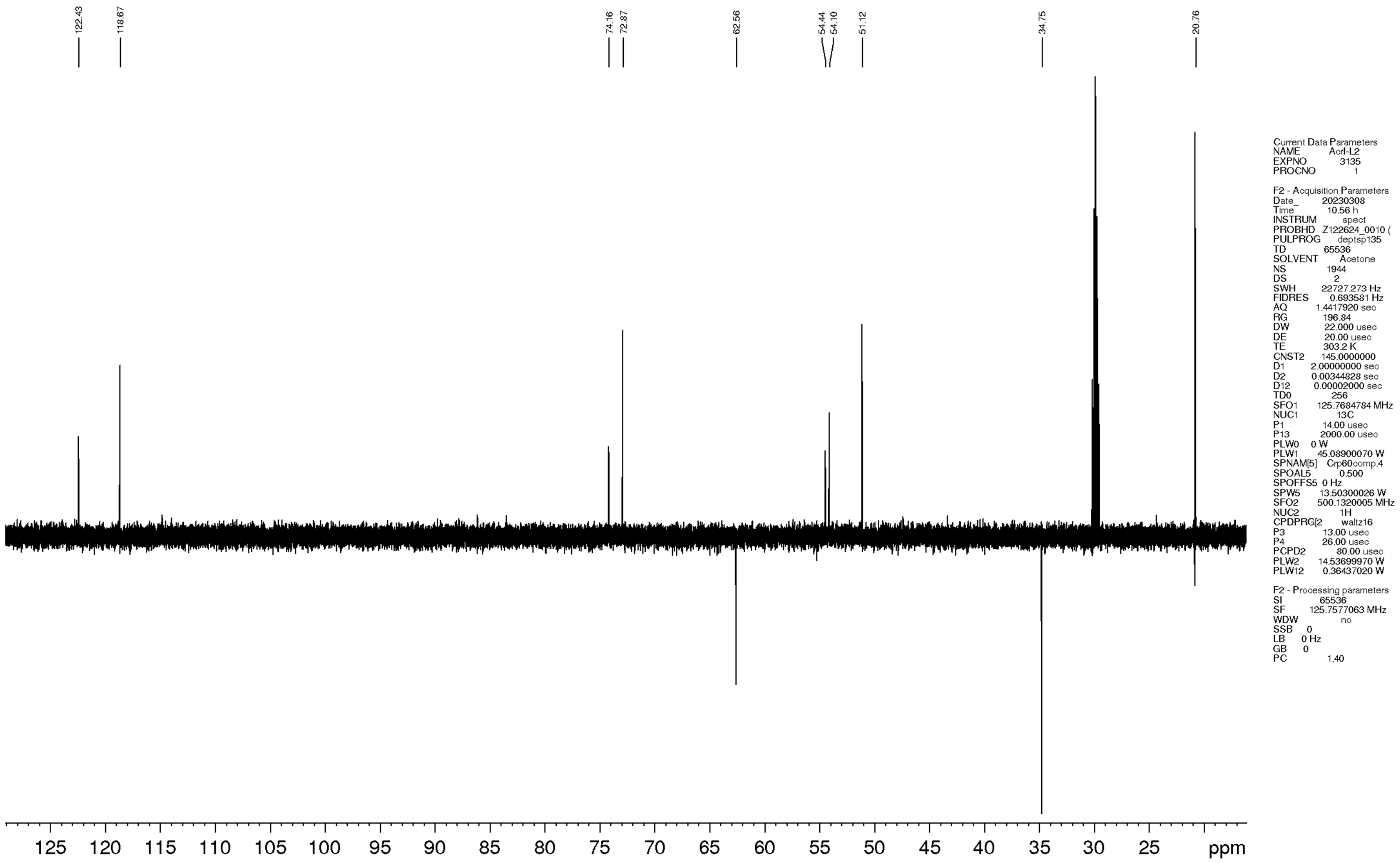

Figure S25. ^{1}H NMR spectrum (500 MHz, $CDCl_3$) of **6**

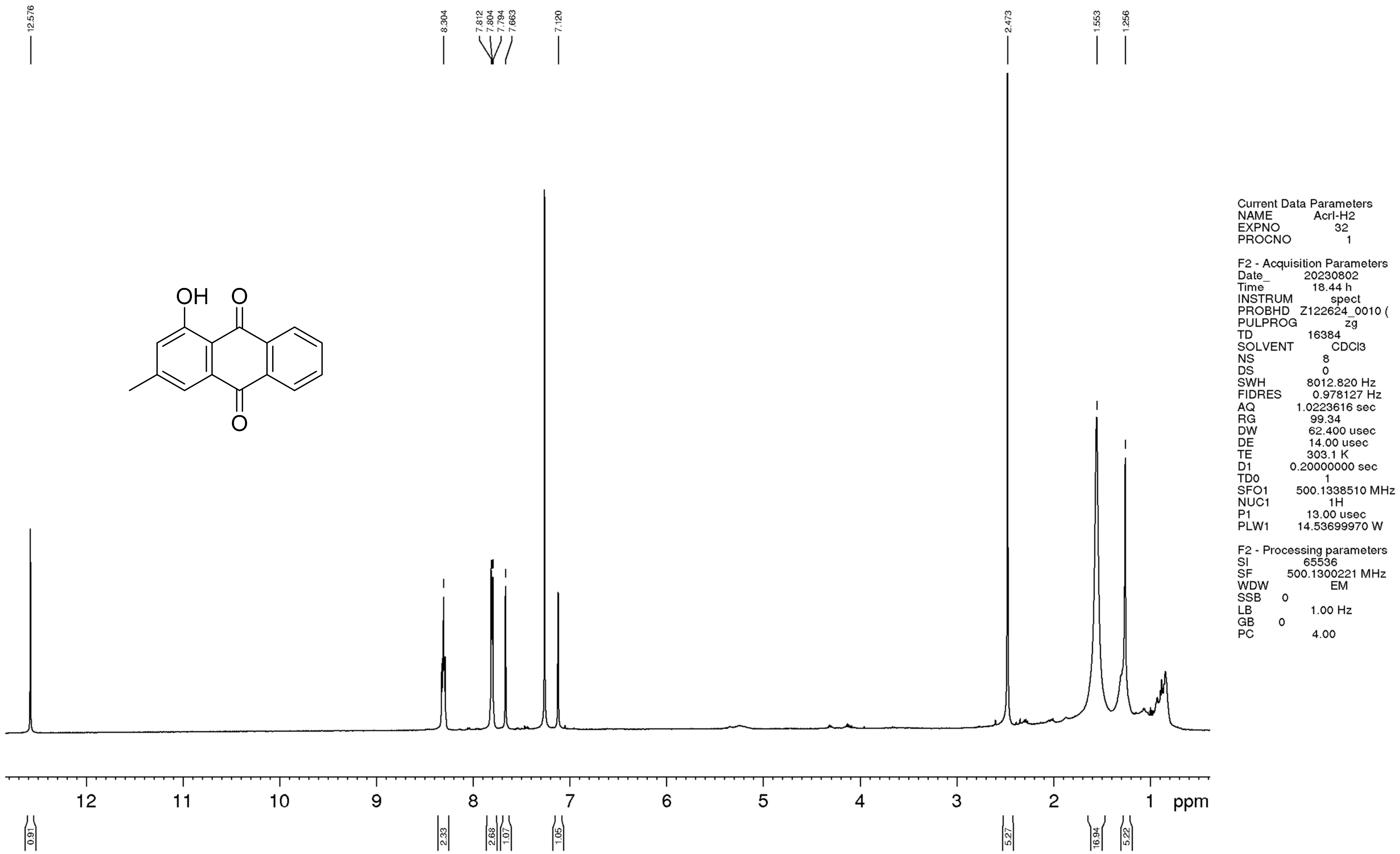

Figure S26. ^{13}C NMR spectrum (500 MHz, $CDCl_3$) of **6**

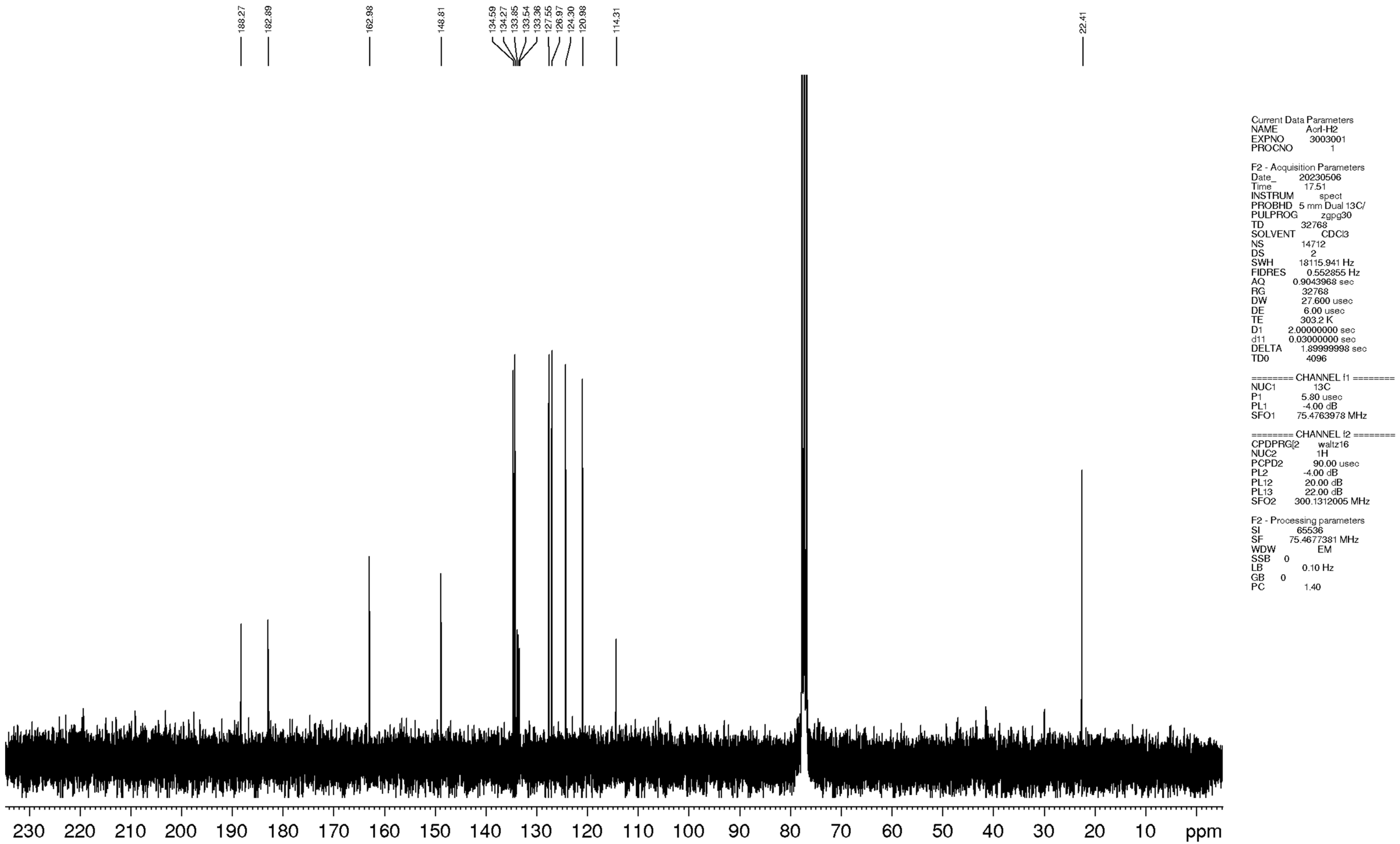

Figure S27. ^{1}H NMR spectrum (500 MHz, acetone-d6) of **7**

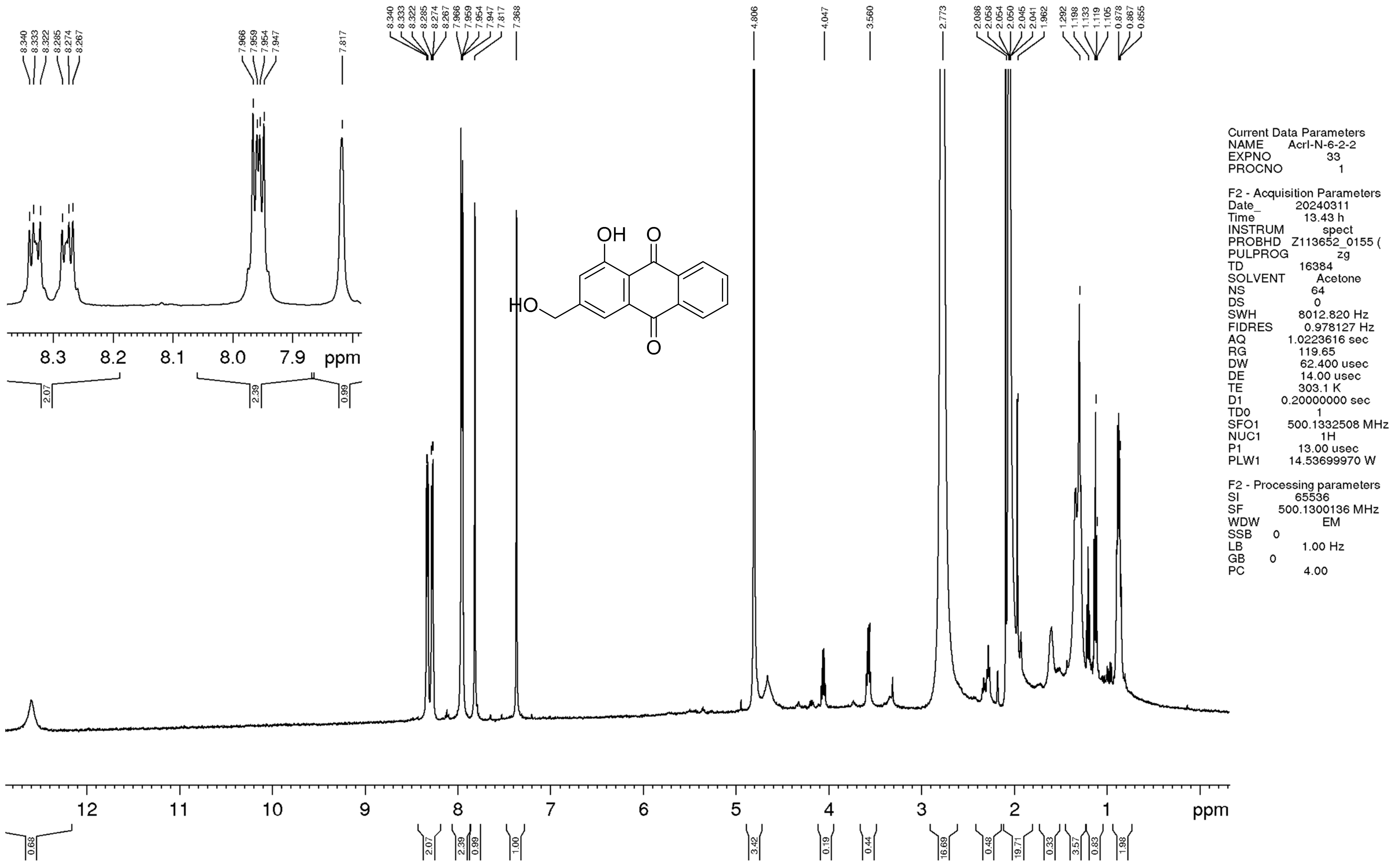

Figure S28. ^{13}C NMR spectrum (500 MHz, acetone-d6) of **7**

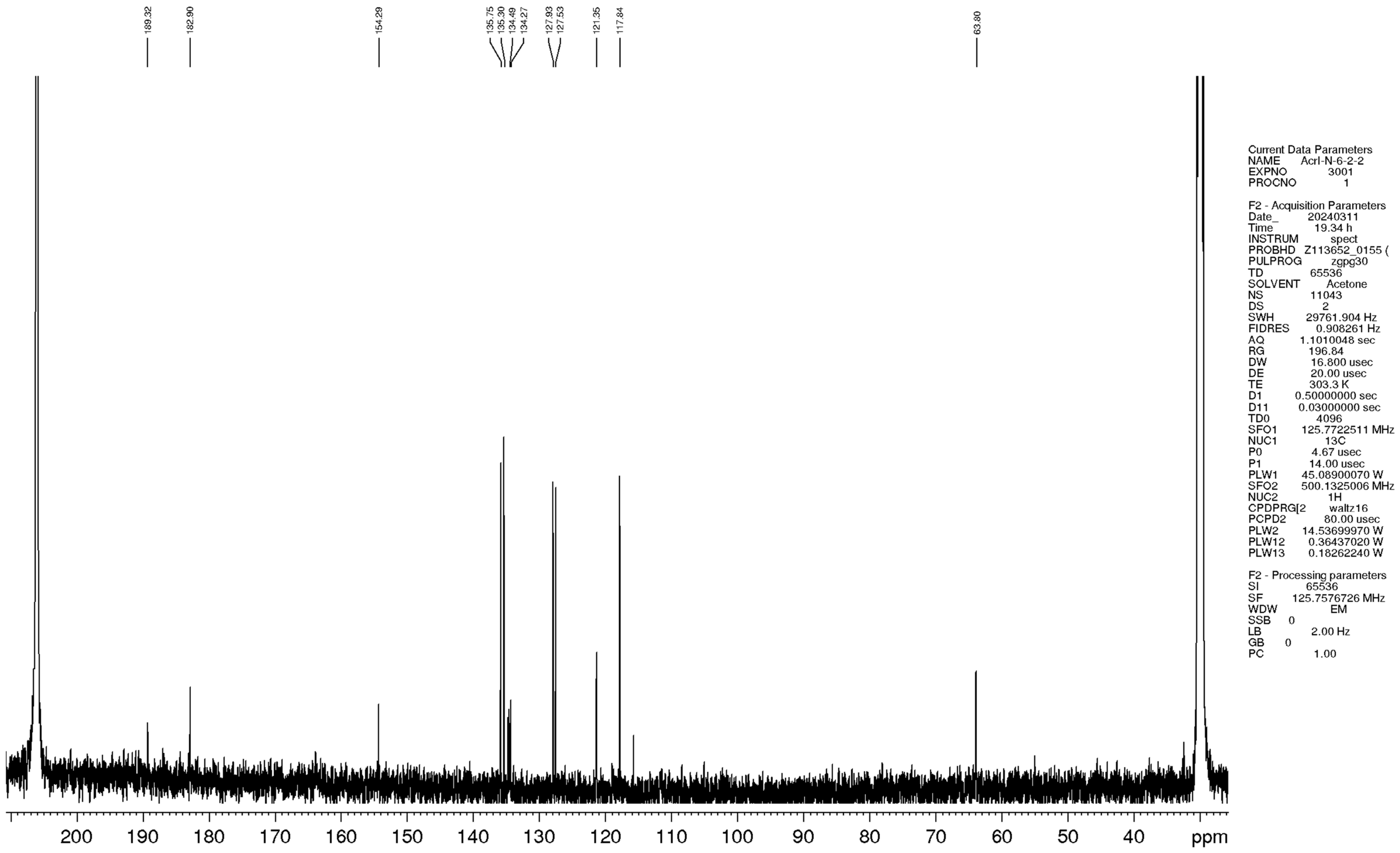

Figure S29. DEPT NMR spectrum (500 MHz, acetone-d6) of **7**

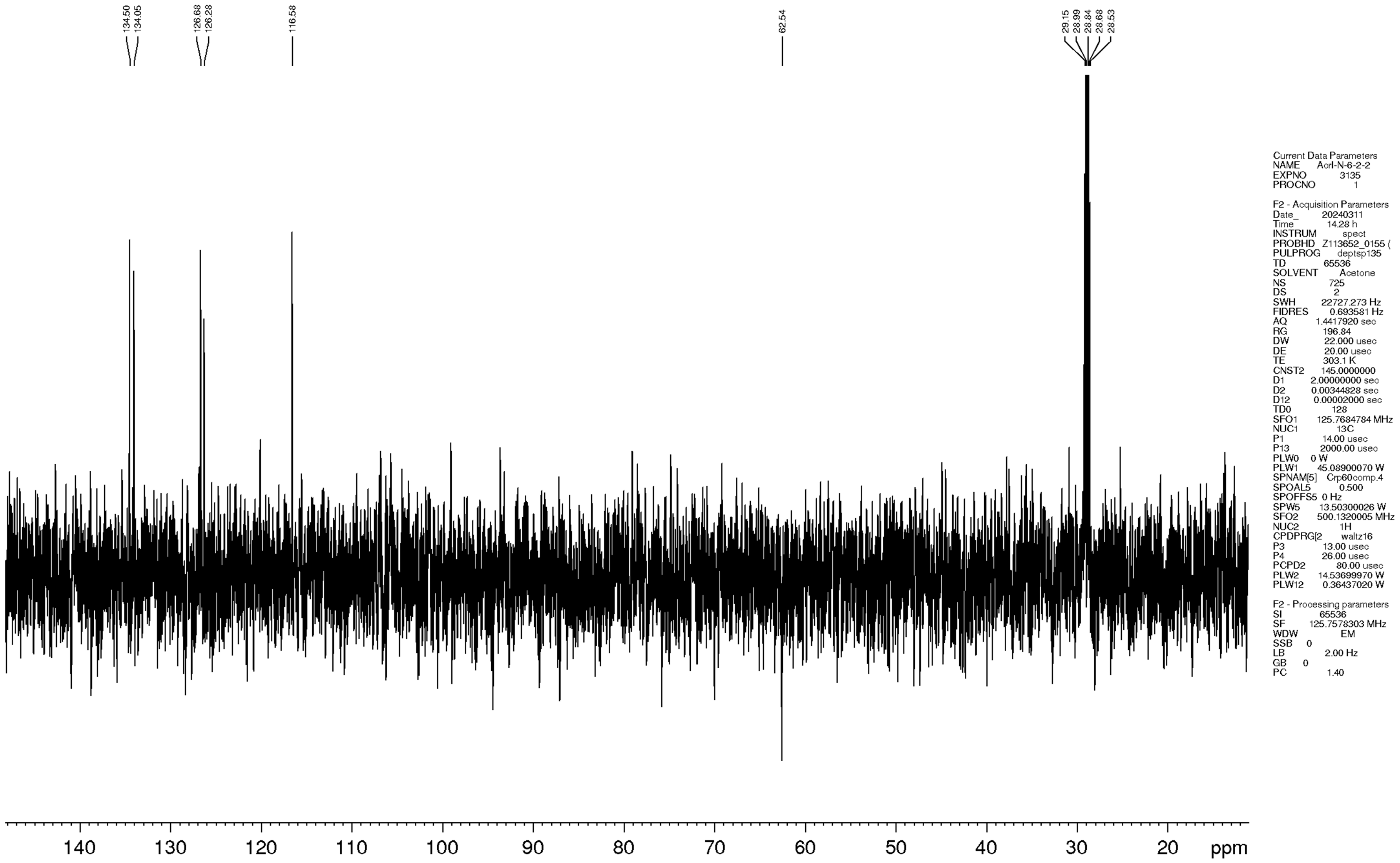

Figure S30. ^{1}H NMR spectrum (500 MHz, acetone-d6) of **8**

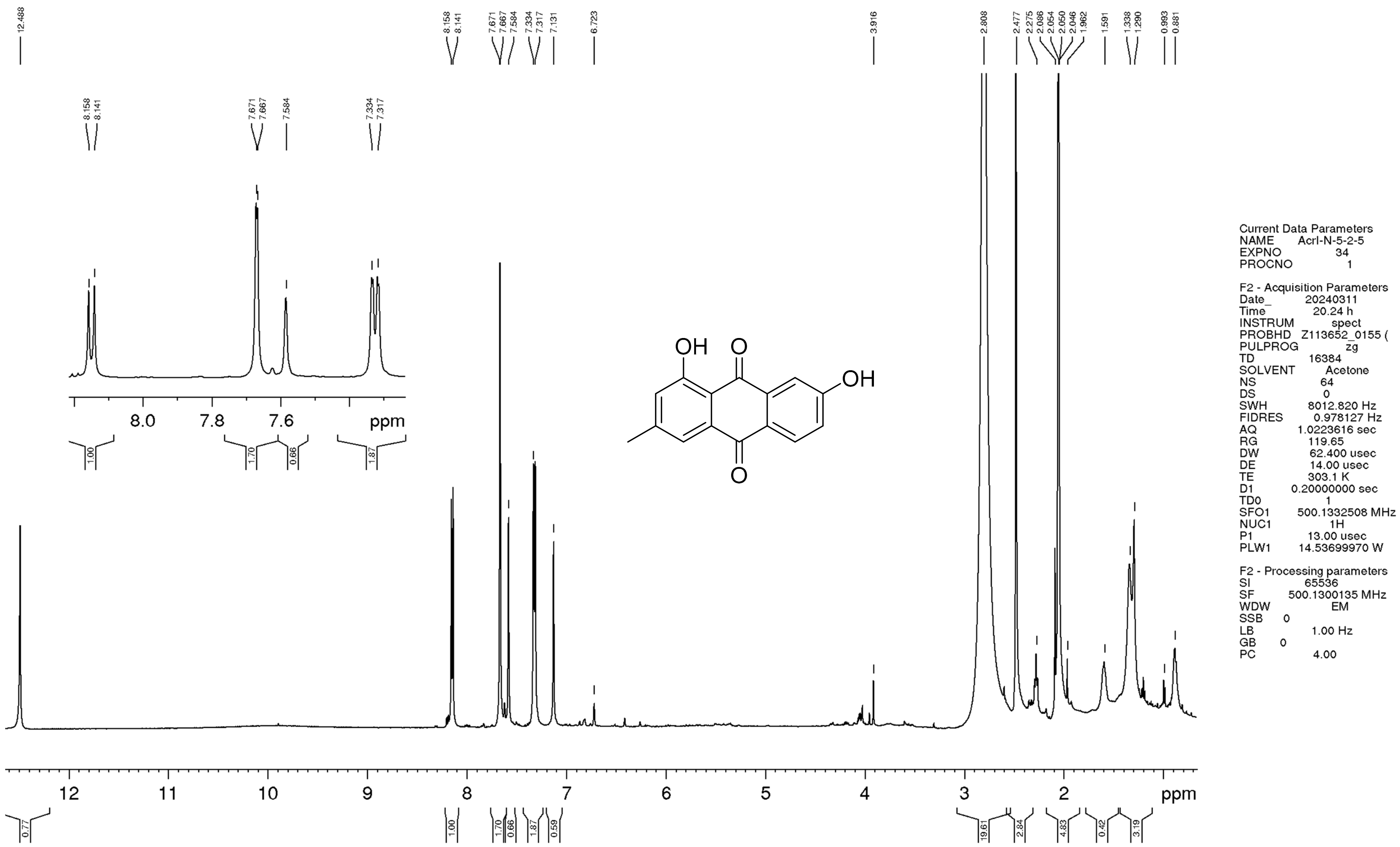

Figure S31. ^{13}C NMR spectrum (500 MHz, acetone-d6) of **8**

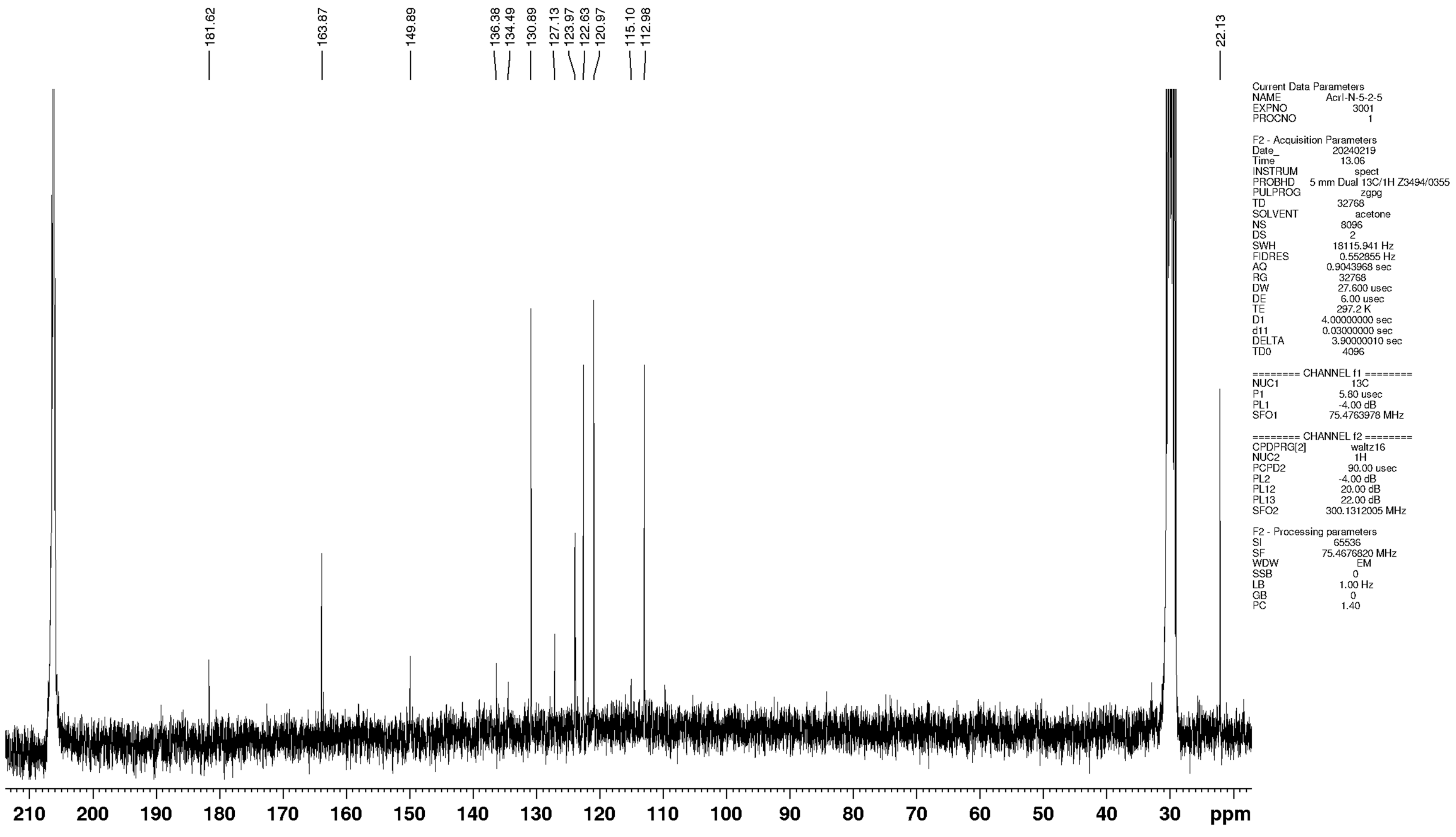

Figure S32. DEPT NMR spectrum (500 MHz, acetone-d6) of **8**

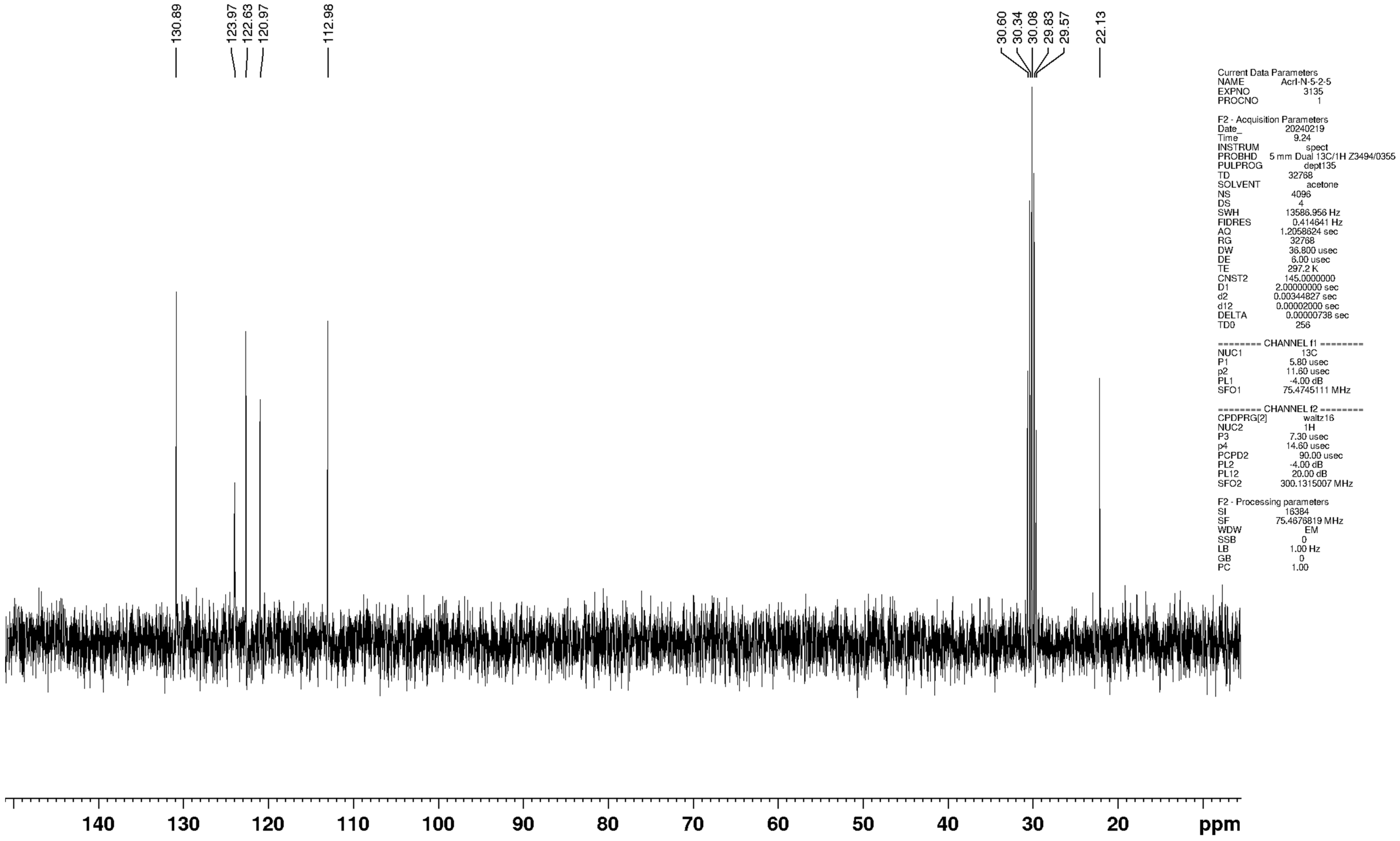

Figure S33. ^{1}H NMR spectrum (500 MHz, CD_3OD) of **10**

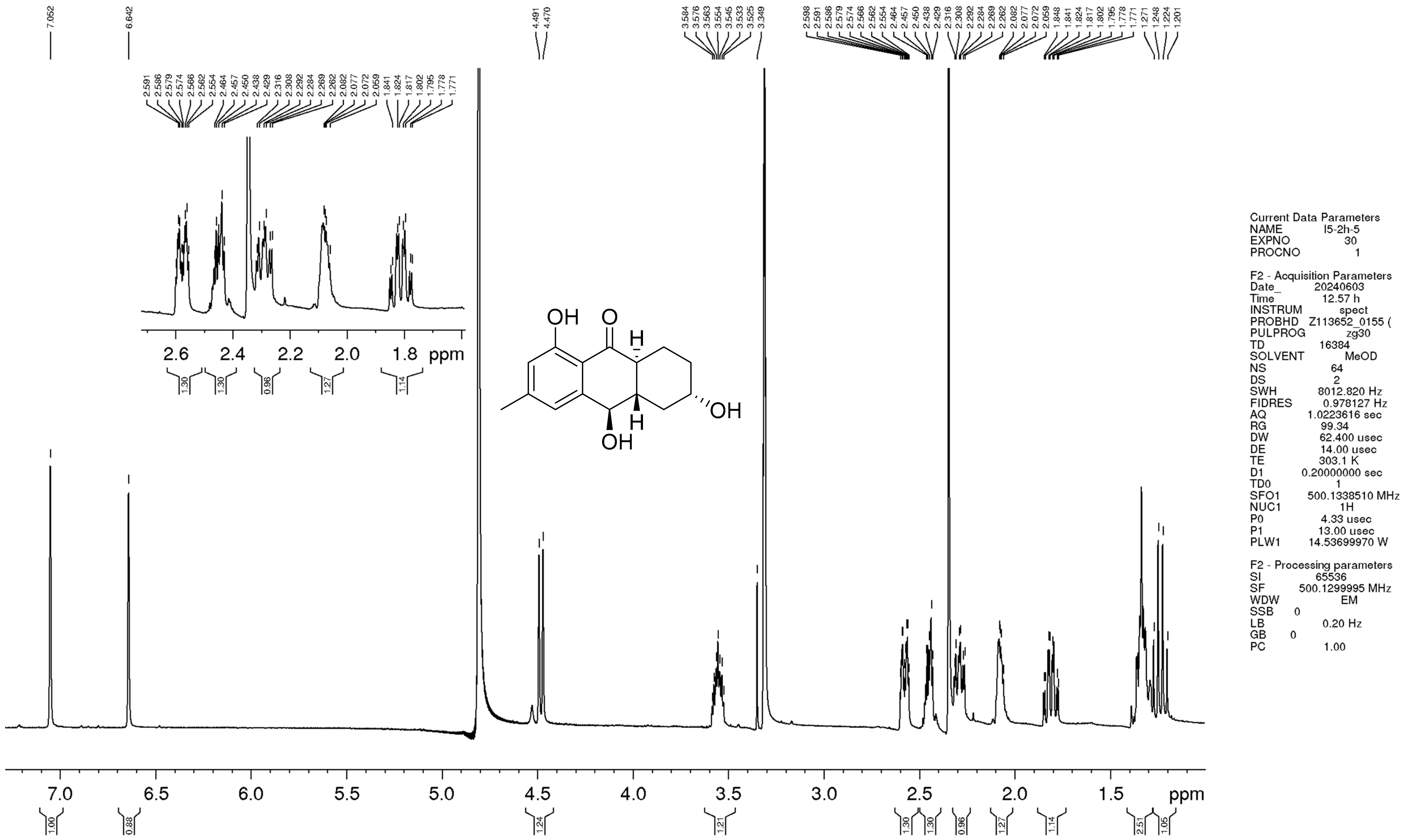

Figure S34. ^{13}C NMR spectrum (500 MHz, CD_3OD) of **10**

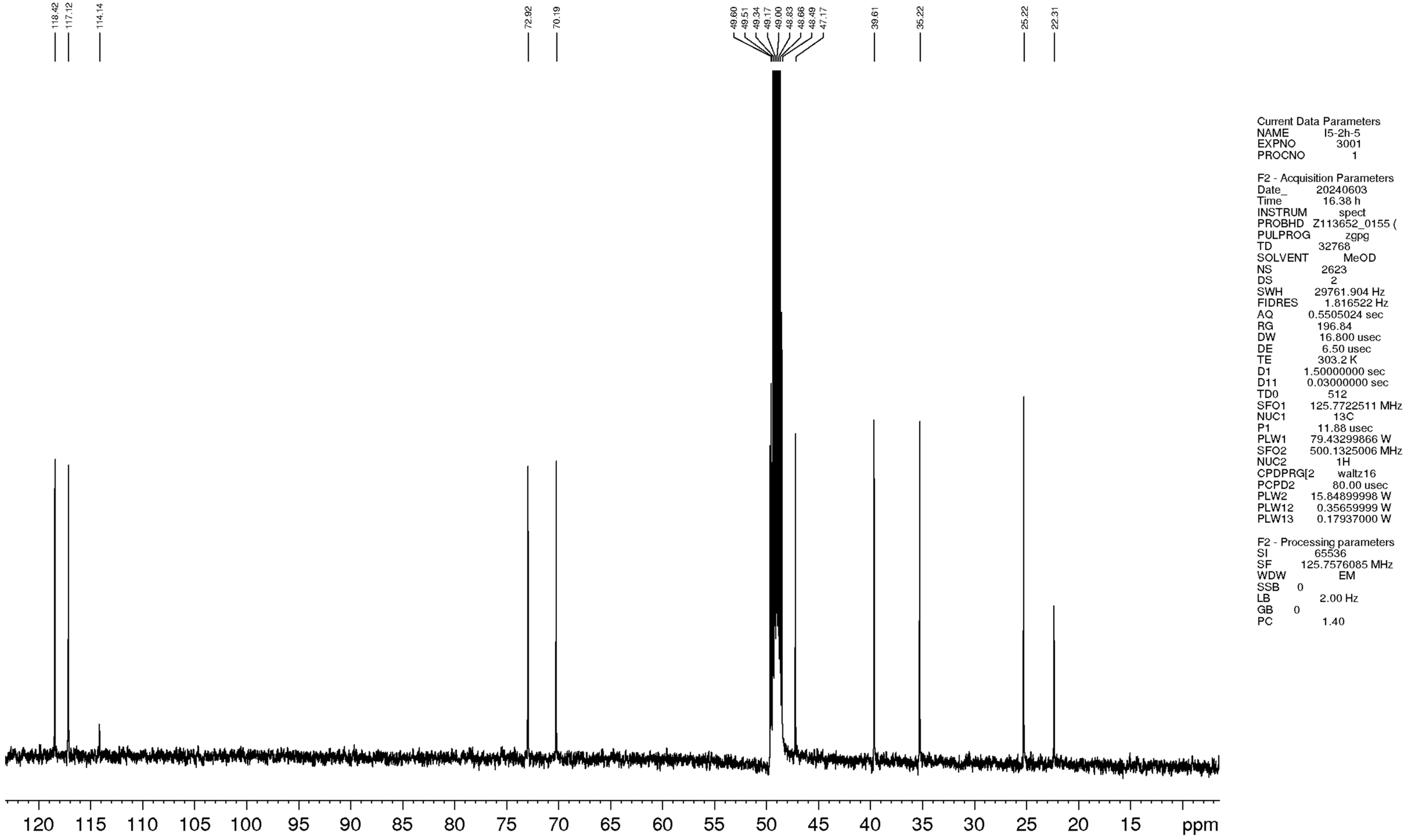

Figure S35. DEPT NMR spectrum (500 MHz, CD_3OD) of **10**

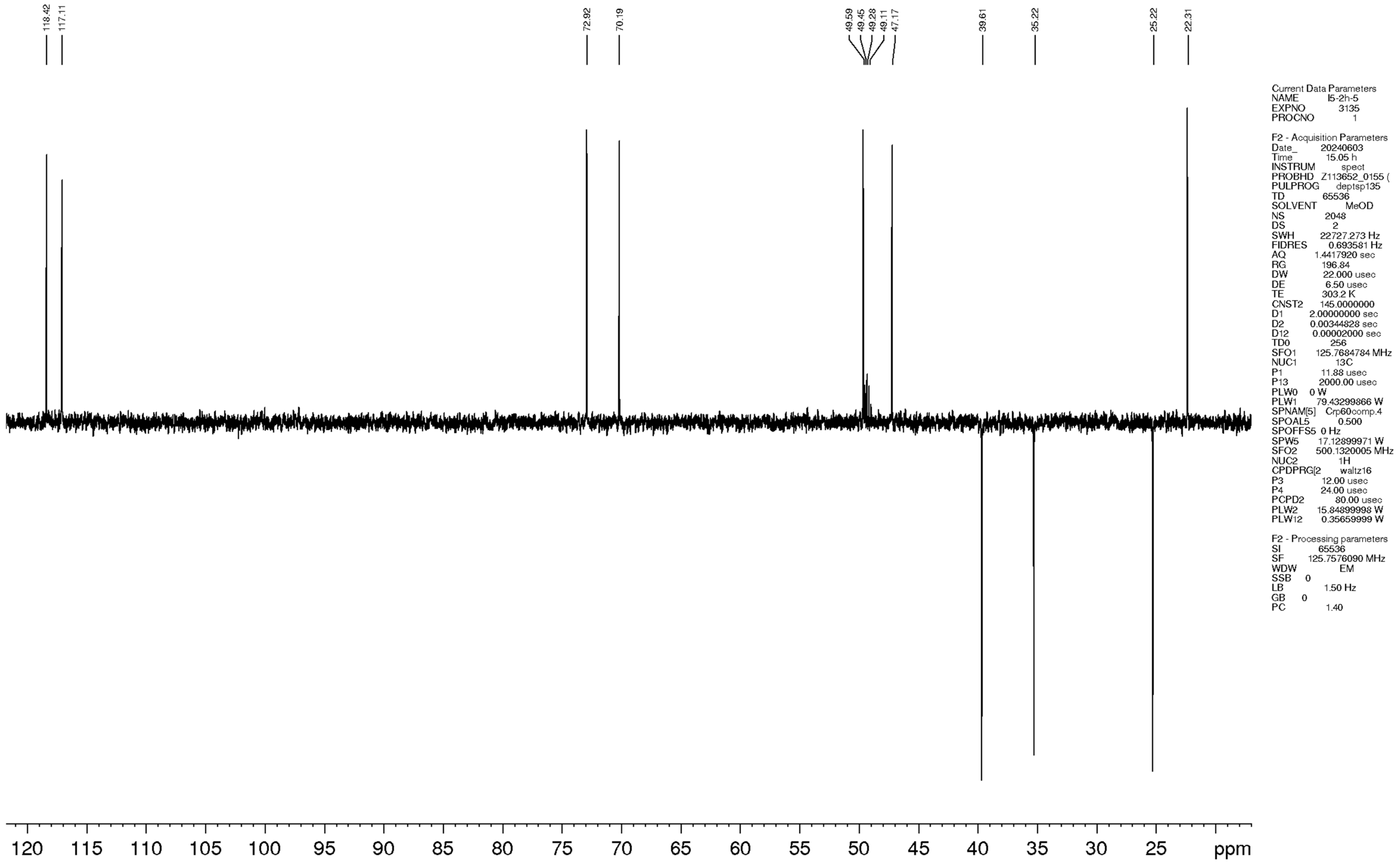

Figure S36. ^{1}H NMR spectrum (500 MHz, acetone-d6) of **11**

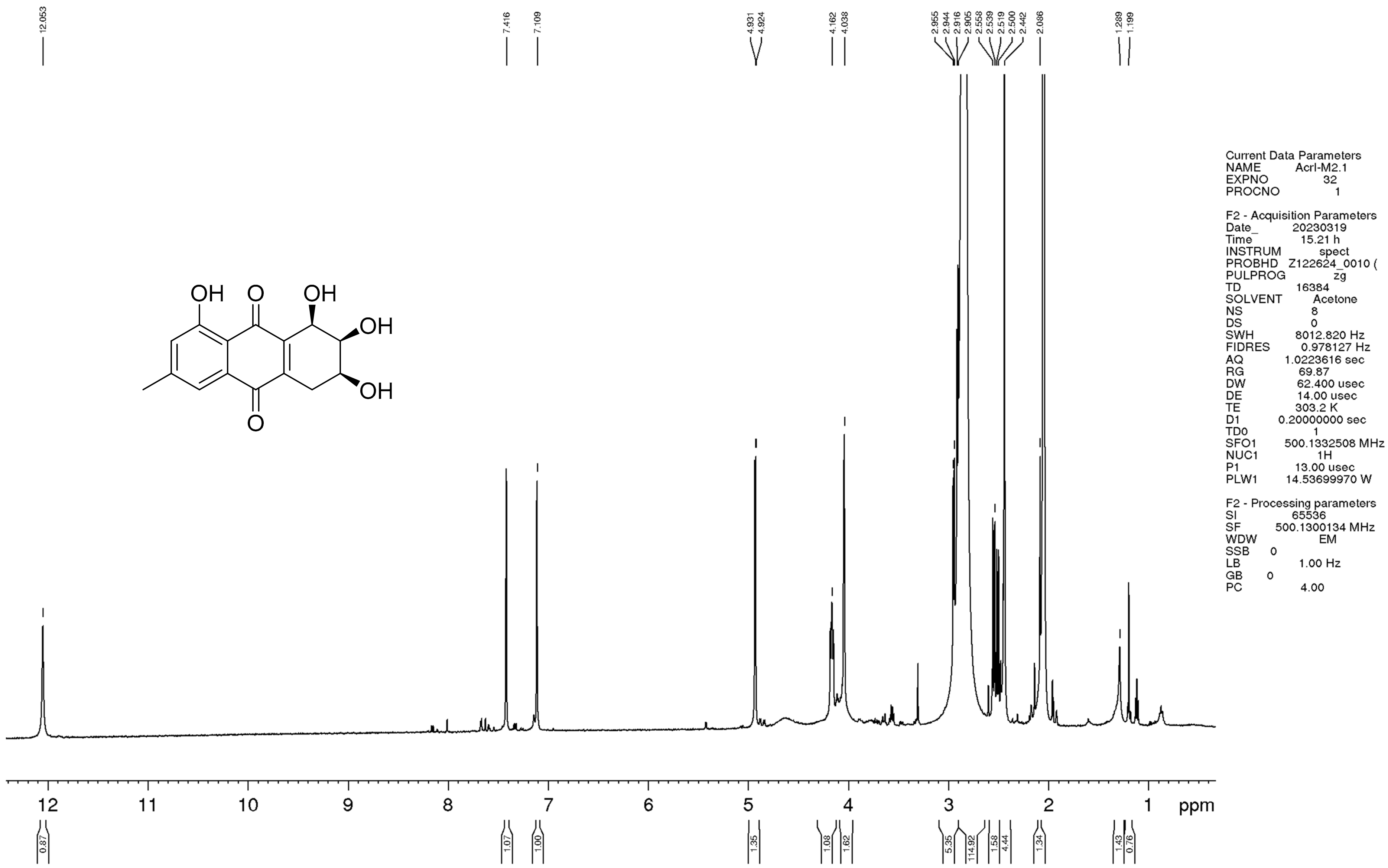

Figure S37. ^{13}C NMR spectrum (500 MHz, acetone-d6) of **11**

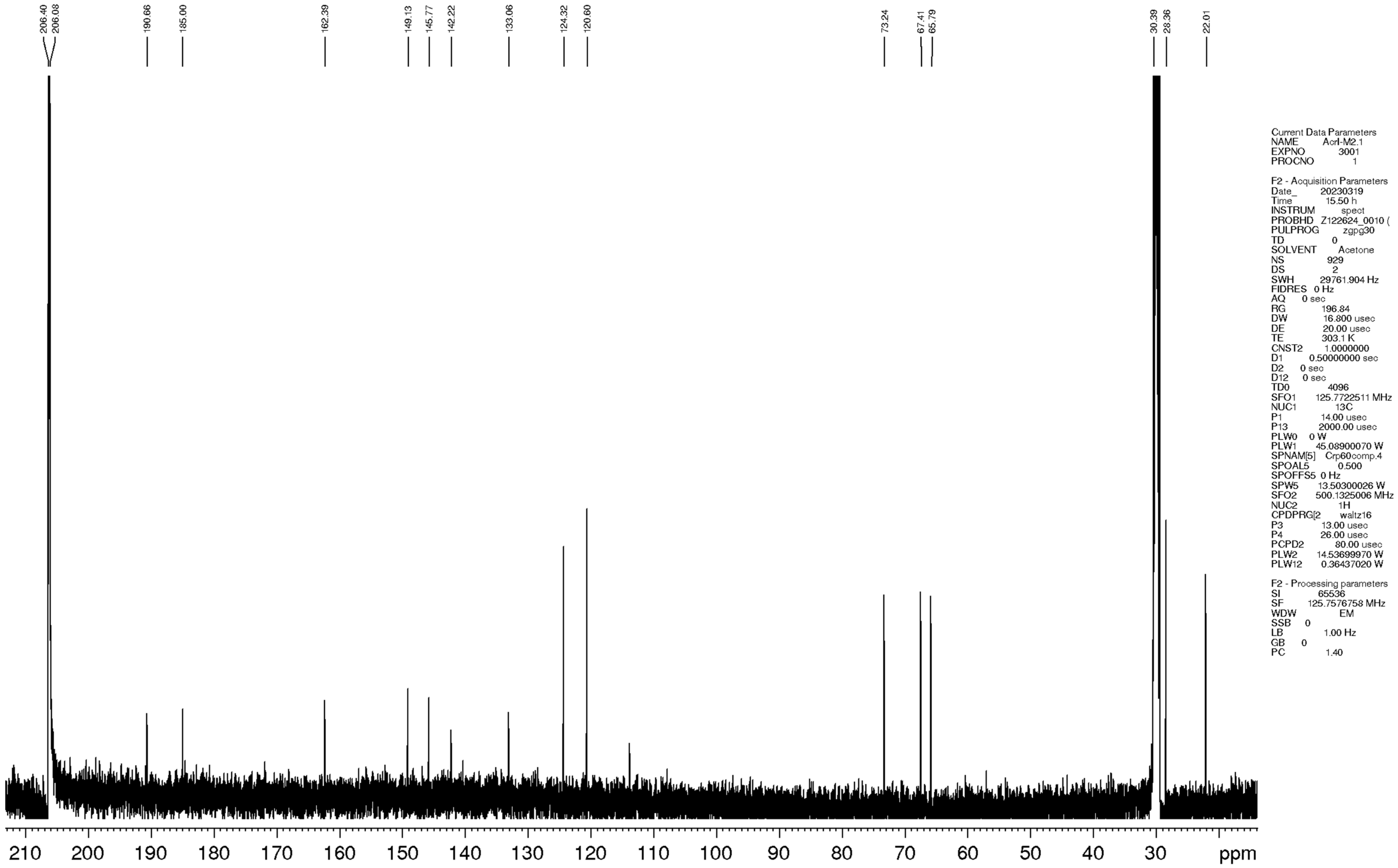

Figure S38. DEPT NMR spectrum (500 MHz, acetone-d6) of **11**

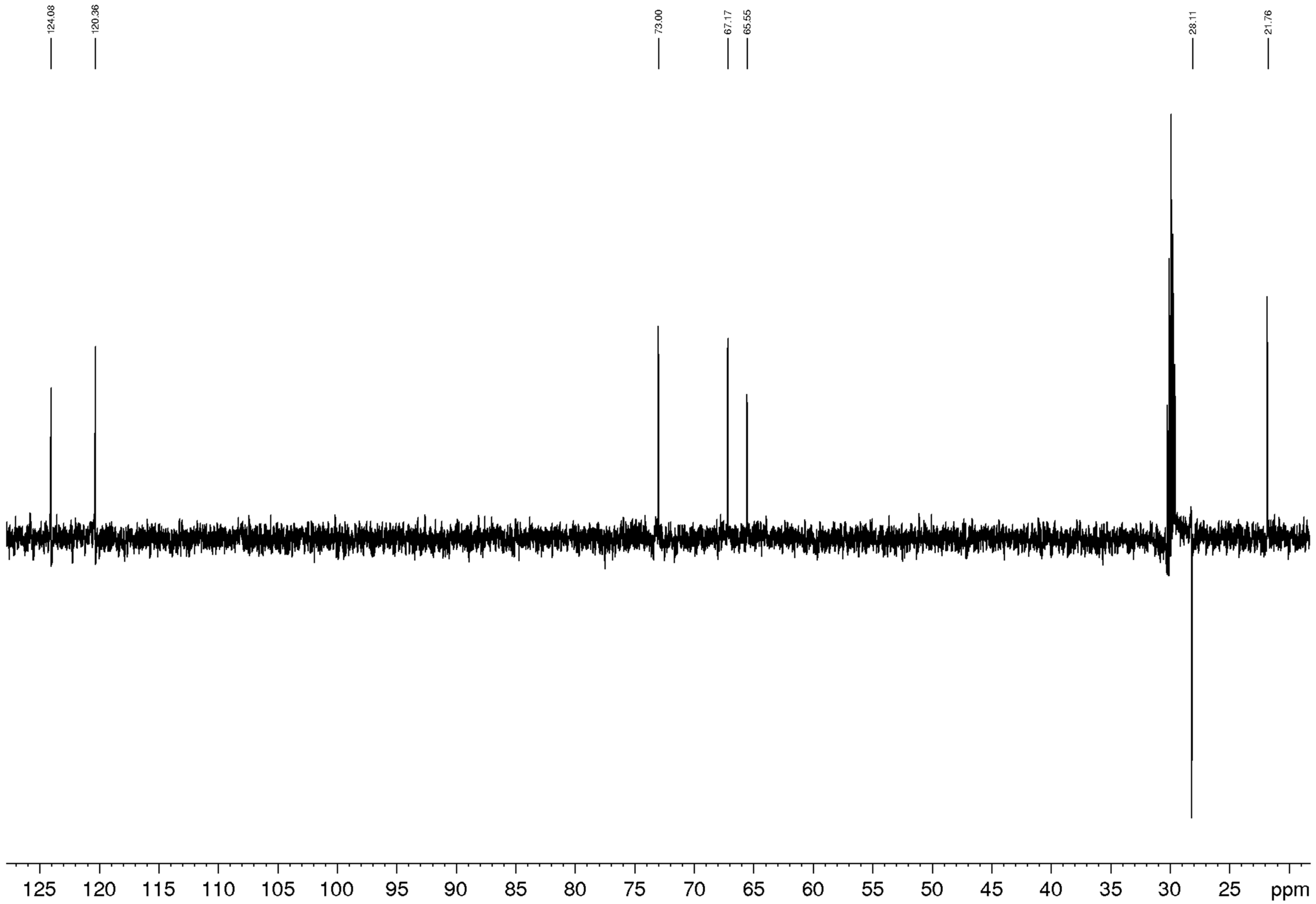

Current Data Parameters
NAME Acrl-M2.1
EXPNO 3135
PROCNO 1

F2 - Acquisition Parameters
Date_ 20230319
Time 17.06 h
INSTRUM spect
PROBHD Z122624_0010 (
PULPROG deptsp135
TD 65536
SOLVENT Acetone
NS 707
DS 2
SWH 22727.273 Hz
FIDRES 0.693581 Hz
AQ 1.4417920 sec
RG 196.84
DW 22.000 usec
DE 20.00 usec
TE 303.2 K
CNST2 145.0000000
D1 2.00000000 sec
D2 0.00344828 sec
D12 0.00002000 sec
TD0 256
SFO1 125.7684784 MHz
NUC1 13C
P1 14.00 usec
P13 2000.00 usec
PLW0 0 W
PLW1 45.08900070 W
SPNAM[5] Crp60comp.4
SPOAL5 0.500
SPOFFS5 0 Hz
SPW5 13.50300026 W
SFO2 500.1320005 MHz
NUC2 1H
CPDPRG[2 waltz16
P3 13.00 usec
P4 26.00 usec
PCPD2 80.00 usec
PLW2 14.53699970 W
PLW12 0.36437020 W

F2 - Processing parameters
SI 65536
SF 125.7577060 MHz
WDW EM
SSB 0
LB 1.50 Hz
GB 0
PC 1.40

Figure S39. UV spectrum of **1**

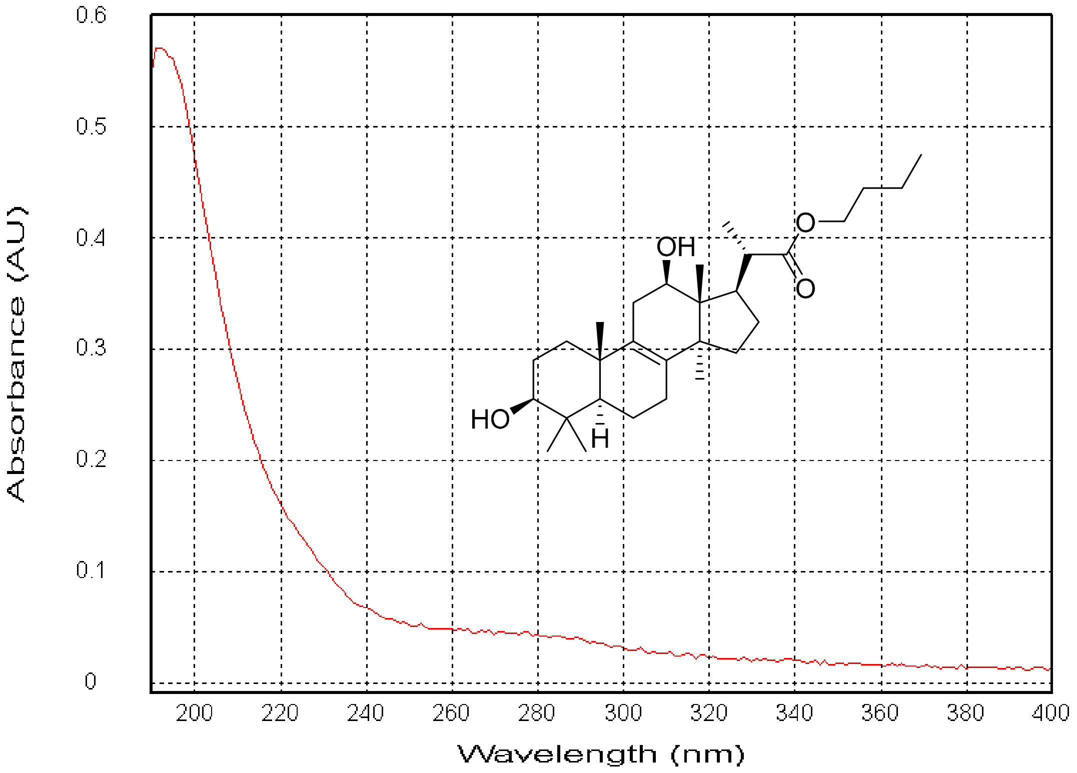

Figure S40. ECD spectrum of **1**

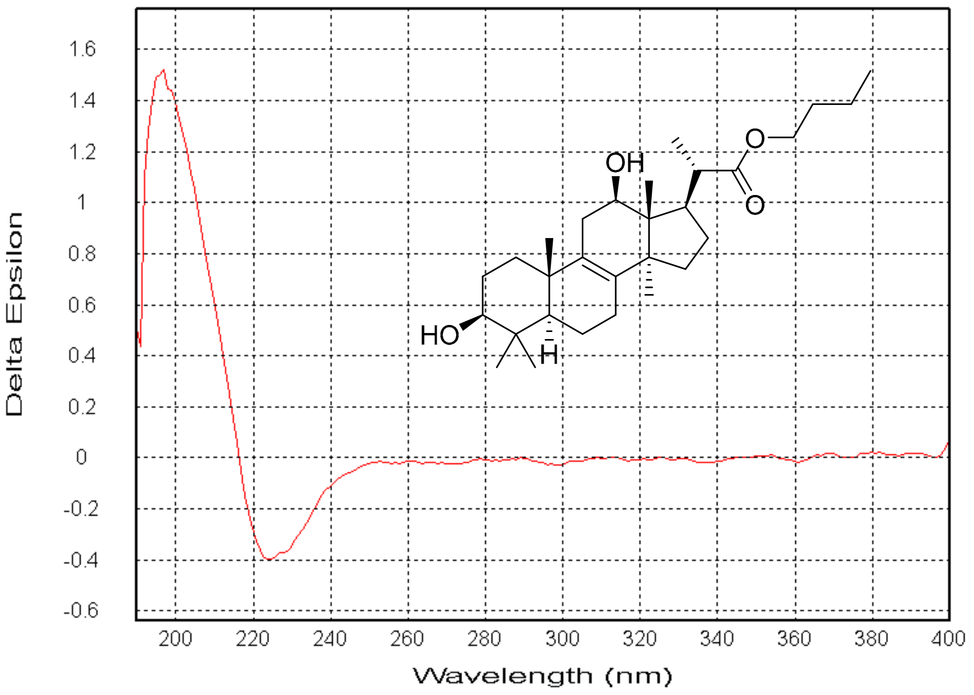

Figure S41. UV spectrum of 3

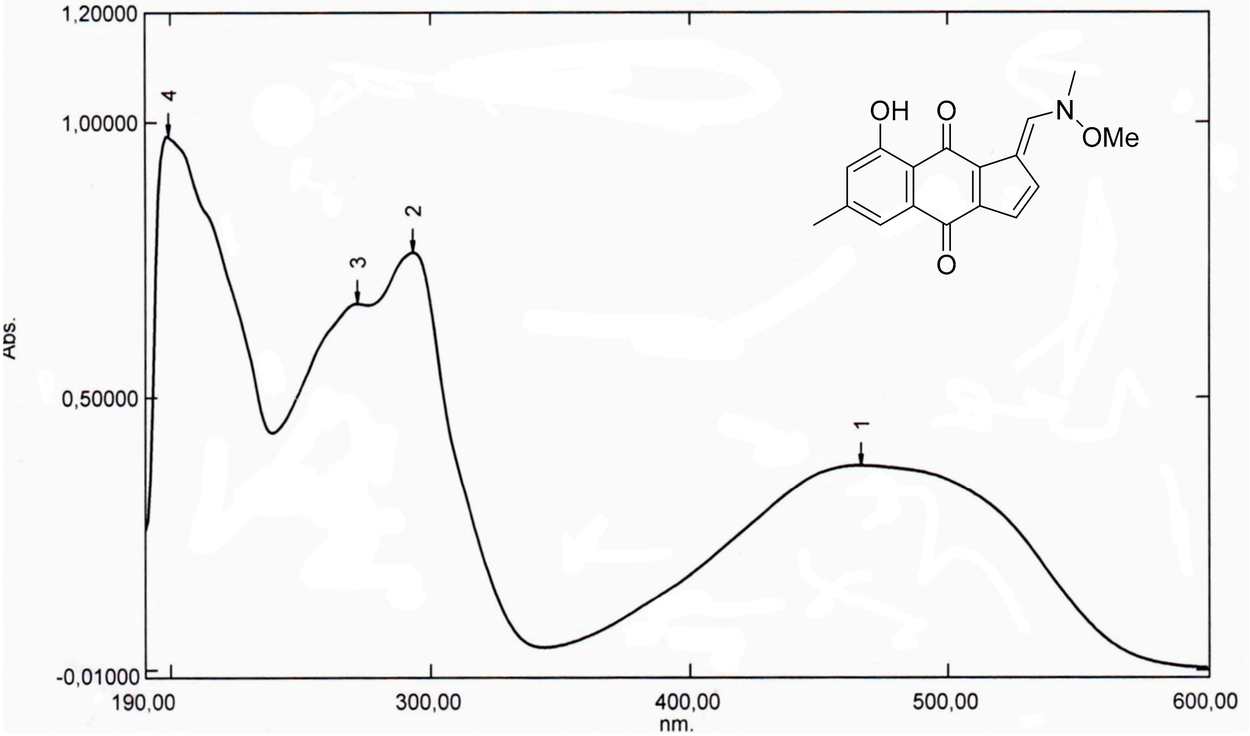

Figure S42. (+) ESI MS of **1**

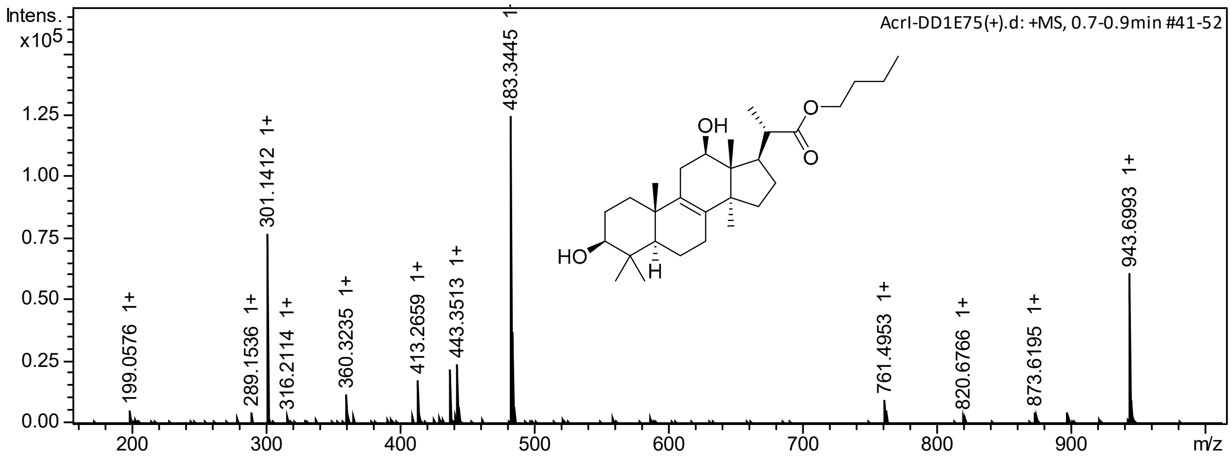

Figure S43. (+) ESI MS of **2**

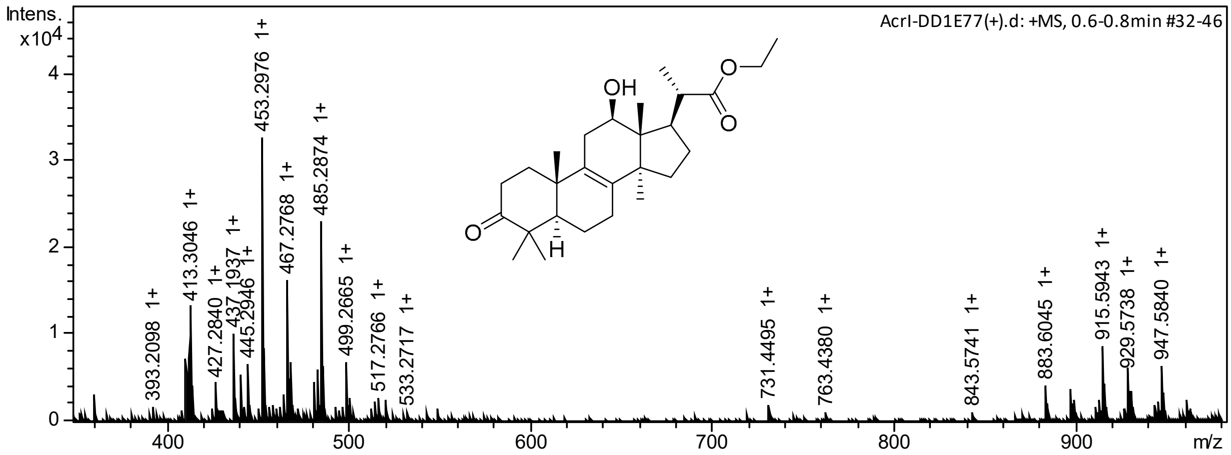

Figure S44. (-) ESI MS of **3**

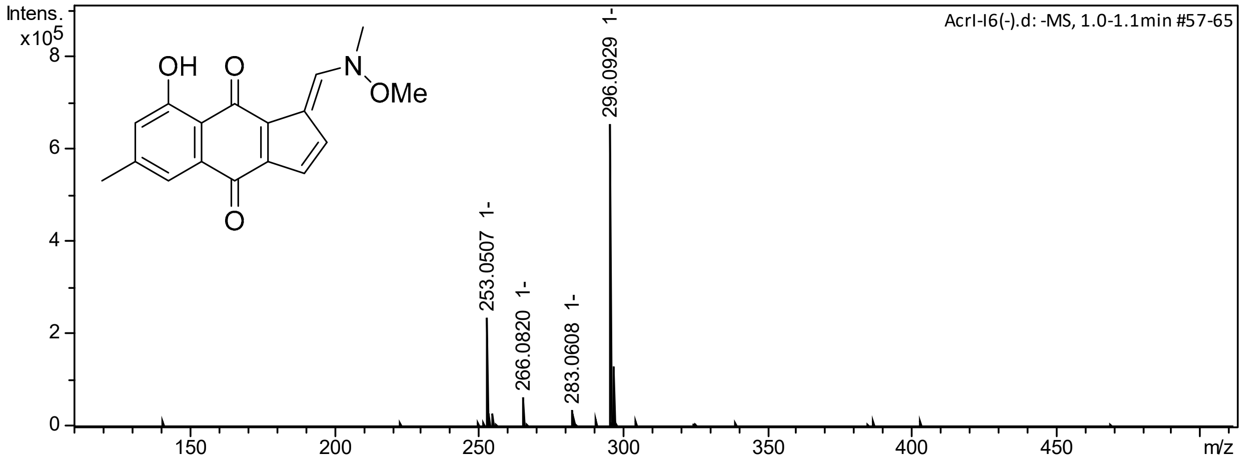

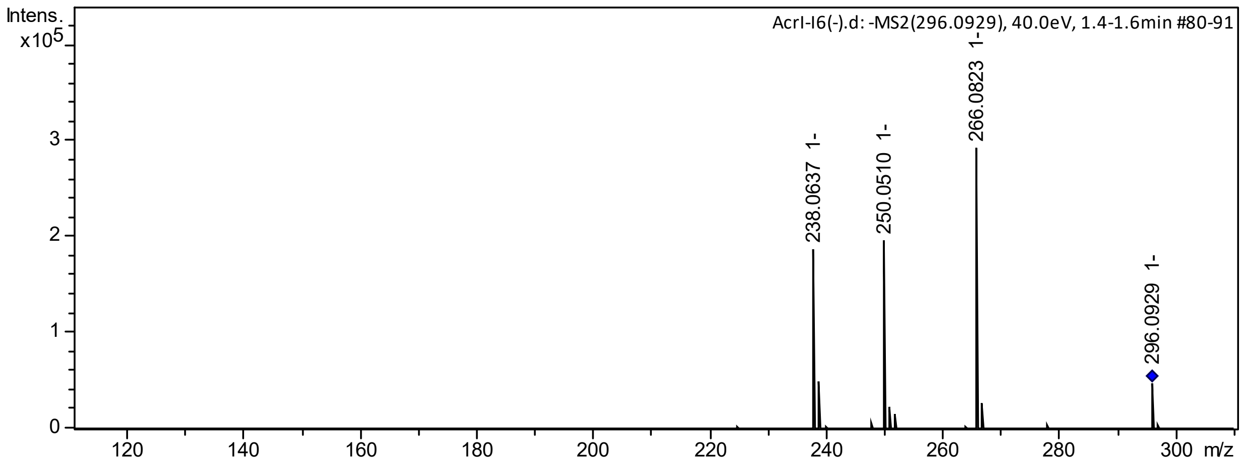

Figure S45. (-) ESI MS of **5**

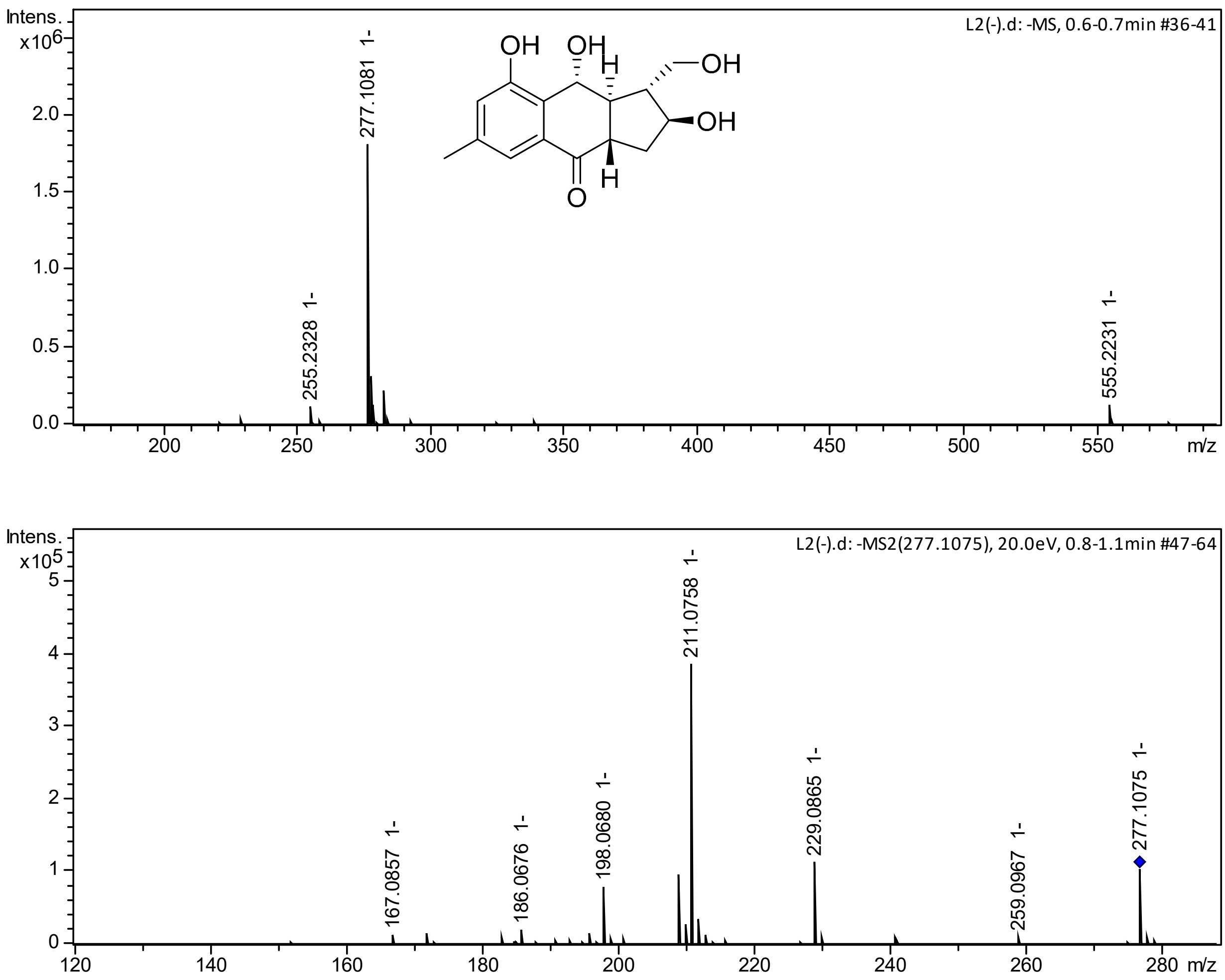

Figure S46. (-) ESI MS of **6**

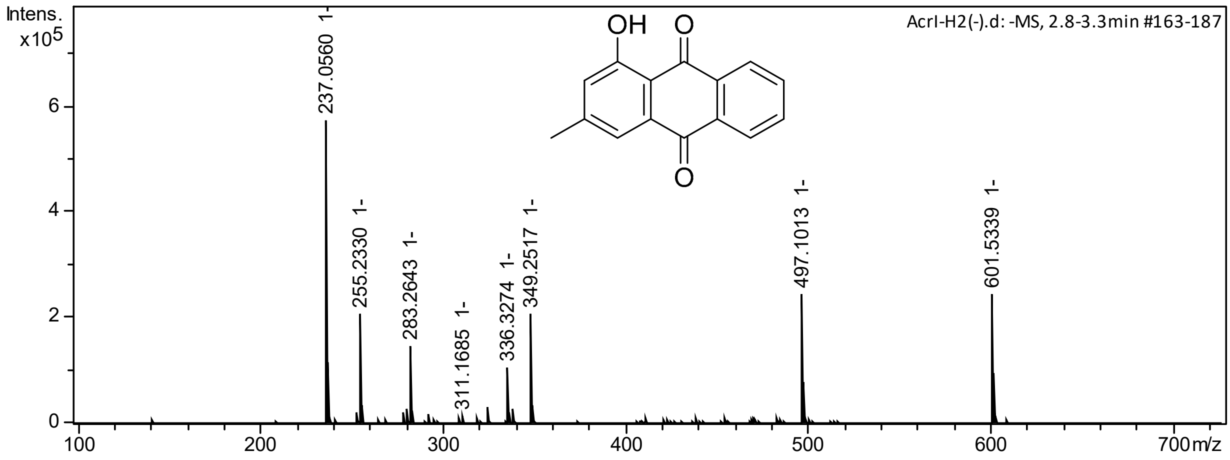

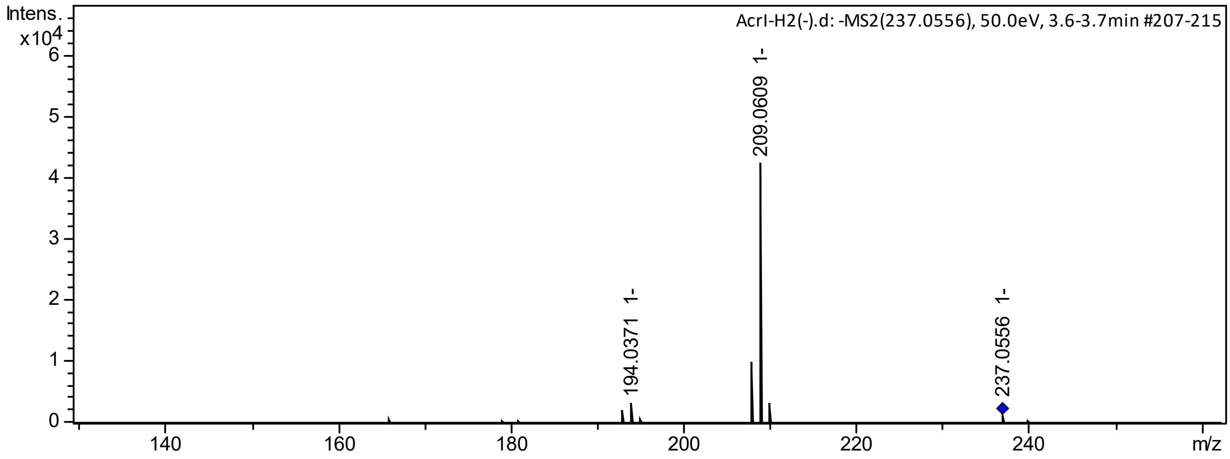

Figure S47. (-) ESI MS of **7**

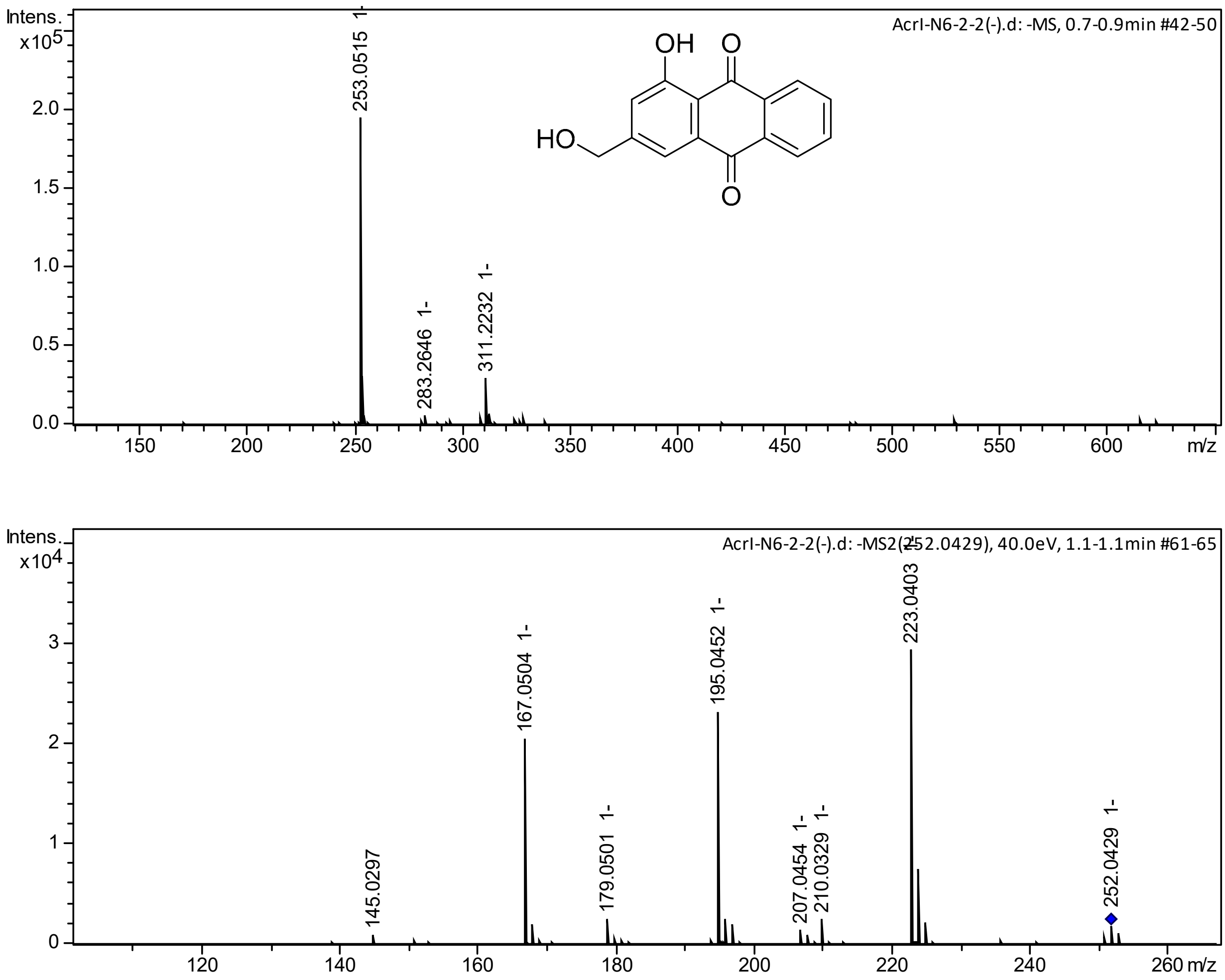

Figure S48. (-) ESI MS of **8**

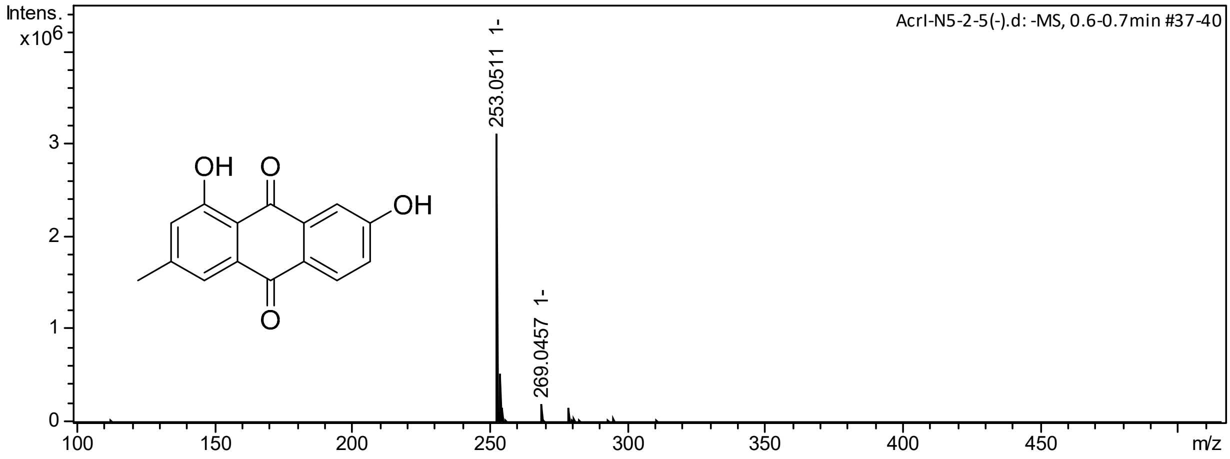

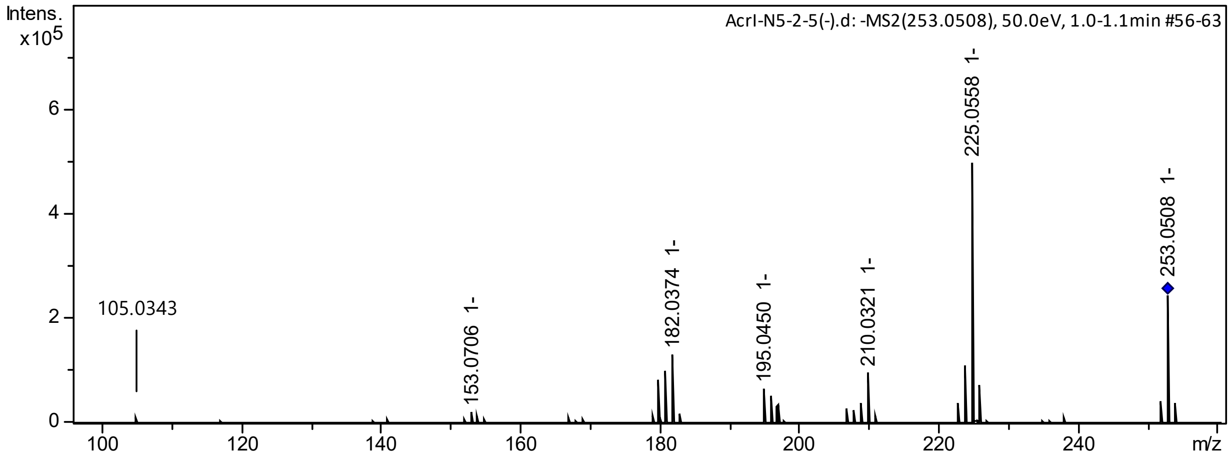

Figure S49. (-) ESI MS of **9**

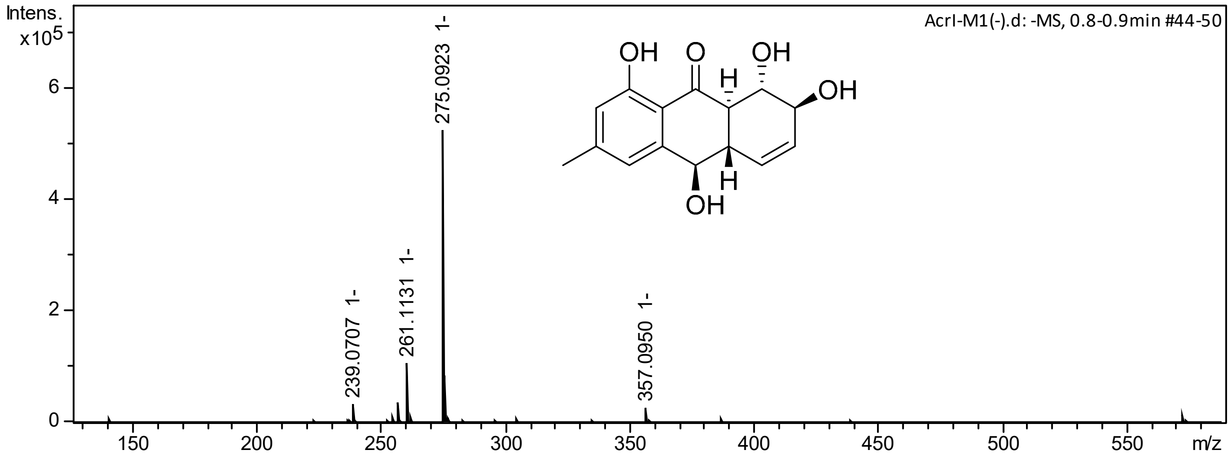

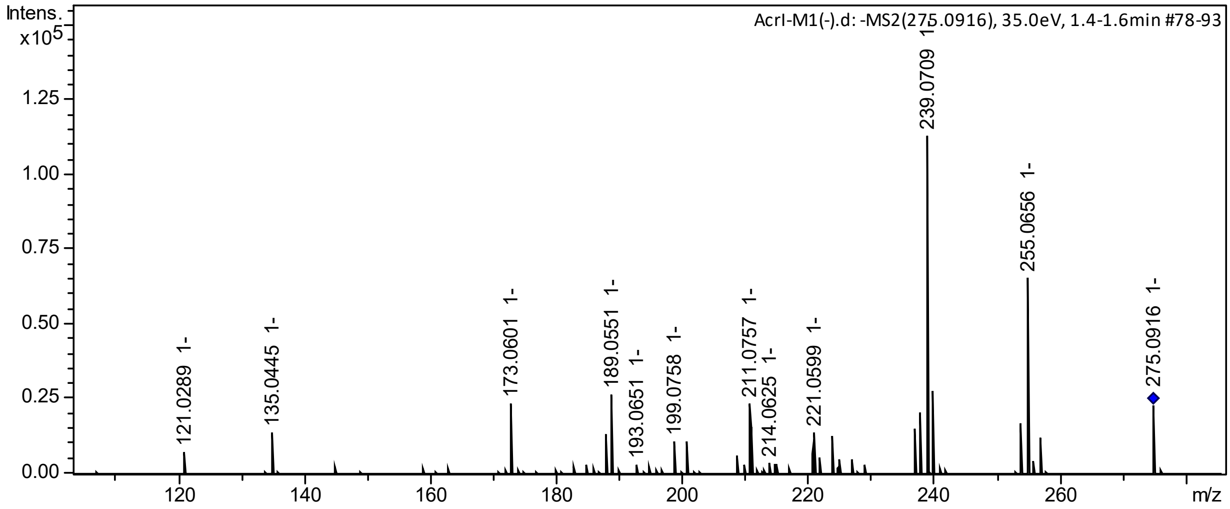

Figure S50. (-) ESI MS of **10**

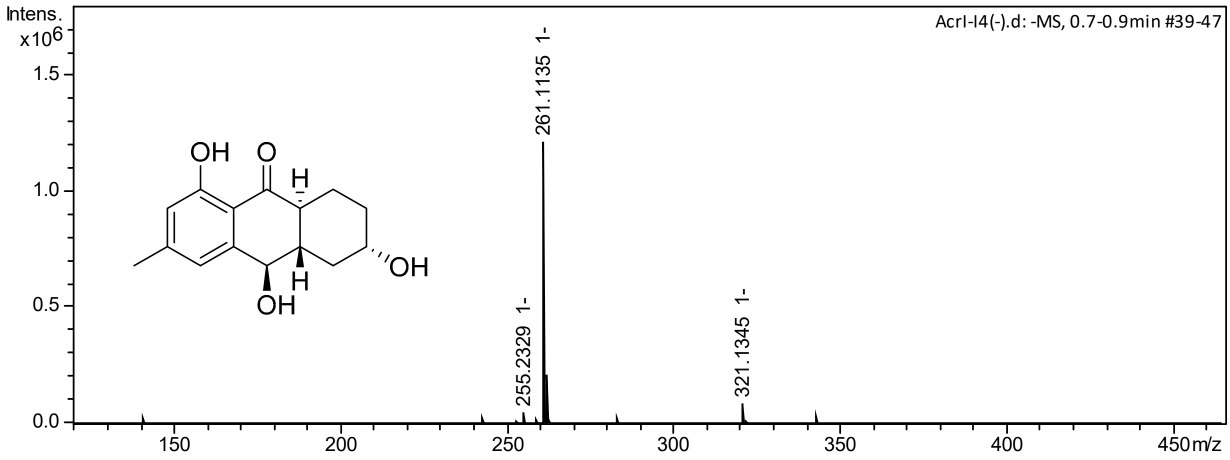

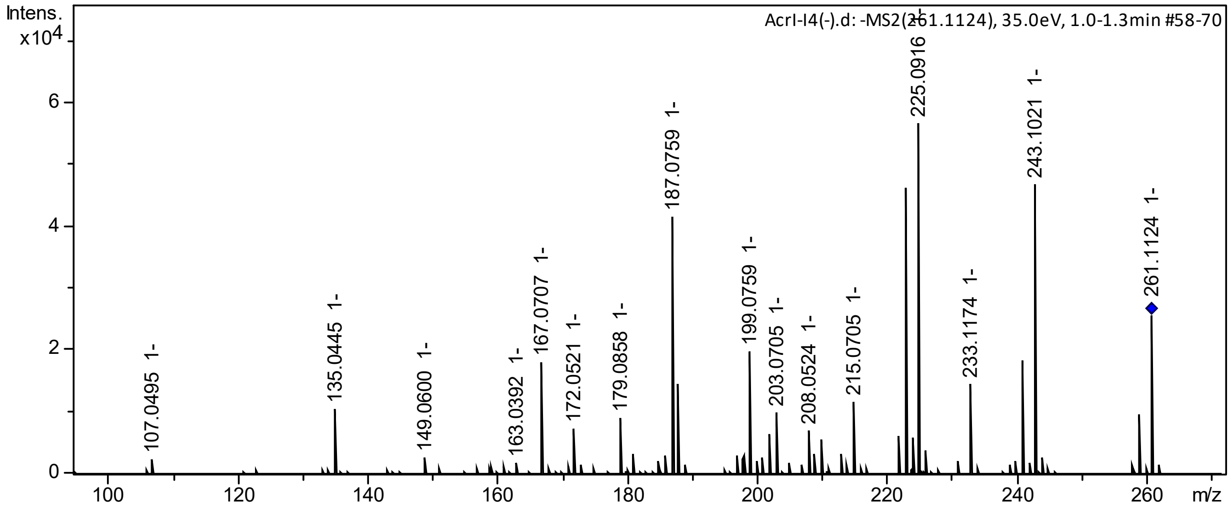

Figure S51. (-) ESI MS of **11**

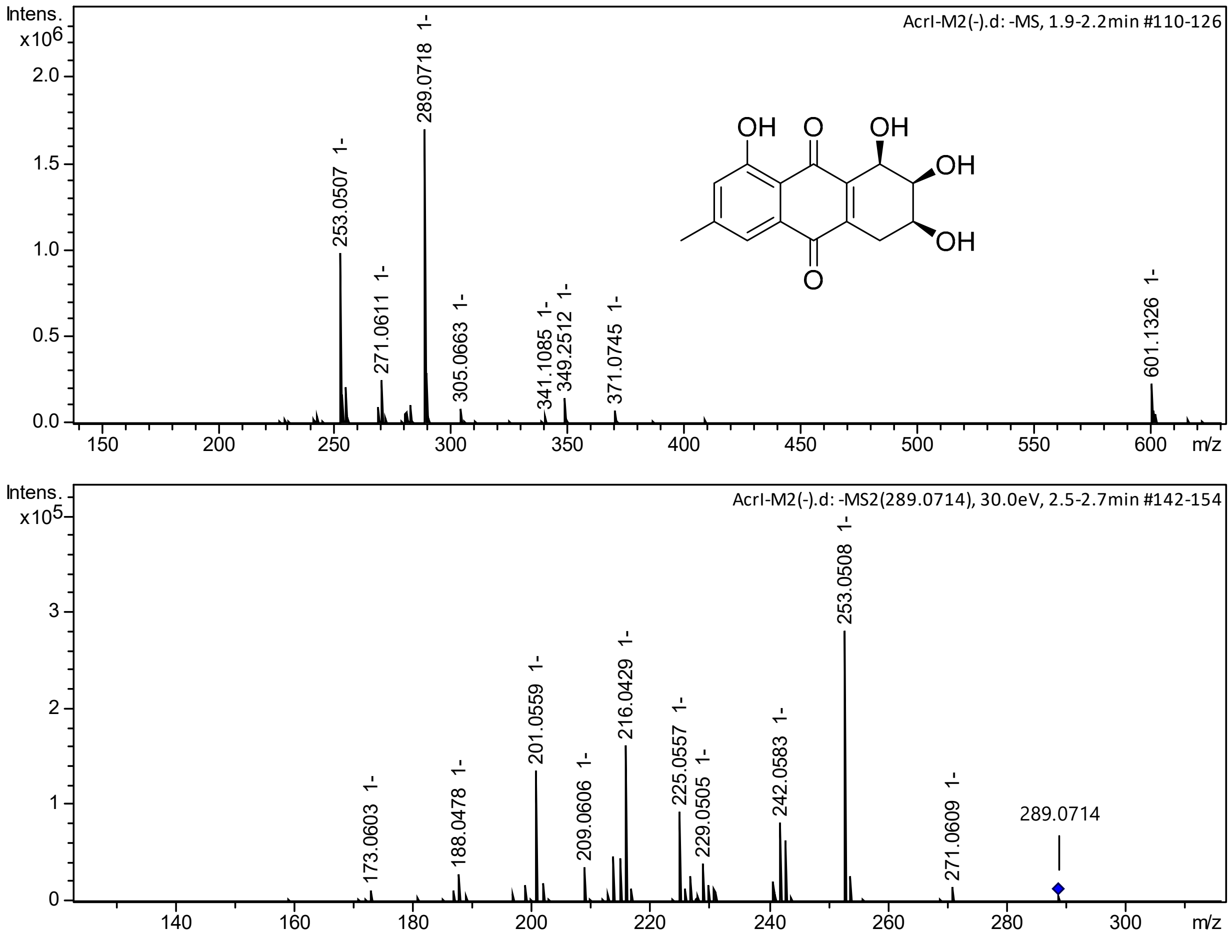

Figure S52. (-) ESI MS of **4**

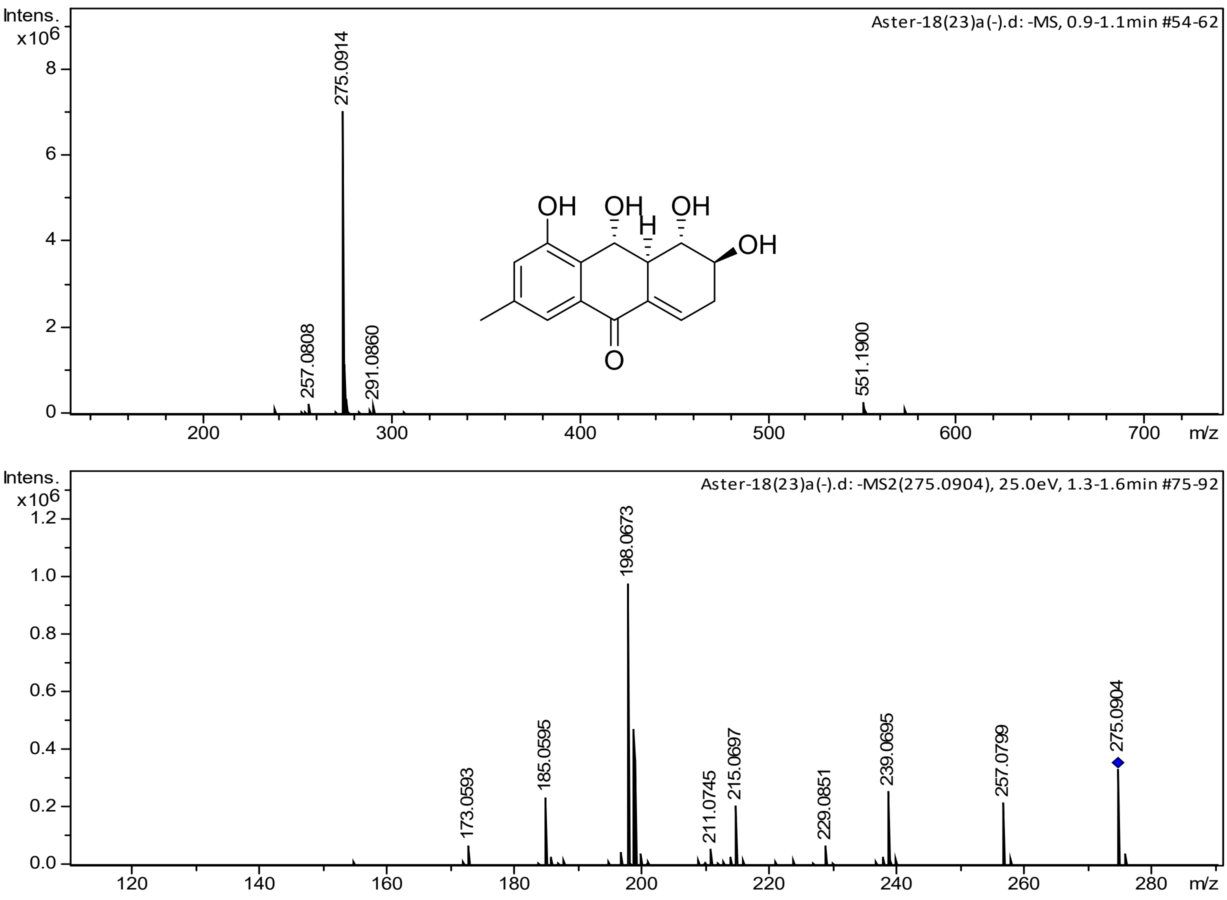

Table S1. The pentanorlanostane type compounds

Structure	Name	Source	Ref
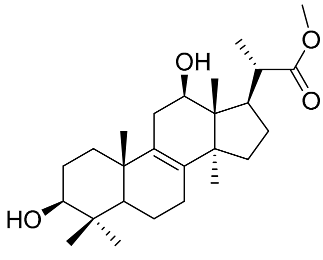	curvalarol A	soil fungus *Curvularia borreriae* HS-FG-237	[1]
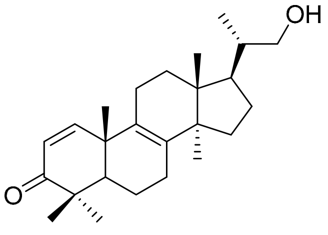	curvalarol B	soil fungus *Curvularia borreriae* HS-FG-237	[1]
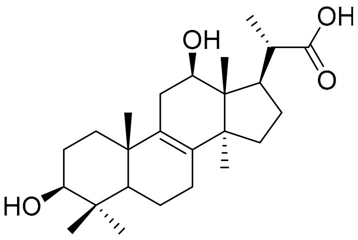	dendryphiellide A	marine fungus *Paradendryphiella salina* PC 362H	[2]
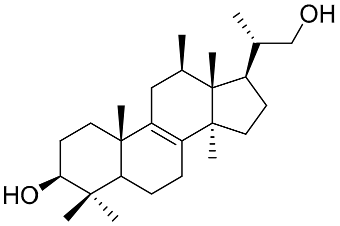	23,24,25,26,27-pentanorlanost-8-ene-3β,22-diol	soil fungus *Curvularia borreriae* HS-FG-237<br>entomopathogenic fungus *Verticillium lecanii*<br>dematiaceous fungus *Cladosporium* sp. IFM 49189	[1]<br>[3]<br>[4]
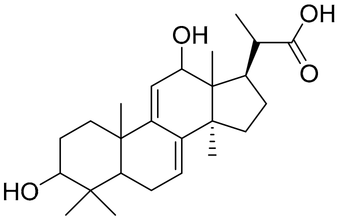	pentanorlanost-7,9(11)-diene asid	entomopathogenic fungus *Verticillium lecanii*	[3]
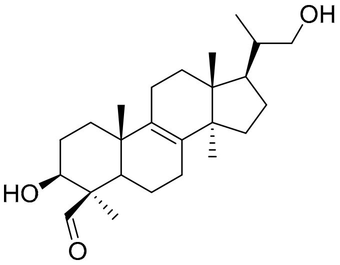	cladosporide A	dematiaceous fungus *Cladosporium* sp. IFM 49189	[4]

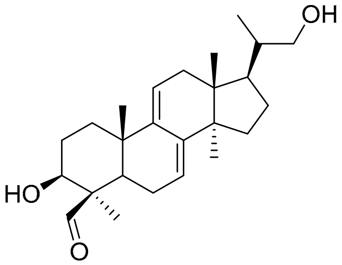	cladosporide B		
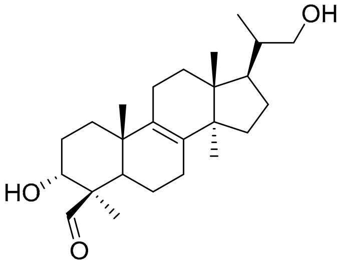	cladosporide C		
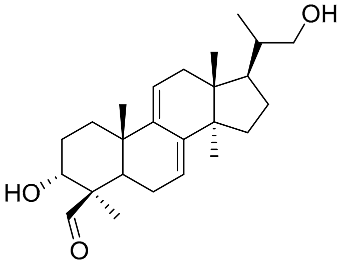	cladosporide D		
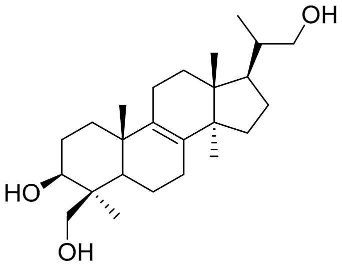	dihydrocladosporide A		
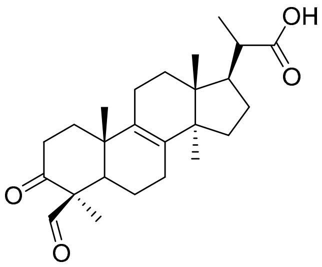	3,30-dioxo23,24,25,26,27-pentanorlanost-8-en-22-oic acid		
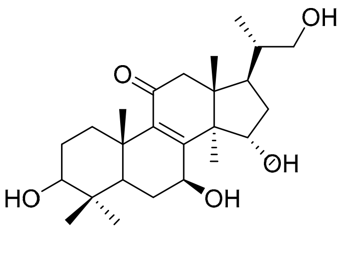	ganosineniol A (**1**)	medicinal fungus *Ganoderma sinense*	[5]

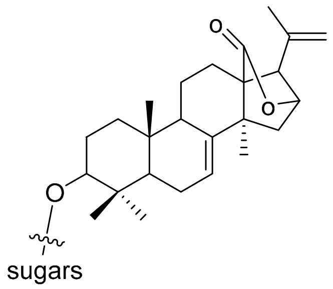	cucumarioside I_4	sea cucumber *Eupentacta fraudatrix*	[6]
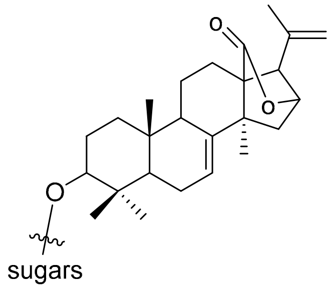	cucumarioside A_{10}	sea cucumber *Eupentacta fraudatrix*	[7]
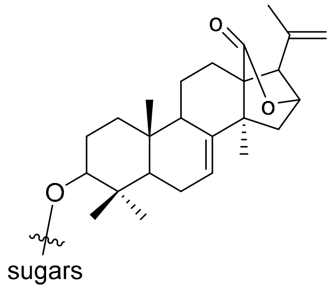	cucumarioside G_2	sea cucumber *Eupentacta fraudatrix*	[8]

References

1. Wang, X.J.; Xue, C.Y.; Li, J.S.; Xiang, W.S.; Qi, H.; Xi, F.Y.; Wang, J.D. Two new pentanorlanostane metabolites from a soil fungus *Curvularia borreriae* strain HS-FG-237. *J. Antibiot.* **2013**, *66*, 735-737. 10.1038/ja.2013.85
2. Dezaire, A.; Marchand, C.H.; Vallet, M.; Ferrand, N.; Chaouch, S.; Mouray, E.; Larsen, A.K.; Sabbah, M.; Lemaire, S.D.; Prado, S., *et al.* Secondary metabolites from the culture of the marine-derived fungus *Paradendryphiella salina* PC 362H and evaluation of the anticancer activity of its metabolite hyalodendrin. *Mar. Drugs* **2020**, *18*. 10.3390/md18040191
3. Grove, J.F. 23,24,25,26,27-pentanorlanost-8-en-3β,22-diol from *Verticillium lecanii*. *Phytochemistry* **1984**, *23*, 1721-1723.
4. Hosoe, T.; Okamoto, S.; Nozawa, K.; Kawai, K.I.; Okada, K.; De Campos Takaki, G.M. New pentanorlanostane derivatives, cladosporide B-D, as characteristic antifungal agents against *Aspergillus fumigatus*, isolated from *Cladosporium* sp. *J. Antibiot.* **2001**, *54*, 747-750. 10.7164/antibiotics.54.747
5. Liu, J.Q.; Wang, C.F.; Li, Y.; Luo, H.R.; Qiu, M.H. Isolation and bioactivity evaluation of terpenoids from the medicinal fungus *Ganoderma sinense*. *Planta Med.* **2012**, *78*, 368-376. 10.1055/s-0031-1280441
6. Silchenko, A.S.; Kalinovsky, A.I.; Avilov, S.A.; Andryjaschenko, P.V.; Dmitrenok, P.S.; Martyyas, E.A.; Kalinin, V.I. Triterpene glycosides from the sea cucumber *Eupentacta fraudatrix*. Structure and biological action of cucumariosides I_1, I_3, I_4, three new minor disulfated pentaosides. *Nat. Pro. Comm.* **2013**, *8*, 1053-1058. 10.1177/1934578x1300800805
7. Silchenko, A.S.; Kalinovsky, A.I.; Avilov, S.A.; Andryjaschenko, P.V.; Dmitrenok, P.S.; Martyyas, E.A.; Kalinin, V.I. Triterpene glycosides from the sea cucumber *Eupentacta fraudatrix*. Structure and cytotoxic action of cucumariosides A_2, A_7, A_9, A_{10}, A_{11}, A_{13} and A_{14}, sevennew minor non-sulfated tetraosides and an aglycone with an uncommon 18-hydroxy group. *Nat. Pro. Comm.* **2012**, *7*, 845-852. 10.1177/1934578x1200700710

8. Avilov, S.A.; Kalinin, V.I.; Makarieva, T.N.; Stonik, V.A.; Kalinovsky, A.I.; Rashkes, Y.W.; Milgrom, Y.M. Structure of cucumarioside G_2, a novel nonholostane glycoside from the sea cucumber *Eupentacta fraudatrix*. *J. Nat. Prod.* **1994**, *57*, 1166-1171. 10.1021/np50110a007

Table S2. The HPLC MS data of compounds 1 and 2

N	RT, min	Meas-ured m/z	MF	Calculated m/z	Error, ppm	Name and identi-fication lvl	Structure
1	17.9	483.3389	$C_{29}H_{48}O_4$	483.3445 [M+Na]+	-11.6	curvalarol C (**1**)	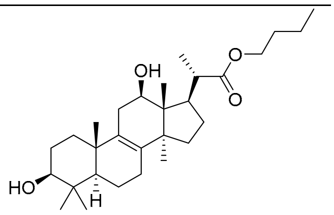
2	17.3	431.3124	$C_{27}H_{42}O_4$	431.3156 [M+H]+	-7.4	curvalarol D (**2**)	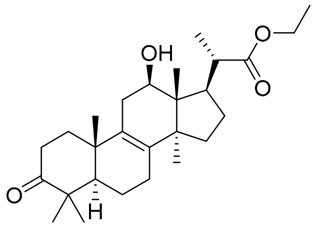

Figure S53. Morphological characters of *Asteromyces cruciatus* KMM 4696.

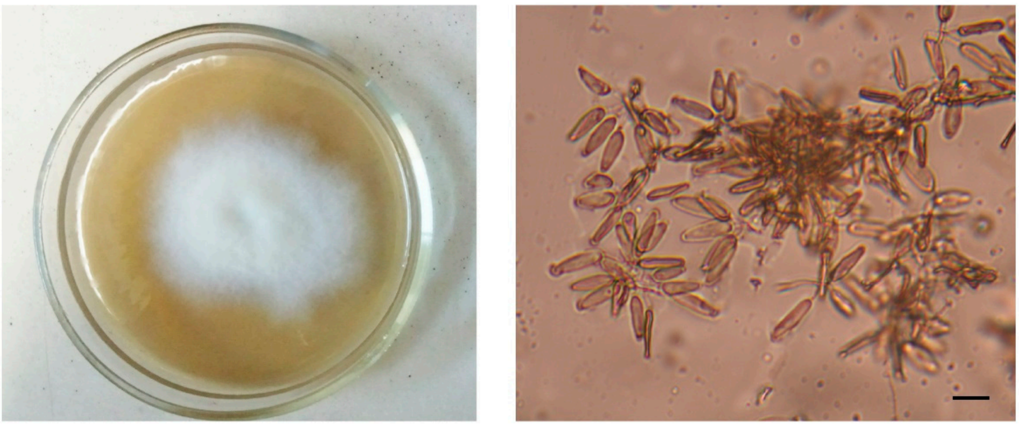

A B

A. Colony after 7 d at 25 °C on MEA. **B**. Conidia. Scale bar=10μm.

Figure S54. Sequence variation of concatenated ITS-LSU-*tef1* between KMM 4696 and ex-types of the genera *Asteromyces*, *Stemphylium* and *Paradendryphiella*. ITS, LSU and *tef1* are shown in green, yellow and blue, respectively. Identical nucleotides are shown as asterisk.

	35	36	38	43	55	126	372	373	401	414	452	453	454	462	513	623	706	808	831	911	1275	1279	1290	1299	1300	1302	1318	1319
Asteromyces cruciatus CBS 171.63^{T}	T	C	C	G	T	C	T	T	C	G	C	T	G	A	T	T	C	A	A	T	T	T	T	G	C	T	C	G
Asteromyces cruciatus KMM 4696	*	*	*	*	*	*	*	*	*	*	*	*	*	*	*	*	*	*	*	*	*	*	*	*	*	*	*	*
Paradendryphiella salina CBS 302.84^{T}	*	*	*	*	*	*	C	*	T	*	*	*	*	*	C	*	T	G	C	C	*	A	*	A	T	*	T	A
Stemphylium vesicarium CBS 191.86	C	*	*	A	*	A	C	*	T	A	T	G	A	T	*	C	T	G	C	C	C	A	C	*	*	C	T	A
Stemphylium botryosum CBS 714.68^{T}	C	T	T	A	C	A	C	C	T	A	T	G	A	T	*	*	T	G	C	C	C	A	C	*	*	C	*	A

	1320	1321	1326	1335	1336	1337	1338	1352	1367	1388	1389	1392	1393	1402	1403	1408	1415	1435	1436	1438	1439	1447	1448	1457	1460	1461	1464
Asteromyces cruciatus CBS 171.63^{T}	G	C	T	T	T	T	T	C	T	G	T	T	T	C	C	C	G	T	T	G	G	A	A	G	T	C	C
Asteromyces cruciatus KMM 4696	*	*	*	*	*	*	*	*	*	*	*	*	*	*	*	*	*	*	*	*	*	*	*	*	*	*	*
Paradendryphiella salina CBS 302.84^{T}	T	*	*	*	*	*	*	T	*	*	*	C	C	*	T	T	A	*	*	C	C	G	*	*	*	*	T
Stemphylium vesicarium CBS 191.86	T	G	A	C	A	A	A	T	C	T	C	C	C	T	*	*	A	C	G	A	T	G	G	A	C	T	T
Stemphylium botryosum CBS 714.68^{T}	T	*	A	C	A	A	A	T	C	*	C	C	C	*	*	*	A	C	G	A	T	G	G	A	C	T	*
